# High conductance values in π-folded molecular junctions

**DOI:** 10.1038/ncomms15195

**Published:** 2017-05-18

**Authors:** Marco Carini, Marta P. Ruiz, Imanol Usabiaga, José A. Fernández, Emilio J. Cocinero, Manuel Melle-Franco, Ismael Diez-Perez, Aurelio Mateo-Alonso

**Affiliations:** 1 POLYMAT, University of the Basque Country UPV/EHU, Avenida de Tolosa 72, E-20018 Donostia-San Sebastian, Spain; 2 Department of Materials Science and Physical Chemistry, Institute of Theoretical and Computational Chemistry, University of Barcelona, Martí I Franquès 1, 08028 Barcelona, Spain; 3 Institute for Bioengineering of Catalonia (IBEC), Martí I Franquès 1, 08028 Barcelona, Spain; 4 Departamento de Química Física, Facultad de Ciencia y Tecnología, Universidad del País Vasco (UPV/EHU), Apartado 644, E-48080 Bilbao, Spain; 5 CICECO—Aveiro Institute of Materials, Department of Chemistry, University of Aveiro, 3810-193 Aveiro, Portugal; 6 Centro ALGORITMI, 4710-057 Braga, Portugal; 7 Ikerbasque, Basque Foundation for Science, Bilbao, Spain

## Abstract

Folding processes play a crucial role in the development of function in biomacromolecules. Recreating this feature on synthetic systems would not only allow understanding and reproducing biological functions but also developing new functions. This has inspired the development of conformationally ordered synthetic oligomers known as foldamers. Herein, a new family of foldamers, consisting of an increasing number of anthracene units that adopt a folded sigmoidal conformation by a combination of intramolecular hydrogen bonds and aromatic interactions, is reported. Such folding process opens up an efficient through-space charge transport channel across the interacting anthracene moieties. In fact, single-molecule conductance measurements carried out on this series of foldamers, using the scanning tunnelling microscopy-based break-junction technique, reveal exceptionally high conductance values in the order of 10^−1^ *G*_0_ and a low length decay constant of 0.02 Å^−1^ that exceed the values observed in molecular junctions that make use of through-space charge transport pathways.

## Abstract (web summary)

Foldamers are synthetic oligomers that adopt folded conformations through non-covalent intramolecular interactions. Here, Carini *et al*. describe a family of foldamers with a large number of anthracene units that are able to transport charge efficiently at the single-molecule level.

The high level of performance of proteins and nucleic acids in terms of function is not only the result of their chemical (primary) structure but also of the implicit non-covalent intramolecular interactions between specific units in their sequence that promote folding into a well-defined (secondary and tertiary) three-dimensional structure. Recreating this feature on synthetic systems would not only allow reproducing biological functions but also developing new functions that align with our technological needs. This has inspired the development of conformationally ordered synthetic oligomers known as foldamers^1^,^2^,^3^,^4^,^5^,^6^,^7^,^8^, which are able to adopt folded conformations through non-covalent intramolecular interactions encoded in their primary structure. In the last years, there have been remarkable advances in the design of foldamers that self-assemble into well-defined structural motifs^1^,^2^,^3^,^4^,^5^,^6^,^7^,^8^, such as helices, pillars and sheets, among others. Many foldamers have been explored in diverse fields including protein mimicking^9^,^10^,^11^,^12^,^13^,^14^,^15^, molecular recognition^16^,^17^,^18^, self-assembly^19^,^20^,^21^, and photoinduced electron and energy transfer^22^,^23^,^24^,^25^, with very promising results.

Charge transport in single molecules is of utmost importance in the development of molecular-scale electronic components. Among these, molecular wires^26^,^27^,^28^,^29^,^30^,^31^ show a prominent position since they allow interconnecting different elements giving rise to complex molecular circuitry and to the transduction of biochemical events. A lot of effort^26^,^27^,^28^,^29^,^30^,^31^ has been devoted to understanding through-bond charge transport across molecular wires with different lengths and conjugation to quantify their charge transport capabilities and to establish the charge transport mechanism. However, in higher-order systems, such as biological systems and organic solids, long-range electron transport does not only depend on the inherent properties of the individual molecules imposed by their chemical structure and *π*-conjugation but also on the spatial organization of such molecules^32^,^33^. In fact, a strong electronic coupling between the frontier orbitals of adjacent molecules, which is often associated with an excellent spatial overlap between their *π*-systems, is necessary to ensure efficient charge transport. This has motivated the study of synthetic molecular wires that impose through-space charge transport schemes either by a combined though-bond/through-space channel^34^,^35^,^36^,^37^,^38^ or by a through-space channel^39^,^40^,^41^,^42^, and also of DNA^43^,^44^,^45^,^46^,^47^,^48^,^49^ in which base pairs are stacked at virtually the graphitic interlayer distance.

Herein, we report a new family of foldamers consisting of an increasing number of anthracene units, which fold by a combination of intramolecular hydrogen bonds and aromatic interactions. Such folding process opens up an efficient through-space charge transport channel across the interacting anthracene moieties (Fig. 1). As a matter of fact, single-molecule conductance measurements carried out on this series of foldamers, using the scanning tunnelling microscopy-based break junction (STM-BJ) technique^50^,^51^, reveal exceptionally high conductance values (*G*) in the order of 10^−1^ *G*_0_ and a low length decay constant (*ß*) of 0.02 Å^−1^. These values exceed the *G* and *ß* values observed in molecular junctions that make use of through-space charge transport pathways^34^,^37^,^42^ (*G*=10^−4^ to 10^−2^ *G*_0_, *ß=*0.21 to 1.16 Å^−1^), including DNA^43^,^44^,^48^ (*G*=10^−4^ to 10^−2^ *G*_0_, *ß*=0.18 to 0.43 Å^−1^).

## Results

### Design and synthesis

As our foldamers, we have designed and synthesized a series of oligomeric structures that combine 9,10-dimethyleneanthracene units stitched together by 2,6-pyridinedicarboxamides (Fig. 2). The series shows an increasing number of anthracene moieties: dimer-NH_2_; trimer-NH_2_; tetramer-NH_2_; and pentamer-NH_2_. Anthracenes have been selected among other acenes because of their well-balanced properties in terms of charge transport, stability and solubility^52^. The presence of the 2,6-pyridinedicarboxamides^53^ promotes a sigmoidal conformation because of the favourable intramolecular hydrogen-bonding interactions. This folded conformation is reinforced by aromatic interactions between the anthracene units, which are also favoured by the optimal parallel arrangement and the flexibility provided by the methylene groups that connect the amide and the anthracene moieties. Triisopropylsilyl (TIPS) groups have been introduced in the pyridine units to ensure the solubility of the oligomers. Terminal amine groups have been selected as they have proven to be effective anchoring groups for attaining single-molecule junctions^54^.

The oligomers were first synthesized as tert-butoxycarbonyl-protected amines (-NHBoc), which were deprotected afterwards in acidic media to make available the terminal amines (-NH_2_) as anchoring groups. The structure of both protected and deprotected foldamer series has been confirmed by nuclear magnetic resonance (NMR) and matrix-assisted laser desorption ionization (coupled to a time-of-flight (TOF) analyser) mass spectrometry. All details of the synthesis and characterization of the oligomers are given in the supporting information (Supplementary Note 1).

### Determination of the folded structure

A combination of X-ray crystallography, NMR, ultraviolet–visible absorption and photoluminescence (PL) spectroscopy, infrared ion dip spectroscopy and theoretical calculations provide strong evidence that the oligomers adopt a folded structure.

Crystals suitable for X-ray crystallography could be obtained for trimer-NHBoc from dimethylsulfoxide (DMSO) solutions. The X-ray diffraction analysis confirms a sigmoidal and folded structure of trimer-NHBoc and also provides an insight into the interactions between the anthracene moieties in the solid state (Fig. 3a). Remarkably, even considering the interference of DMSO with the folding process—the X-ray structure reveals the presence of two DMSO molecules bound to the 2,6-pyridinedicarboxamides by hydrogen bonds—the high predisposition of the anthracene moieties to interact between each other is evidenced. In fact, the central anthracene adopts a CH–*π* conformation by flipping over the DMSO molecules to stick to the two external anthracene moieties.

To study the folding process in solution, we carry out all the experiments with the NHBoc foldamer series as it represents a closer model to the break-junction scenario, in which the free amines are anchored to the gold tips. 1,1,2,2-Tetrachloroethane (TCE) is selected as a solvent to carry out the studies because the oligomers are highly soluble in it; it has a high boiling point (146 °C) optimal for the break-junction studies; and it has a low hydrogen bond basicity (Σ*β*^H^=0.08)^55^, which favours the formation of intramolecular hydrogen bonds that hold together the 2,6-pyridinedicarboxamide backbone in a sigmoidal configuration. The NMR anthracene aromatic signals of dimer-NHBoc and trimer-NHBoc in TCE-*d*_2_ at 25 °C appear shifted upfield and broadened in comparison to the Boc-protected 9,10-bis(aminomethyl)anthracene (A-NHBoc) that is used as a reference (Fig. 3b,c). This is consistent with the close proximity between anthracene moieties, which results in the observed shielded signals as an effect of anisotropy. Moreover, a more substantial broadening is clearly observed in the NMR spectra of tetramer-NHBoc and pentamer-NHBoc that leads to an almost complete disappearance of the aromatic signals. We first ascribed this effect to the aggregation of the tetramer-NHBoc and pentamer-NHBoc in solution at NMR concentrations (10^−3^ to 10^−4^ M). However, this contradicts the high solubility of the whole NHBoc foldamer series in TCE. Two-dimensional diffusion-ordered spectroscopy (DOSY) NMR studies at 25 °C reveal that the broadening of the aromatic signals is indeed not related to aggregation in solution, as the signals of the oligomers diffuse at the same rate, which means that the oligomers are monodisperse species in solution (Fig. 3d). Therefore, the complex NMR observed for tetramer-NHBoc and pentamer-NHBoc indicates the coexistence of multiple conformers at room temperature in a complex dynamic equilibrium. This is supported by variable temperature NMR (VT-NMR) experiments carried out from –40 to 140 °C that show how the complex fine structure of the pentamer-NHBoc coalesces at increasing temperatures into a simpler NMR spectrum that can be easily assigned to the structure (Fig. 3e), as the high temperatures disrupt the intramolecular interactions, unfolding the oligomers.

The ultraviolet–visible electronic spectra in TCE showed the typical features of anthracene derivatives with four vibronic bands on the main electronic absorption (Fig. 3f). An electronic interaction between anthracene moieties is clearly observed, as the absorption bands appear increasingly bathochromically shifted with an increasing number of anthracene units in the oligomers. When dimer-NHBoc was compared with reference A-NHBoc, a bathochromic shift (Δ*λ*) of 1.0 nm was observed. The electronic interactions between anthracenes become more evident when increasing the number of anthracenes in the oligomers as the bathochromic shifts become increasingly larger in trimer-NHBoc (Δ*λ*=1.5 nm), tetramer-NHBoc (Δ*λ*=2.0 nm) and pentamer-NHBoc (Δ*λ*=5.5 nm), when compared with A-NHBoc, respectively. Almost invariable changes were observed in the absorption spectra on dilution (from 10^−4^ to 10^−6^ M), which is consistent with the intramolecular nature of the interactions between anthracene units.

The intramolecular aromatic interactions between anthracenes are also evidenced in the ultraviolet–visible PL studies. The PL spectra of the -NHBoc foldamer series show the typical emission features of anthracene derivatives with four vibronic bands (Fig. 3g). Overall, the observed PL bands are bathochromically shifted as the number of anthracene moieties in the oligomers increases, in line with the absorption measurements. When dimer-NHBoc was compared with A-NHBoc, using the 0-1 transition as a reference, a bathochromic shift (Δ*λ*=1.0 nm) is clearly observed. The bathochromic shifts become increasingly larger in trimer-NHBoc (Δ*λ*=1.5 nm), tetramer-NHBoc (Δ*λ*=2 nm) and pentamer-NHBoc (Δ*λ*=6.0 nm). These energy changes are coupled to a change on the relative intensity of the 0-2 and 0-3 transitions that increase together with the number of anthracene moieties in the oligomer. Such increase of the relative intensity is due to an underlying transition that corresponds to the anthracene excimer (∼500 nm)^56^. The presence of the anthracene excimer in the PL spectra evidences the face-to-face overlap between anthracenes in solution. Most importantly, almost invariable changes were observed in the PL spectra on dilution (from 10^−5^ to 10^−7^ M), which also supports the intramolecular nature of the interactions between anthracenes.

To obtain a much clearer view of the folding process, conformation-specific vibrational spectroscopic studies in the gas phase were carried out as they allow elucidating the intrinsic structures of moderately large molecules (typically <500–600 Da) without the interferences of packing or solvation. Remarkably, dimer-NHBoc (984.33 Da) and trimer-NHBoc (1,532.10 Da) could be studied even if they roughly duplicate and triplicate, respectively, the typical molecular weight for this kind of measurements^57^,^58^. The full experimental description is given in Supplementary Information. In brief, an infrared laser pulse (1,064 nm) was used to transfer the intact foldamer molecules from solid state to gas phase. Then, the target vapourized sample was diluted and expanded in an argon jet and skimmed to generate a cold collimated beam. In the extraction and acceleration regions of the linear TOF, an ultraviolet dye laser ionized the foldamers through a resonant two-photon ultraviolet excitation (∼34,000 cm^−1^). Conformation-selective spectra could be obtained from a third tunable optical parametric oscillator/optical parametric amplifier infrared laser through infrared ion dip spectroscopy. The conformationally specific vibrational spectra could be interrogated in the C–H and N–H stretch regions, which are extremely sensitive to the local hydrogen-bonded conformational environment. The infrared experimental spectra of dimer-NHBoc and trimer NH-Boc are very similar (Fig. 3h,i) with a set of bands in the 2,850–3,100 cm^−1^ region, which correspond to the C–H stretch vibrational modes. With regard to N–H modes, the experimental band about 3,430 cm^−1^ corresponds to the symmetric and antisymmetric coupled modes of the amide nitrogens. The presence of a single red-shifted band points out that the amide nitrogens are equivalent and that they are weakly hydrogen bonded to the nitrogen atoms of pyridine rings. A second set of N–H bands should be observed, which would correspond to the carbamate nitrogens. However, these modes are not observed in the experimental spectra. This is not strange since these atoms are not forming hydrogen bonds and hence, these bands should be weaker. The spectra of several minimized conformers of dimer-NHBoc and trimer-NHBoc were calculated and compared with the experimental spectra to assign the intrinsic folded conformations. To ensure that the assignment is correct, the spectra were calculated from the minimized structure dimer-NHBoc and trimer-NHBoc exhibiting TIPS groups. The good agreement between experimental and calculated spectra (Fig. 3h,i) illustrates that the foldamers adopt a *π*–*π* conformation between the anthracene moieties (see below). The simulated spectrum for the alternative CH–*π* conformation for dimer-NHBoc does not fit with the experimental data confirming univocally that the observed conformation is *π*–*π* (Supplementary Fig. 1). In the case of trimer-NHBoc the simulations of the *π*–*π* and the CH–*π* conformers are similar and both could account for the experimental spectrum (Supplementary Fig. 2). However, the higher correlation of the simulated *π*–*π* conformer with the experimental spectrum, the clear observation of the *π*–*π* conformation for dimer-NHBoc and the larger stability found for the *π*–*π* conformers from modelling (see below) indicate a preferential *π*–*π* conformation for trimer-NHBoc, as well.

Overall, from the combination of all different characterization techniques, we can safely conclude that the foldamers adopt a folded conformation in the solid state, in solution and in the gas phase. X-ray crystallography of trimer-NHBoc shows a folded structure in the solid state even in the presence of two crystallization DMSO molecules that impose a CH–*π* conformation (Fig. 3a). NMR, ultraviolet–visible absorption and PL measurements in solution (Fig. 3c–g) show the close proximity of the anthracene moieties (shielding of the NMR anthracene signals) and an intramolecular electronic interaction among them (batochromically shifted absorption and emission features) that gives rise to a complex intramolecular dynamic equilibrium between different *π*–*π* and CH–*π* conformers (a single diffusing DOSY signal for the foldamers and also denaturation of the foldamer at high temperature observed in VT-NMR, with a preferential *π*–*π* conformation as the number of anthracenes increases (anthracene excimer emission evidencing a face-to-face overlap between anthracenes). Conformation-specific vibrational spectroscopic studies confirm a more favourable *π*–*π* conformation for dimer-NHBoc and trimer-NHBoc in the gas phase (Fig. 3h,i).

Theoretical calculations were carried out to provide a detailed picture of the fundamental interactions determining the energetics and structure of the folded molecules (Fig. 4a). The TIPS groups have a marginal effect on the geometrical and electronic structure and were omitted. The effect of the (implicit) solvent was modelled and was found to have a negligible effect on the geometries of dimer-NH_2_ and therefore was not used in the geometry optimizations. On the basis of the spectroscopic and X-ray data described above two different types of folded conformations were explored: CH–*π* and *π*–*π*. On one hand, the CH–*π* conformations were based on the crystal structure of trimer-NHBoc using partial optimizations at the B3LYP/6-31 g(d,p) level in which only the amines and hydrogens were allowed to relax, as full optimizations lead to *π*–*π* structures. Trimer-NHBoc crystallizes in a CH–*π* conformation yet, when the co-crystallizing solvent molecules were removed and the structure was optimized in vacuum, it changed into a *π*–*π* structure, which indicates that interconversion between CH–*π* and *π*–*π* folded structures is energetically feasible. On the other hand, classical molecular dynamics simulations were run followed by optimizations at the BLYP/def2-TZP-D3BJ level to obtain the *π*–*π* conformers studied (see Supplementary Information for full details). Overall, density functional theory molecular mechanics and semi-empirical models are found to favour compact *π*–*π* conformations, in line with our experimental observations. The first CH–*π* stabilized structure of dimer-NH_2_, which also shows the highest CH–*π* character of the whole foldamer series, is predicted to be 2.1 kcal mol^−1^ above the lowest lying *π*–*π* conformer. The distance between stacked anthracenes for the *π*–*π* dimer-NH_2_ is 3.26 Å. The structures of trimer-NH_2_, tetramer-NH_2_ and pentamer-NH_2_ are substantially more complex. As a matter of fact, the computed structures confirm that the foldamers are held together by a continuous interplay of aromatic interactions between anthracene units, consistent with the VT-NMR and DOSY experiments that confirm a complex conformational equilibrium.

We computed the electronic structure of CH–*π* and *π*–*π* conformations at the B3LYP/6-311+g(2d,p) level surrounded by implicit solvent (Fig. 4b). The electronic structure of the frontier orbitals for the four foldamers is qualitatively similar for the *π*–*π* and the CH–*π* series. The HOMO orbitals for all foldamers have consistently large densities on anthracene moieties, while the LUMO orbitals have often, but not exclusively, larger densities on the pyridine-2,6-dicarboxamide residues (Fig. 4b and Supplementary Figs 3–6). In addition, in all studied cases, the terminal amines are electronically coupled to the terminal anthracenes in the HOMO orbitals. In the case of the CH–*π* conformers, the anthracene units are electronically decoupled at the HOMO level. In contrast, the *π*–*π* conformers, due to the stacking conformations, show HOMO orbitals with densities that spread over more than one anthracene moiety. Furthermore, in all foldamers, the CH–*π* and *π*–*π* conformations show nearly degenerate occupied orbitals for each anthracene unit with energies from −5.3 to −5.6 eV (Supplementary Table 1). In particular, the HOMO degeneracy of the *π*–*π* conformers extends to the HOMO–1 (dimer-NH_2_, trimer-NH_2_, tetramer-NH_2_ and pentamer-NH_2_), HOMO–2 (trimer-NH_2_, tetramer-NH_2_ and pentamer-NH_2_), HOMO–3 (tetramer-NH_2_ and pentamer-NH_2_) and HOMO–4 (pentamer-NH_2_ only), and in most cases with densities that spread over more than one anthracene moiety (Supplementary Figs 5–7). All foldamers also share very similar ionization potentials ranging from –5.3 to –5.4 eV. A comparison of the HOMO and HOMO–1 for the CH–*π* and *π*–*π* conformers in the simplest foldamer, dimer-NH_2_, illustrates the differences between conformers (Supplementary Fig. 7). For the *π*–*π* conformer, there is an electronic density path linking the terminal amines and both anthracene moieties in the HOMO and HOMO–1. Furthermore, for this conformer both molecular orbitals present very similar electron densities and energies (110 meV). In contrast, for the CH–*π* conformer, the HOMO and the HOMO–1 orbitals show localized electron densities on different anthracenes and a slightly larger energy difference (190 meV). Overall, the *π*–*π* and CH–*π* conformers for the whole foldamer series exhibit degenerate occupied frontier orbitals with large electron density on the anthracene moieties and with ionization potentials which are closely aligned to the Fermi energy of gold (−5.5 eV). This provides a viable pathway for charge transport across the molecule through the anthracene moieties.

### Single-molecule charge transport studies

**Figure 1:**
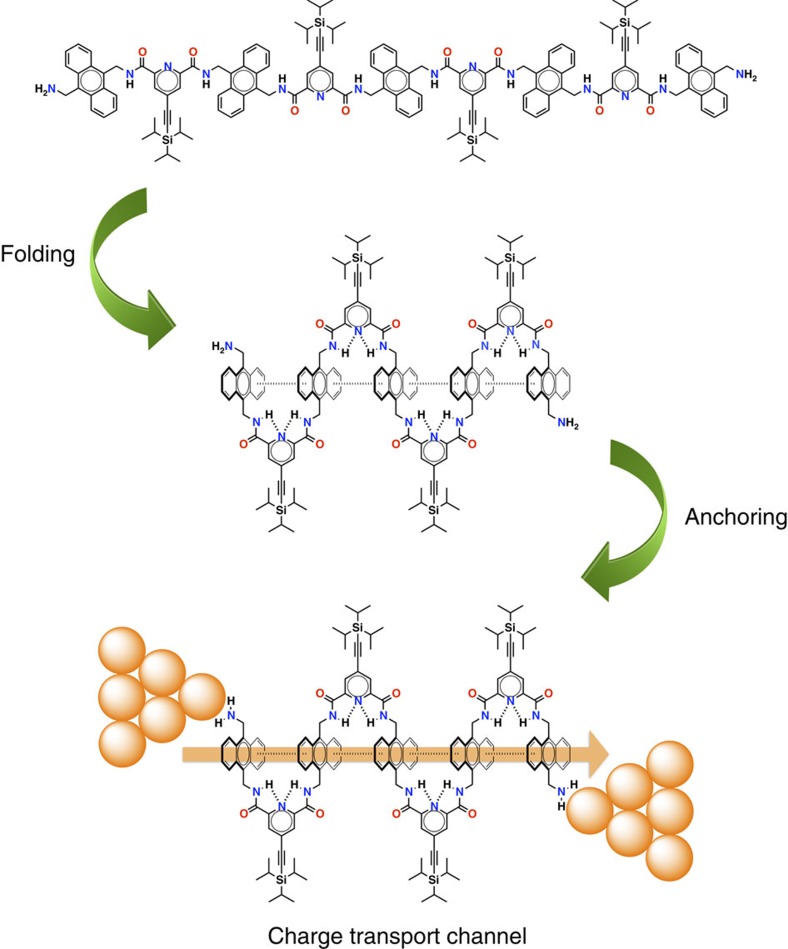
*π*-Folded molecular junctions. Schematic representation of the folding and anchoring processes needed to obtain *π*-folded molecular junctions from a representative member of the foldamer family studied in this work.

**Figure 2:**
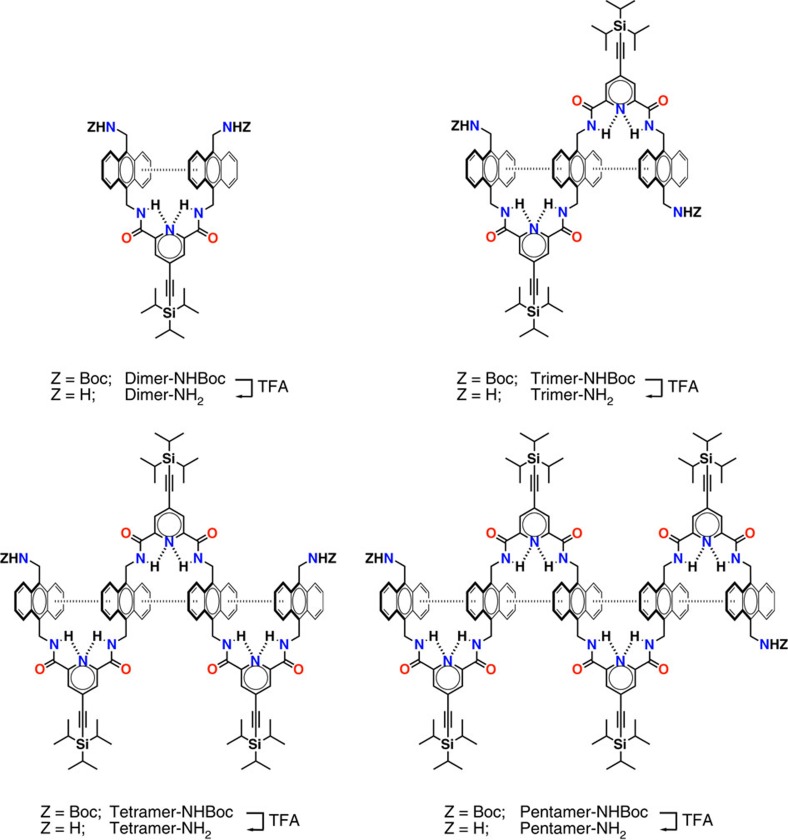
Chemical structures of the foldamers. Structures of the complete -NHBoc and -NH_2_ foldamer series studied in this work: dimer-NHBoc, dimer-NH_2_, trimer-NHBoc, trimer-NH_2_, tetramer-NHBoc, tetramer-NH_2_, pentamer-NHBoc, and pentamer-NH_2_. Arrows indicate the transformation of the -NHBoc series into the -NH_2_ series.

**Figure 3:**
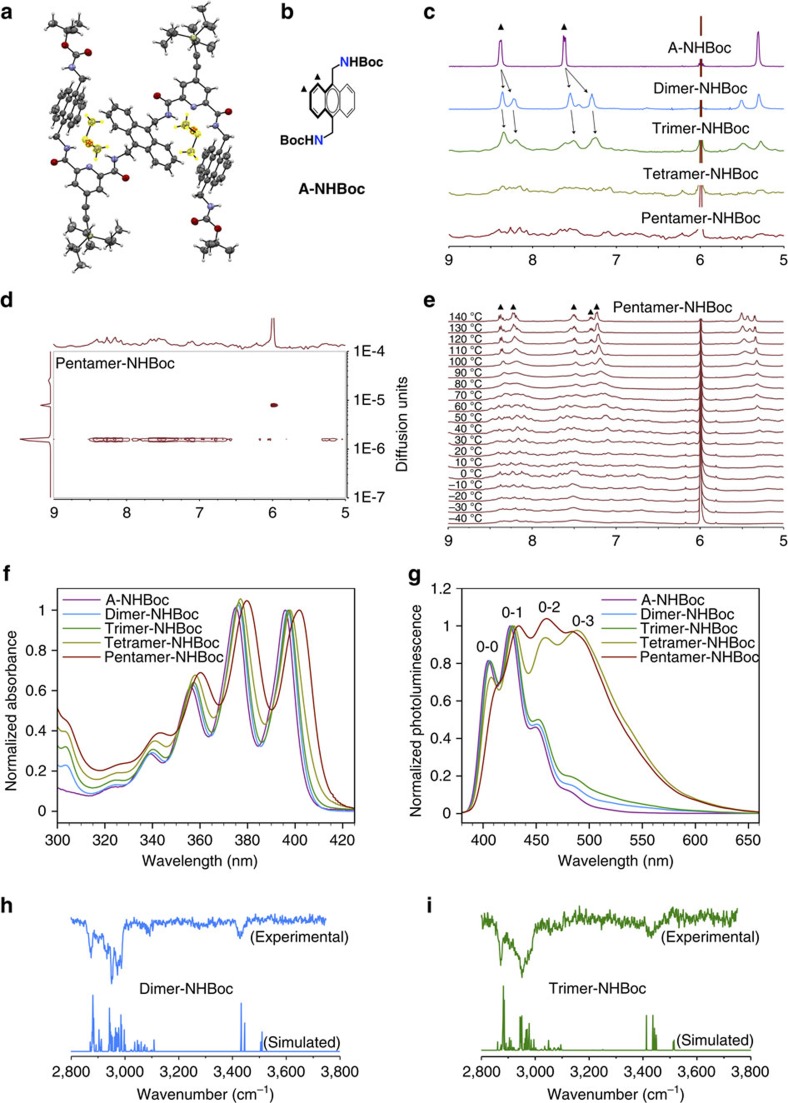
Structural characterization of the foldamers. (**a**) Solid-state structure of trimer-NHBoc with two DMSO molecules (highlighted in yellow). (**b**) Chemical structure of A-NHBoc. (**c**) NMR spectra of the foldamer NHBoc series at 25 °C. (**d**) Two-dimensional DOSY NMR of pentamer-NHBoc at 25 °C. (**e**) VT-NMR of pentamer-NHBoc. (**f**) Absorption and (**g**) PL spectra of the NHBoc foldamer series at 25 °C. Infrared ion dip spectrum of (**h**) dimer-NHBoc and (**i**) trimer-NHBoc. The triangles in **b**–**e** indicate the signals that correspond to anthracene protons.

**Figure 4:**
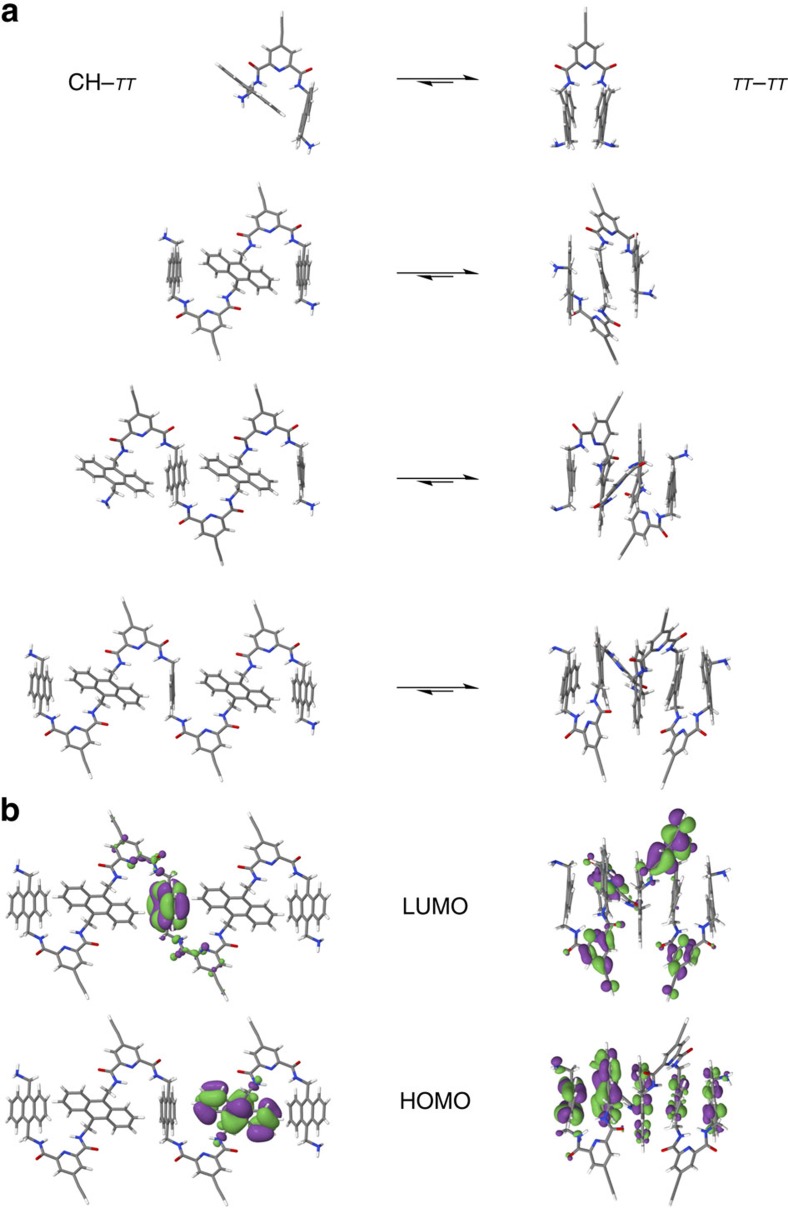
Computed structure of the foldamers. (**a**) Representative CH–*π* and *π*–*π* structures for the different foldamers. (**b**) HOMO and LUMO orbitals for CH–*π* and *π*–*π* conformers of pentamer-NH_2_.

**Figure 5:**
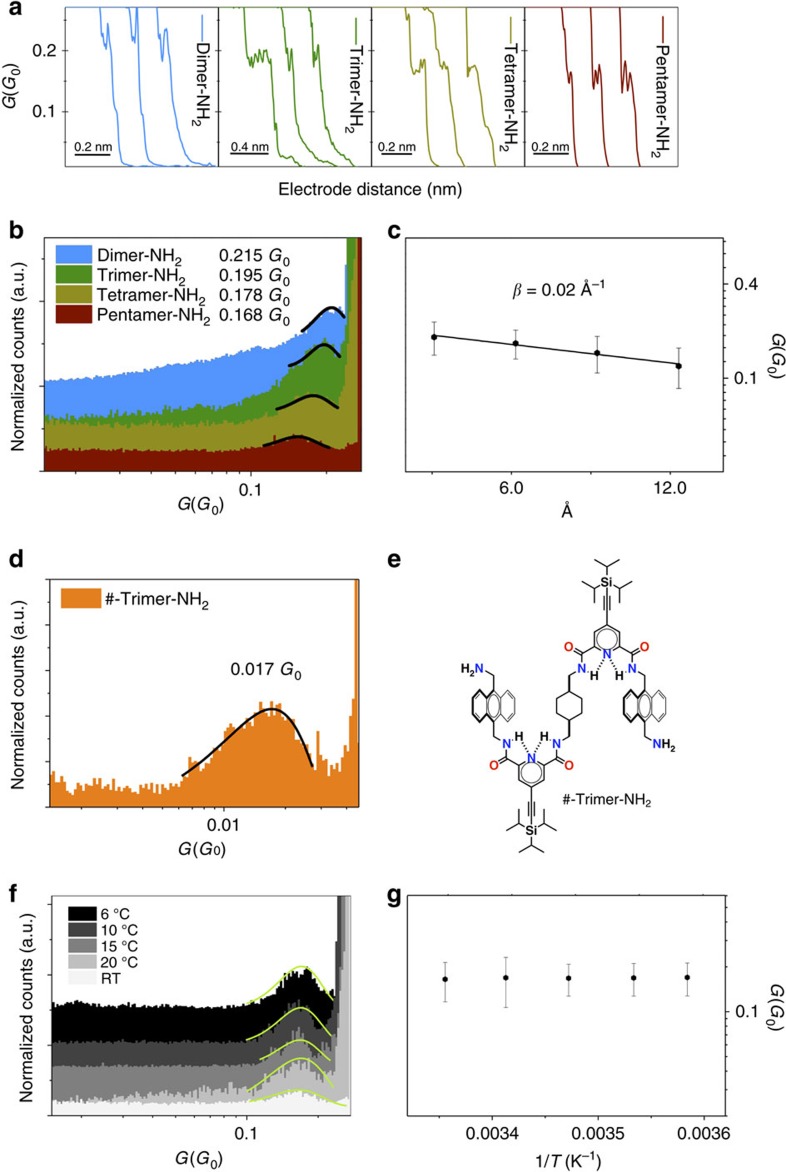
Single-molecule conductance measurements. (**a**) Individual pulling traces and (**b**) conductance histograms of the different foldamers. All conductance values were extracted from Gaussian fits. (**c**) Calculated *ß* value for the *π*–*π* foldamers. Error bars capture the variability in peak position. (**d**) Conductance histograms for #-trimer-NH_2_. (**e**) Structure of #-trimer-NH_2_. (**f**) Conductance histograms for the pentamer-NH_2_ at variable temperatures. (**g**) Arrhenius plot of the single-molecule conductance for the pentamer-NH_2_. Error bars represent the s.d. of the experimental conductance values extracted from the full width at half maximum of the Gaussian fits. Applied voltage bias were 5 mV for the trimer-NH_2_–pentamer-NH_2_ series and 50 mV for dimer-NH_2_.

Single-molecule charge transport characterization of each oligomer was carried out in single-molecule junctions using the STM-BJ technique^50^,^51^. The experiments were conducted in TCE at very low concentrations (<10^−9^ M) to ensure an efficient anchoring of individual molecules between the electrodes and to avoid any aggregation in solution. Briefly, the STM-BJ is based on driving a Au STM tip in and out of contact to/from a Au(111) substrate functionalized with the target foldamer. During the contact process, individual oligomers can spontaneously bridge between both biased (few mV) electrodes via the two -NH_2_ terminal groups (Fig. 1, bottom). The current is then recorded for each pulling stage in the form of current versus time/displacement (Fig. 5a), and the traces displaying molecular plateau features below the quantum conductance (*G*_0_=2*e*^2^*h*^−1^) were used to build the conductance histograms (Fig. 5b). The observed maxima in the histograms represent the most probable conductance values for the formed single-molecule contact (see Supplementary Information for details). The most intense (higher counting) conductance peak, which corresponds to the most probable molecular junction configuration, shows very large conductance values in the range of 10^−1^ *G*_0_ for all foldamers: 0.215 *G*_0_ for dimer-NH_2_; 0.195 *G*_0_ for trimer-NH_2_; 0.178 *G*_0_ for tetramer-NH_2_; and 0.168 *G*_0_ for pentamer-NH_2_. Such high conductance values decrease exponentially with an increasing number of anthracenes in the molecular backbone. By fitting the conductance data to a simple tunnelling transport regime, *G*=*Ae*^*−ßL*^, and using the distance between *π*-stacked anthracenes estimated from the simulations (3.26 Å), a very low length decay constant *ß*=0.02 Å^−1^ is obtained (Fig. 4c).

To experimentally confirm that charge transport takes place preferentially through the interacting anthracene moieties, a truncated trimer (#-trimer-NH_2_) was synthesized, in which the central anthracene ring has been substituted by a non-aromatic cyclohexane ring (Fig. 5d,e). Conductance histograms for #-trimer-NH_2_ showed that the highest conductance peak (0.017 *G*_0_) is one order of magnitude smaller than the corresponding conductance observed for the homologous trimer-NH_2_ (0.195 *G*_0_). This substantial conductance drop can be ascribed to the interrupted conduction channel because of the lack of an anthracene moiety in #-trimer-NH_2_ that interferes with charge transport. Consequently, the high conductance values observed are undoubtedly owed to the presence of a charge transport channel across the interacting anthracene units.

To validate the above proposed tunnelling charge transport mechanism in the studied single-molecule junctions, we have measured temperature-dependent single-molecule conductance for the longer pentamer-NH_2_ moiety (Fig. 5f,g). To avoid any possible unfolding, we covered a temperature range below room temperature from 6 to 25 °C. The invariance of the single-molecule conductance versus temperature around the working room temperature for the longer backbone (Fig. 5g) supports a tunnelling transport regime throughout all the conductance series in Fig. 5b,d. It is worth noticing in Fig. 5g that while the conductance dispersion (represented as error bars) is fairly similar within the studied temperature range, the single-molecule experimental yield (defined as the percentage of current traces displaying single-molecule events or plateaus over the total collected traces) drops down by a factor of four from the lowest to the highest working temperature, which results in the observed counts decrease in Fig. 5f histogram peaks from top to bottom. This behaviour has been observed before in single-molecule transport studies^59^ and is ascribed to thermal instabilities at the molecule/electrode contact as the temperature is raised.

Overall, the conductance measurements carried out point towards a coherent tunnelling charge transport process across the anthracene units as supported by the presence of a charge transport channel across the interacting anthracene units—this is consistent with the calculated electronic structure and evidenced by the conductance drop observed when comparing trimer-NH_2_ and #-trimer-NH_2_ (Fig. 5b,d)—and by the invariance of the single-molecule conductance versus temperature (Fig. 5g), which supports a tunnelling transport regime.

The molecule–electrodes electronic coupling strength (Γ) has been estimated from a single-channel resonant tunnelling model^60^,^61^ yielding values between 50 and 60 meV from pentamer-NH_2_ to dimer-NH_2_ (Supplementary Note 2). Even if such values support the observed coherent tunnelling behaviour^60^, they cannot be used to confirm whether charge transport is resonant, but nevertheless, they point out to a high coupling scenario^62^.

## Discussion

We have reported a series of synthetic oligomers consisting of an increasing number of anthracene moieties that fold by a combination of hydrogen bonds and aromatic interactions as demonstrated by a series of studies in the solid, liquid and gas phases, and by theoretical calculations. Remarkably, such folding process opens up an efficient through-space charge transport channel across the interacting anthracene moieties that has been confirmed both theoretically and experimentally. First, the computed electronic structure of the frontier orbitals of the foldamer series illustrates the degeneracy of anthracene occupied frontier orbitals, which provides a viable pathway for charge transport across the anthracene moieties. Second, charge transport studies at the single-molecule level show exceptionally high conductance values in the range of 10^−1^ *G*_0_ for all foldamers and a low length decay constant of 0.02 Å^−1^. Third, comparative conductance studies carried out with #-trimer-NH_2_, in which the central anthracene ring has been substituted by a non-aromatic cyclohexane ring, reveal conductance values one order of magnitude smaller than the homologous trimer-NH_2_, confirming that charge transport takes place preferentially through the channel constituted by the interacting anthracene moieties. While a complete picture of the transport mechanism at play is still to be determined, the invariance of the single-molecule conductance versus temperature supports a tunnelling transport regime. Overall, this work illustrates that carefully designed foldamers provide a new and efficient means for charge transport at the single-molecule level and also new application perspectives for foldamers.

## Methods

### Synthesis and structural characterization

All commercially available reagents and solvents were used without further purification. Anhydrous tetrahydrofuran (THF) and toluene were dried through a SPS solvent purification system. All reactions of the compounds were carried out under a nitrogen atmosphere and oven-dried glassware. Column chromatography was carried out using Silica gel 60 (40–60 μm; 230–400 mesh) from VWR. Thin layer chromatography was performed to follow the reaction process by using sheets (20 × 20) of aluminium pre-coated with Silica gel 60 F_254_ from Merck. Ultraviolet-active compounds were detected with an ultraviolet lamp from CAMAG at wavelength *λ*=254 or 366 nm. ^1^H-NMR and ^13^C-NMR spectra were recorded on Bruker Avance 400 or 500 spectrometer at 298 K using partially deuterated solvents as internal references. Chemical shifts are reported in (*δ*) parts per million and referred to the residual solvent peak. DOSY and VT-NMR measurements were performed at the NMR General Services of the University of the Basque Country by Dr José Ignacio Miranda. Matrix-assisted laser desorption ionization (coupled to a TOF analyser) experiments were recorded on Bruker REFLEX spectrometer at POLYMAT by Dr Antonio Veloso. X-ray diffraction measurements were performed at the X-ray diffraction unit of the General Services of the University of the Basque Country by Dr Leire San Felices. Intensity data were collected on an Agilent Technologies Super-Nova diffractometer, which was equipped with monochromated Cu kα radiation (*λ*=1.54184 Å) and Atlas charge-coupled device detector. Measurement was carried out at 150.00(10) K with the help of an Oxford Cryostream 700 PLUS temperature device. Data frames were processed (united cell determination, analytical absorption correction with face indexing, intensity data integration and correction for Lorentz and polarization effects) using the Crysalis software package. The structure was solved using Olex2 and refined by full-matrix least-squares with SHELXL-97. Final geometrical calculations were carried out with Mercury and PLATON as integrated in WinGX.

### Foldamer handling

The -NHBoc and -NH_2_ foldamer series are sensitive to oxygen and light and decompose in solution to the corresponding anthracene endoperoxides in about 24 h. All anthracene-containing compounds were characterized from fresh samples in a light-free environment. In the case the experiments required more than 2 h they were carried out in a nitrogen- and light-free environment (either under an nitrogen stream, using airtight glassware or handled in a glovebox).

### Optical characterization

Absorption and PL spectra were recorded on a Perkin-Elmer Lambda 950 spectrometer and a LS55 Perkin-Elmer Fluorescence spectrometer, respectively, at 298 K.

### Infrared ion dip spectroscopy

Approximately 40 mg of foldamer (dimer-NHBoc or trimer-NHBoc) were used in the experiments. The sample was mixed with nanotubes and deposited on a graphite cylindrical rod (15 mm long and 4 mm diameter). A stepper motor rotated and translated the sample. So, the infrared pulses (1,064 nm, ∼8 ns) from a Q-switched Nd:YAG laser found fresh sample. A full description of technique is described here^63^. The foldamers exhibit broad absorption electronic spectra due to several chromophore anthracene groups. The infrared spectra were recorded at 34,343 and 34,013 cm^−1^ for dimer-NHBoc and trimer-NHBoc, respectively. Finally, the experimental infrared spectra were compared with theoretical spectra. The frequencies of the NH and CH stretch modes, expressed in wavenumbers, were corrected for anharmonicity using the multipliers, 0.995 and 0.98, respectively.

### Computer modelling

We studied different conformers for the dimer-NH_2_ and trimer-NH_2_ foldamers. The TIPS groups have a marginal effect on the geometric and electronic structure of these systems and were omitted for computational efficiency. To generate the starting structures, we first performed molecular dynamics simulations at 298 K with the molecular mechanics MMFF94 force field as implemented in the software Tinker. Collisions with solvent molecules were considered implicitly through the use of stochastic dynamics with a friction coefficient of 2 ps to improve conformational sampling. Simulations were run for 10 ns and snapshots of the trajectory were selected at 50 ps intervals yielding 200 different conformations, which were optimized at MMFF94 level and at the PM6-D3H4 level^64^. The so-obtained geometries were filtered by an algorithm explicitly considering topological symmetry with a root mean squared deviation cut-off of 0.5 Å (ref. 65). The unique conformations were subsequently minimized at the BLYP/def2-SVP-D3BJ (also used for infrared spectra simulations) and BLYP/def2-TZVP-D3BJ (geometries and total energies) levels^66^ within the RI approximation with the programme Orca 3.03 (ref. 67). The effect of the (implicit) solvent was modelled with the programme Gaussian 09 (ref. 68) and found to have a negligible effect on the geometries of the dimer-NH_2_ and was not used in the geometry optimizations. The aforementioned procedure produces prevalently conformers with *π*–*π* stacked anthracenes. The lowest energy minimum for the dimer-NH_2_ and the minima presenting the largest contact between anthracene moieties in the trimer were selected as representative for the *π*–*π* conformations. The pentamer-NH_2_ *π*–*π* conformation was produced by fusing two trimer-NH_2_ molecules followed by optimization, while the *π*–*π* tetramer-NH_2_ was obtained by cutting out one monomer from the pentamer-NH_2_ followed by optimization. CH–*π* conformers were derived from the trimer-NHBoc crystalline structure by, first, substituting the NHBoc groups by amine groups and, second, using partial optimizations at the B3LYP/6-31g(d,p) levels where only the amines and hydrogen atoms were allowed to relax. This was necessary as full optimizations favour *π*–*π* stacked structures. The CH–*π* pentamer-NH_2_ was built by fusing two CH–*π* trimer-NH_2_ molecules, but in this case only the central CH_2_–antracene–CH_2_ unit was optimized. The CH–*π* tetramer-NH_2_ was obtained cutting out one monomer from the pentamer. The electronic structure for neutral and charged foldamers was computed at the B3LYP/6-311+g(2d,p) level, as with this Hamiltonian HOMO and LUMOs eigenvalues are similar to the adiabatic ionization potential and electron affinity of conjugated molecules in low-polarity solvents^69^. The adiabatic ionization potential and electron affinity were explicitly computed for all foldamers and match the HOMO and LUMOs eigenvalues for these systems.

### STM break junction

Foldamer-NH_2_ TFA salt was dissolved in MeOH (saturated) and the free amine was precipitated by addition of triethylamine (TEA), the solid was collected by centrifugation and was washed with MeOH/TEA 20:1 and twice with MeOH and finally vacuum-dried until constant weight. All the conductance measurements were carried out in the dark in anaerobic conditions with a mechanically and electronically isolated PicoSPM I microscope head controlled by a Picoscan 2500 electronics (all from Agilent) and using a homemade PTFE STM cell. Data captures were acquired using a NI-DAQ_mx_/BNC-2110 National Instruments (LabVIEW data acquisition System) and analysed with LabVIEW code. The procedure of a typical break-junction experiment is based on bringing the STM tip to tunnelling distance over a flat clean Au (111) surface area as a first step. The STM feedback is then turned off and the tip is driven into and out of contact with the substrate at a speed of 1–2 V s^−1^. These two-point feedback loop is used to collect thousands of current decays (5,000–6,000). Single-molecule conductance (*G*) was determined using the expression *G*=*I*_step_/*U*_BIAS_, where *I* is the current and *U* is the voltage difference between the two junction electrodes. The current decays are accumulated to semi-logarithmic conductance histograms. The observed plateaus in the individual current decays result in the observed peaks in the conductance histograms and provide an averaged value of the single-molecule conductance. Transient curves that are either noisy or that showed smooth exponential decay because of the absence of molecular bridge formation were rejected when building the histograms using an automatic selection procedure driven by a code written in LabVIEW. The histograms were compiled by applying the same automated selection criteria to each set of the recorded decay curves. The selection procedure allows current traces showing counts exceeding a defined threshold to be added to the conductance histogram. The percentage decay curves that showed clear molecular steps were typically 8–15% and were all selected for building the histograms. This selection process made peaks in the conductance histograms more prominent above the tunnelling background and also allowed a quantitative measure of the yield of molecular junction formation.

### Data availability

Full details on the synthesis and characterization of the foldamers and additional details regarding infrared ion dip spectroscopy, computer modelling and STM break junction are given in the Supplementary Information. Crystallographic data for trimer-NHBoc are deposited with the Cambridge Crystallographic Data Centre under reference number CCDC-1542042.

## Additional information

**How to cite this article:** Carini, M. *et al*. High conductance values in π-folded molecular junctions. *Nat. Commun.* **8,** 15195 doi: 10.1038/ncomms15195 (2017).

**Publisher's note:** Springer Nature remains neutral with regard to jurisdictional claims in published maps and institutional affiliations.

## Supplementary Material

Supplementary Information Supplementary Figures, Supplementary Table 1, Supplementary Notes and Supplementary References

## References

[b1] Gellman S. H. Foldamers: a manifesto. Acc. Chem. Res. 31, 173–180 (1998).

[b2] Hill D. J., Mio M. J., Prince R. B., Hughes T. S. & Moore J. S. A field guide to foldamers. Chem. Rev. 101, 3893–4012 (2001). 11740924 10.1021/cr990120t

[b3] eds Hecht S., Huc I. Foldamers: Structure, Properties, and Applications Wiley (2007).

[b4] Guichard G. & Huc I. Synthetic foldamers. Chem. Commun. 47, 5933–5941 (2011). 10.1039/c1cc11137j 21483969

[b5] Zhang D. W., Zhao X., Hou J. L. & Li Z. T. Aromatic amide foldamers: structures, properties, and functions. Chem. Rev. 112, 5271–5316 (2012). 22871167 10.1021/cr300116k

[b6] Das A. & Ghosh S. Supramolecular assemblies by charge-transfer interactions between donor and acceptor chromophores. Angew. Chem. Int. Ed. 53, 2038–2054 (2014). 10.1002/anie.201307756 24573995

[b7] Hartley C. S. Folding of ortho-phenylenes. Acc. Chem. Res. 49, 646–654 (2016). 26954326 10.1021/acs.accounts.6b00038

[b8] Yu Z. & Hecht S. Remote control over folding by light. Chem. Commun. 52, 6639–6653 (2016). 10.1039/c6cc01423b 27021403

[b9] Porter E. A., Wang X., Lee H.-S., Weisblum B. & Gellman S. H. Antibiotics: non-haemolytic [beta]-amino-acid oligomers. Nature 404, 565–565 (2000). 10766230 10.1038/35007145

[b10] Horne W. S. . Structural and biological mimicry of protein surface recognition by α/β-peptide foldamers. Proc. Natl Acad. Sci. USA 106, 14751–14756 (2009). 19706443 10.1073/pnas.0902663106 PMC2736470

[b11] Lee E. F. . High-resolution structural characterization of a helical α/β-peptide foldamer bound to the anti-apoptotic protein Bcl-xL. Angew. Chem. Int. Ed. 48, 4318–4322 (2009). 10.1002/anie.200805761 PMC2843084 19229915

[b12] Shirude P. S. . Macrocyclic and helical oligoamides as a new class of G-quadruplex ligands. J. Am. Chem. Soc. 129, 11890–11891 (2007). 17845042 10.1021/ja073775h PMC2195897

[b13] De Poli M. . Conformational photoswitching of a synthetic peptide foldamer bound within a phospholipid bilayer. Science 352, 575–580 (2016). 27033546 10.1126/science.aad8352

[b14] Brown R. A., Diemer V., Webb S. J. & Clayden J. End-to-end conformational communication through a synthetic purinergic receptor by ligand-induced helicity switching. Nat. Chem. 5, 853–860 (2013). 24056342 10.1038/nchem.1747

[b15] Laursen J. S., Harris P., Fristrup P. & Olsen C. A. Triangular prism-shaped beta-peptoid helices as unique biomimetic scaffolds. Nat. Commun. 6, 7013 (2015). 25943784 10.1038/ncomms8013 PMC4432622

[b16] Juwarker H., Suk J.-M. & Jeong K.-S. Foldamers with helical cavities for binding complementary guests. Chem. Soc. Rev. 38, 3316–3325 (2009). 20449051 10.1039/b909034g

[b17] Tanatani A., Hughes T. S. & Moore J. S. Foldamers as dynamic receptors: probing the mechanism of molecular association between helical oligomers and rodlike ligands. Angew. Chem. Int. Ed. 41, 325–328 (2002). 10.1002/1521-3773(20020118)41:2<325::aid-anie325>3.0.co;2-1 12491421

[b18] Gan Q. . Helix-rod host-guest complexes with shuttling rates much faster than disassembly. Science 331, 1172–1175 (2011). 21385710 10.1126/science.1200143

[b19] Berl V., Huc I., Khoury R. G., Krische M. J. & Lehn J. M. Interconversion of single and double helices formed from synthetic molecular strands. Nature 407, 720–723 (2000). 11048713 10.1038/35037545

[b20] Kwon S. . Magnetotactic molecular architectures from self-assembly of beta-peptide foldamers. Nat. Commun. 6, 8747 (2015). 26510658 10.1038/ncomms9747 PMC4640081

[b21] Collie G. W. . Shaping quaternary assemblies of water-soluble non-peptide helical foldamers by sequence manipulation. Nat. Chem. 7, 871–878 (2015). 26492006 10.1038/nchem.2353

[b22] Marcos Ramos A. . Supramolecular control over donor-acceptor photoinduced charge separation. J. Am. Chem. Soc. 126, 9630–9644 (2004). 15291567 10.1021/ja0390909

[b23] Zeidan T. A., Wang Q., Fiebig T. & Lewis F. D. Molecular wire behavior in pi-stacked donor-bridge-acceptor tertiary arylureas. J. Am. Chem. Soc. 129, 9848–9849 (2007). 17658811 10.1021/ja073219n

[b24] Wolffs M. . Helical aromatic oligoamide foldamers as organizational scaffolds for photoinduced charge transfer. J. Am. Chem. Soc. 131, 4819–4829 (2009). 19334777 10.1021/ja809367u

[b25] Li X. . Photoinduced electron transfer and hole migration in nanosized helical aromatic oligoamide foldamers. J. Am. Chem. Soc. 138, 13568–13578 (2016). 10.1021/jacs.6b05668 27652807

[b26] Nitzan A. & Ratner M. A. Electron transport in molecular wire junctions. Science 300, 1384–1389 (2003). 12775831 10.1126/science.1081572

[b27] Tao N. J. Electron transport in molecular junctions. Nat. Nanotechnol. 1, 173–181 (2006). 18654182 10.1038/nnano.2006.130

[b28] Genereux J. C. & Barton J. K. Mechanisms for DNA charge transport. Chem. Rev. 110, 1642–1662 (2010). 20214403 10.1021/cr900228f PMC2879062

[b29] Grozema F. C., Siebbeles L. D. A. (eds) Charge and Exciton Transport through Molecular Wires Wiley (2011).

[b30] Aradhya S. V. & Venkataraman L. Single-molecule junctions beyond electronic transport. Nat. Nanotechnol. 8, 399–410 (2013). 23736215 10.1038/nnano.2013.91

[b31] Guldi D. M., Nishihara H. & Venkataraman L. Molecular wires. Chem. Soc. Rev. 44, 842–844 (2015). 25636152 10.1039/c5cs90010g

[b32] Bredas J. L., Beljonne D., Coropceanu V. & Cornil J. Charge-transfer and energy-transfer processes in pi-conjugated oligomers and polymers: a molecular picture. Chem. Rev. 104, 4971–5004 (2004). 15535639 10.1021/cr040084k

[b33] Coropceanu V. . Charge transport in organic semiconductors. Chem. Rev. 107, 926–952 (2007). 17378615 10.1021/cr050140x

[b34] Seferos D. S., Blum A. S., Kushmerick J. G. & Bazan G. C. Single-molecule charge-transport measurements that reveal technique-dependent perturbations. J. Am. Chem. Soc. 128, 11260–11267 (2006). 16925445 10.1021/ja062898j

[b35] Molina-Ontoria A. . [2,2']paracyclophane-based pi-conjugated molecular wires reveal molecular-junction behavior. J. Am. Chem. Soc. 133, 2370–2373 (2011). 21299214 10.1021/ja109745a

[b36] Wielopolski M. . Blending through-space and through-bond pi-pi-coupling in [2,2']-paracyclophane-oligophenylenevinylene molecular wires. J. Am. Chem. Soc. 135, 10372–10381 (2013). 23678866 10.1021/ja401239r

[b37] Chen L. . Multichannel conductance of folded single-molecule wires aided by through-space conjugation. Angew. Chem. Int. Ed. 54, 4231–4235 (2015). 10.1002/anie.201411909 25694026

[b38] Ullmann K. . Single-molecule junctions with epitaxial graphene nanoelectrodes. Nano Lett. 15, 3512–3518 (2015). 25923590 10.1021/acs.nanolett.5b00877

[b39] Kang Y. K., Rubtsov IV, Iovine P. M., Chen J. & Therien M. J. Distance dependence of electron transfer in rigid, cofacially compressed, pi-stacked porphyrin-bridge-quinone systems. J. Am. Chem. Soc. 124, 8275–8279 (2002). 12105906 10.1021/ja012504i

[b40] Vura-Weis J. . Crossover from single-step tunneling to multistep hopping for molecular triplet energy transfer. Science 328, 1547–1550 (2010). 20558715 10.1126/science.1189354

[b41] Batra A. . Quantifying through-space charge transfer dynamics in pi-coupled molecular systems. Nat. Commun. 3, 1086 (2012). 23011140 10.1038/ncomms2083

[b42] Schneebeli S. T. . Single-molecule conductance through multiple pi-pi-stacked benzene rings determined with direct electrode-to-benzene ring connections. J. Am. Chem. Soc. 133, 2136–2139 (2011). 21265533 10.1021/ja111320n

[b43] Guo X., Gorodetsky A. A., Hone J., Barton J. K. & Nuckolls C. Conductivity of a single DNA duplex bridging a carbon nanotube gap. Nat. Nanotechnol. 3, 163–167 (2008). 18654489 10.1038/nnano.2008.4 PMC2747584

[b44] Dulic D. . Direct conductance measurements of short single DNA molecules in dry conditions. Nanotechnology 20, 115502 (2009). 19420440 10.1088/0957-4484/20/11/115502

[b45] van Zalinge H. . Variable-temperature measurements of the single-molecule conductance of double-stranded DNA. Angew. Chem. Int. Ed. 45, 5499–5502 (2006). 10.1002/anie.200601263 16892467

[b46] Slinker J. D., Muren N. B., Renfrew S. E. & Barton J. K. DNA charge transport over 34 nm. Nat. Chem. 3, 228–233 (2011). 21336329 10.1038/nchem.982 PMC3079570

[b47] Livshits G. I. . Long-range charge transport in single G-quadruplex DNA molecules. Nat. Nanotechnol. 9, 1040–1046 (2014). 25344689 10.1038/nnano.2014.246

[b48] Artes J. M., Li Y., Qi J., Anantram M. P. & Hihath J. Conformational gating of DNA conductance. Nat. Commun. 6, 8870 (2015). 26648400 10.1038/ncomms9870 PMC4682165

[b49] Di Ventra M. & Taniguchi M. Decoding DNA. RNA and peptides with quantum tunnelling. Nat. Nanotechnol. 11, 117–126 (2016). 10.1038/nnano.2015.320 26839257

[b50] Xu B. & Tao N. J. Measurement of single-molecule resistance by repeated formation of molecular junctions. Science 301, 1221–1223 (2003). 12947193 10.1126/science.1087481

[b51] Chen F. & Tao N. J. Electron transport in single molecules: from benzene to graphene. Acc. Chem. Res. 42, 429–438 (2009). 19253984 10.1021/ar800199a

[b52] Anthony J. E. Functionalized acenes and heteroacenes for organic electronics. Chem. Rev. 106, 5028–5048 (2006). 17165682 10.1021/cr050966z

[b53] Odriozola I., Kyritsakas N. & Lehn J. M. Structural codons: linearity/helicity interconversion by pyridine/pyrimidine exchange in molecular strands. Chem. Commun. 62–63 (2004). 10.1039/b311045a 14737333

[b54] Venkataraman L., Klare J. E., Nuckolls C., Hybertsen M. S. & Steigerwald M. L. Dependence of single-molecule junction conductance on molecular conformation. Nature 442, 904–907 (2006). 16929295 10.1038/nature05037

[b55] Abraham M. H., Andonian-Haftvan J., Whiting G. S., Leo A. & Taft R. S. Hydrogen bonding. Part 34. The factors that influence the solubility of gases and vapours in water at 298 K, and a new method for its determination. J. Chem. Soc., Perkin Trans. 2, 1777–1791 (1994).

[b56] Sugino M. . Elucidation of anthracene arrangement for excimer emission at ambient conditions. Cryst. Growth Des. 13, 4986–4992 (2013).

[b57] Cocinero E. J., Çarçabal P., Vaden T. D., Simons J. P. & Davis B. G. Sensing the anomeric effect in a solvent-free environment. Nature 469, 76–79 (2011). 21209661 10.1038/nature09693

[b58] León I., Millán J., Cocinero E. J., Lesarri A. & Fernández J. A. Shaping micelles: the interplay between hydrogen bonds and dispersive interactions. Angew. Chem. Int. Ed. 52, 7772–7775 (2013). 10.1002/anie.201303245 23754768

[b59] Hines T. . Transition from tunneling to hopping in single molecular junctions by measuring length and temperature dependence. J. Am. Chem. Soc. 132, 11658–11664 (2010). 20669945 10.1021/ja1040946

[b60] Frisenda R. & van der Zant H. S. J. Transition from strong to weak electronic coupling in a single-molecule junction. Phys. Rev. Lett. 117, 126804 (2016). 27689291 10.1103/PhysRevLett.117.126804

[b61] Komoto Y., Fujii S., Nishino T. & Kiguchi M. High electronic couplings of single mesitylene molecular junctions. Beilstein J. Nanotechnol. 6, 2431–2437 (2015). 26732978 10.3762/bjnano.6.251 PMC4685770

[b62] Matsuhita R., Horikawa M., Naitoh Y., Nakamura H. & Kiguchi M. Conductance and SERS measurement of benzenedithiol molecules bridging between Au electrodes. J. Phys. Chem. C 117, 1791–1795 (2013).

[b63] Rijs A. & Oomens J. in Gas-Phase IR Spectroscopy and Structure of Biological Molecules eds Rijs A., Oomens J. Springer (2015).

[b64] MOPAC2016, version: 16.035L (Stewart Computational Chemistry, Colorado Springs, CO, USA, 2016) Available at http://OpenMOPAC.net.

[b65] O'Boyle N. M. . Open Babel: an open chemical toolbox. J. Cheminformatics 3, 1–14 (2011). 10.1186/1758-2946-3-33 PMC3198950 21982300

[b66] Strutyński K., Melle-Franco M. & Gomes JANF New parameterization scheme of DFT-D for graphitic materials. J. Phys. Chem. A. 117, 2844–2853 (2013). 23473398 10.1021/jp312239n

[b67] Neese F. The ORCA program system. Wiley Interdiscip. Rev. Comput. Mol. Sci. 2, 73–78 (2012).

[b68] Frisch M. J. . Gaussian 09 (Gaussian, Inc., Wallingford, Connecticut, USA, 2009).

[b69] Marco A. B. . Bis(triisopropylsilylethynyl)-substituted pyrene-fused tetraazaheptacene: synthesis and properties. Phys. Chem. Chem. Phys. 18, 11616–11619 (2016). 26910505 10.1039/c5cp07656k

