# Supplementary material for: High conductance values in π-folded molecular junctions
Source: Nat Commun. 2017 May 18;8:15195. doi: 10.1038/ncomms15195 (PMC5454372; doi:10.1038/ncomms15195)
Supplement: Supplementary Information — Supplementary Figures, Supplementary Table 1, Supplementary Notes and Supplementary References [file ncomms15195-s1.pdf]

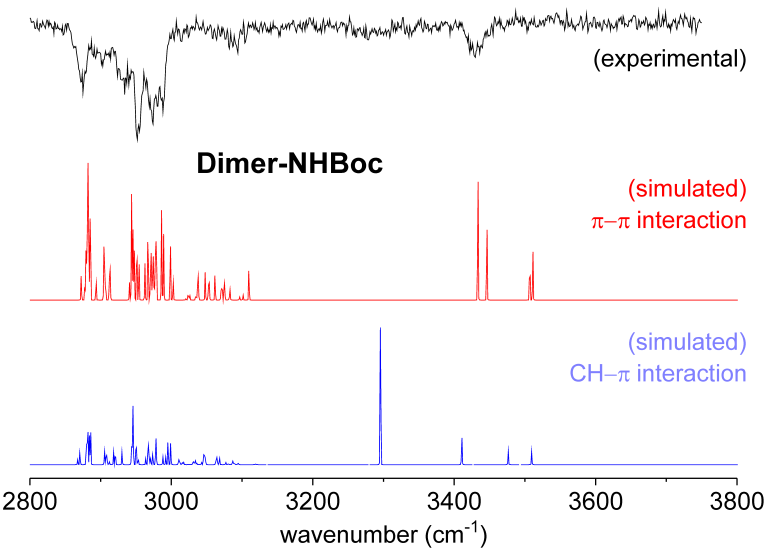

Supplementary Figure 1. IR ion dip spectrum of dimer-NHBoc.

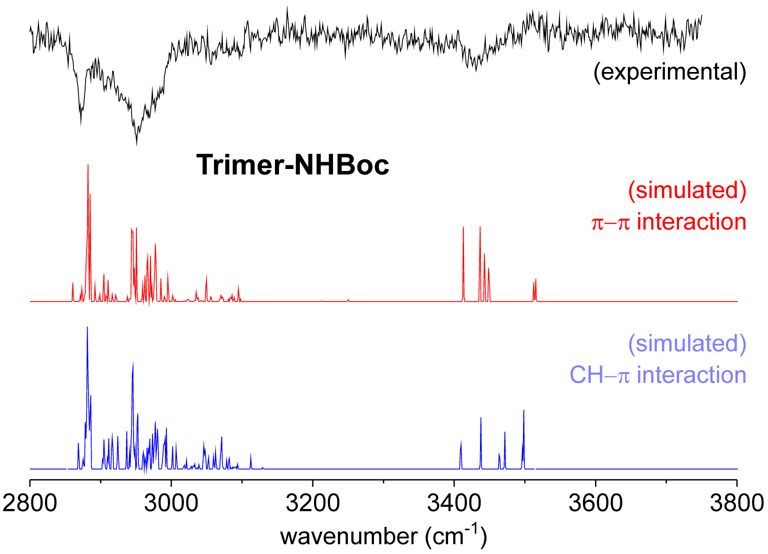

Supplementary Figure 2. IR ion dip spectrum of trimer-NHBoc.

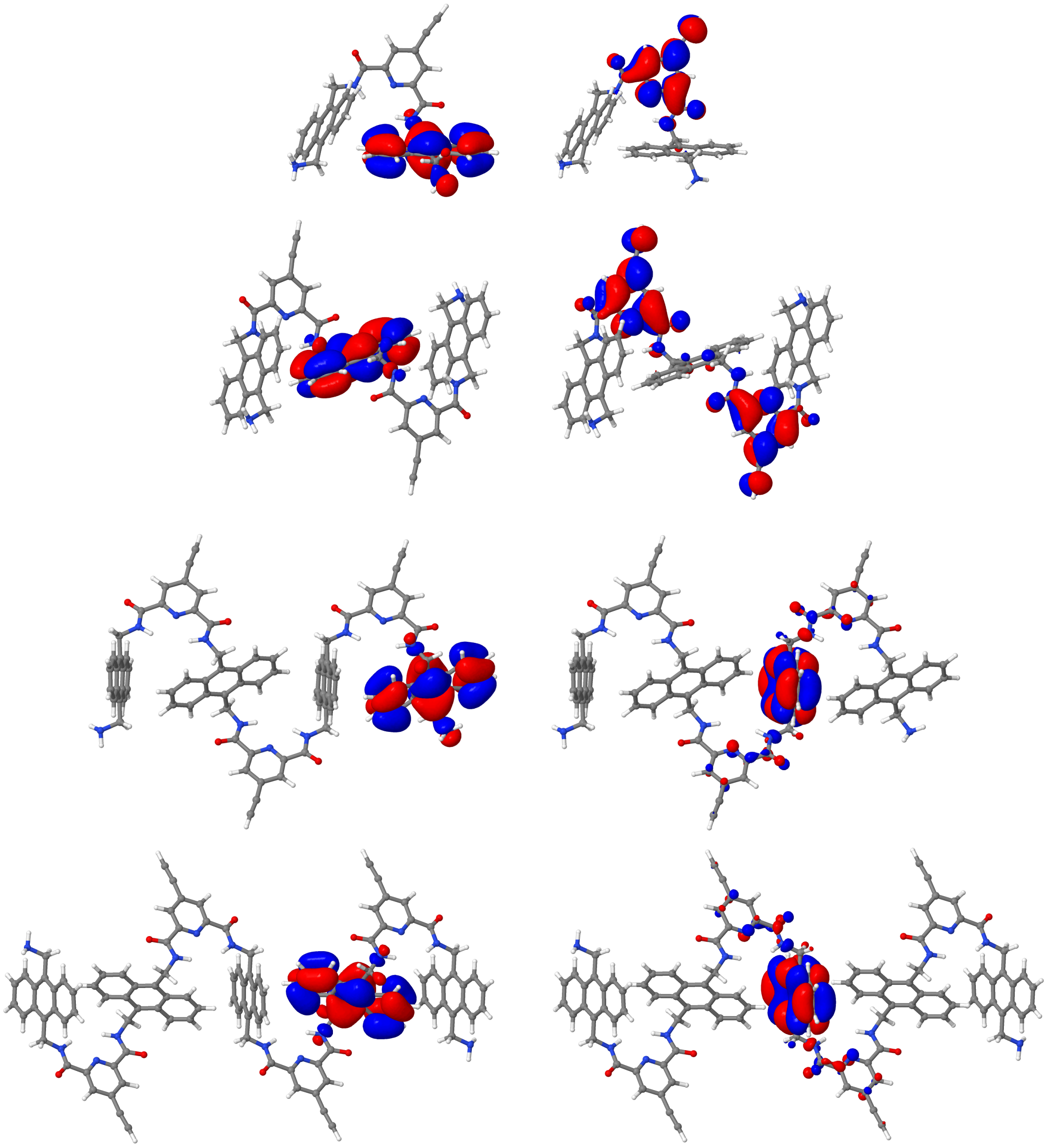

Supplementary Figure 3. HOMO (left) and LUMO (right) of the CH-π folded oligomers.

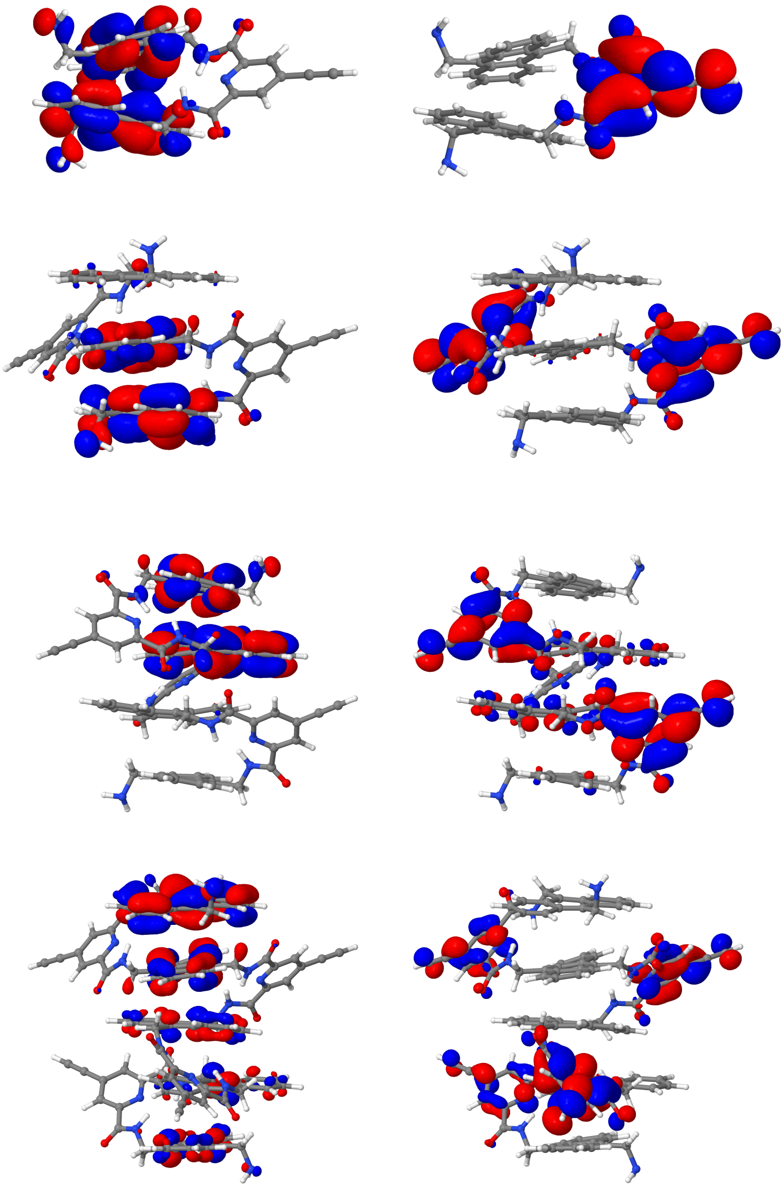

Supplementary Figure 4. HOMO (left) and LUMO (right) of the π-π folded oligomers.

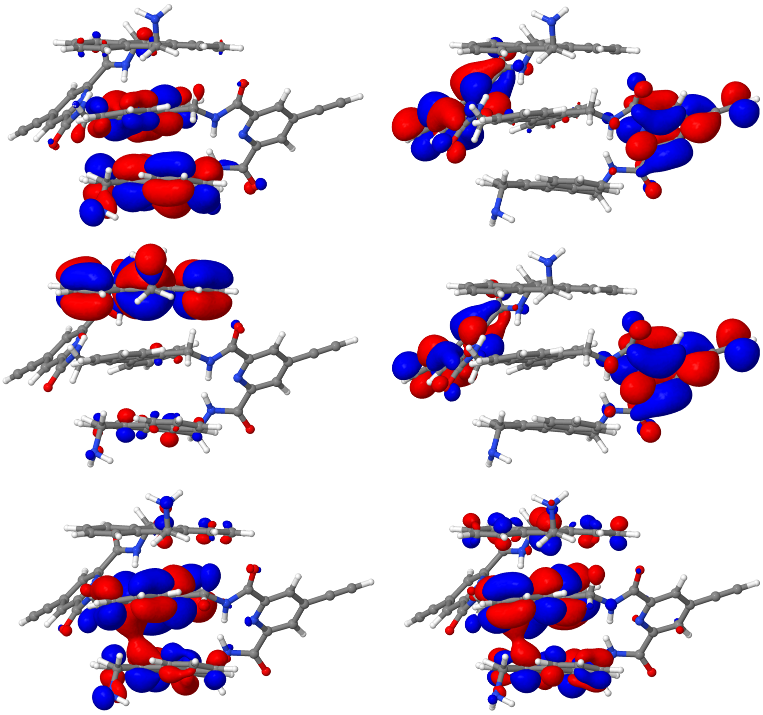

Supplementary Figure 5. Nearly degenerate frontier orbitals of the π-π folded trimer: HOMO and LUMO (top), HOMO-1 and LUMO+1 (middle) and HOMO-2 and LUMO+2 (bottom).

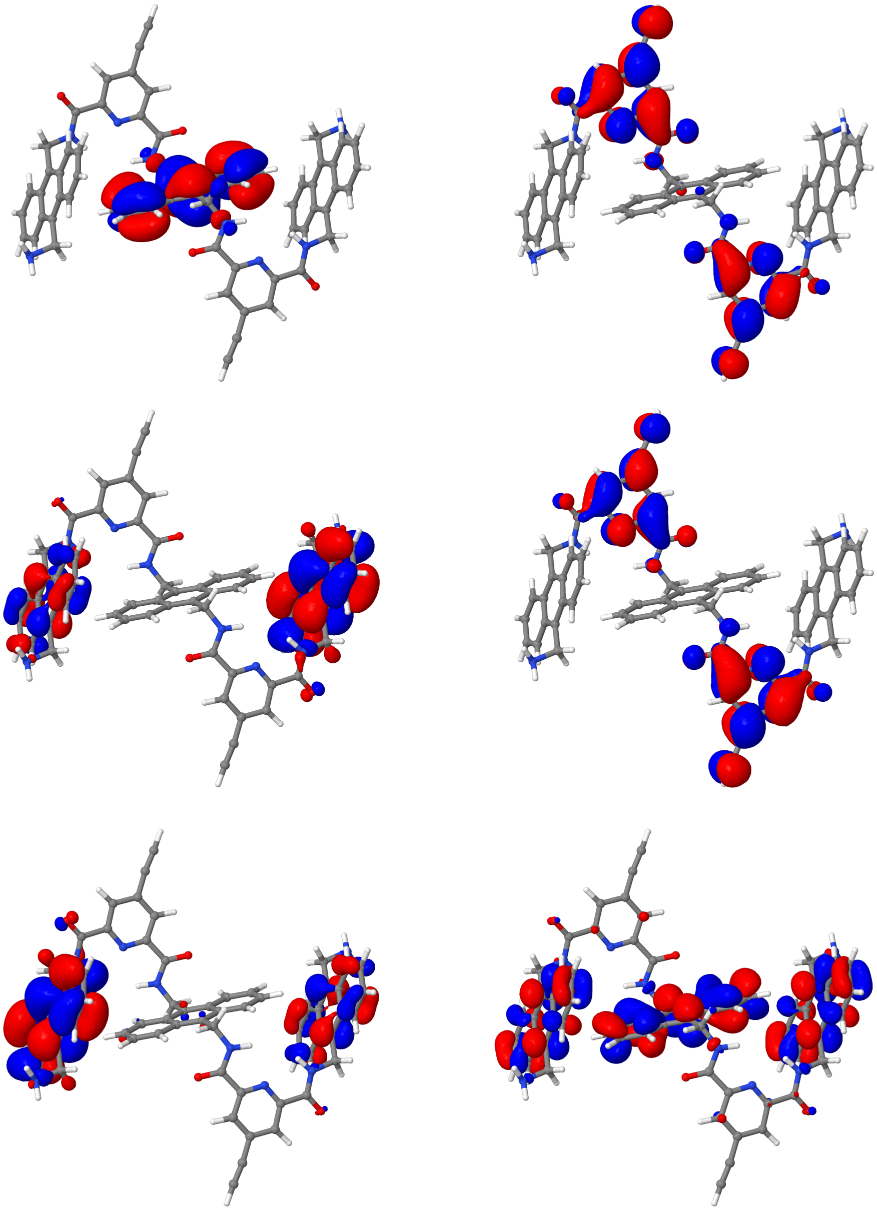

Supplementary Figure 6. Nearly degenerate frontier orbitals of the CH-π folded trimer: HOMO and LUMO (top), HOMO-1 and LUMO+1 (middle) and HOMO-2 and LUMO+2 (bottom).

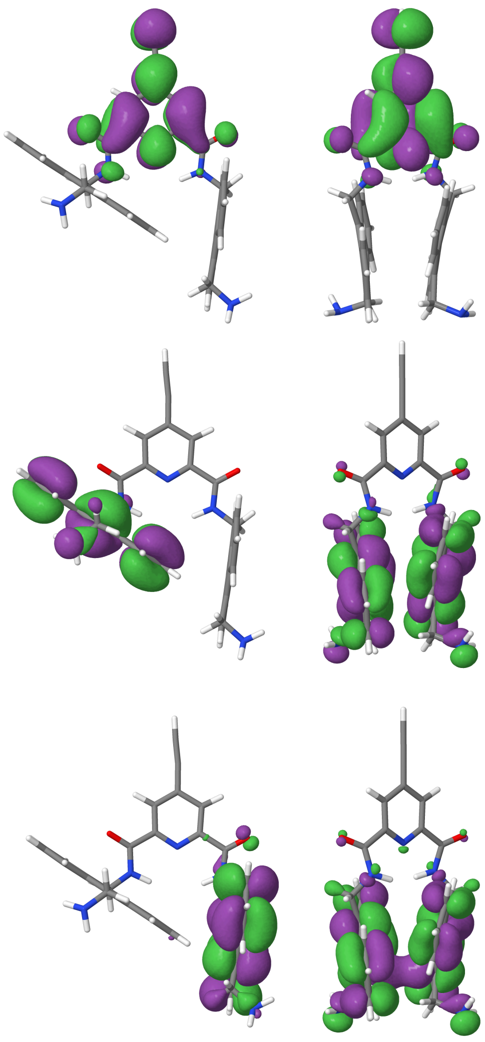

Supplementary Figure 7. LUMO (top), HOMO (middle) and HOMO-1 (bottom) orbitals for CH-π (left) and π-π (right) conformers of dimer-NH_2.

π-π

	L+5	L+4	L+3	L+2	L+1	LUMO	HOMO	H-1	H-2	H-3	H-4	H-5	HL GAP
**Dimer-NH_2**	-0,77	-0,85	-1,9	-2,03	-2,23	-2,29	-5,37	**-5,48**	-6,69	-6,73	-6,84	-6,9	3,08
**Trimer-NH_2**	-1,91	-2,08	-2,11	-2,17	-2,28	-2,29	-5,36	**-5,39**	**-5,45**	-6,61	-6,63	-6,77	3,07
**Tetramer-NH_2**	-2,08	-2,15	-2,17	-2,26	-2,27	-2,27	-5,32	**-5,36**	**-5,45**	**-5,5**	-6,53	-6,6	3,05
**Pentamer-NH_2**	-2,14	-2,17	-2,26	-2,26	-2,27	-2,27	-5,32	**-5,33**	**-5,38**	**-5,42**	**-5,46**	-6,52	3,05
						EA	IP						
**Dimer-NH_2**						-2,25	-5,31						
**Trimer-NH_2**						-2,30	-5,27						
**Tetramer-NH_2**						-2,26	-5,27						
**Pentamer-NH_2**						-2,33	-5,25						

CH-π

	L+5	L+4	L+3	L+2	L+1	LUMO	HOMO	H-1	H-2	H-3	H-4	H-5	HL GAP
**Dimer-NH_2**	-0,6	-0,75	-1,69	-2,02	-2,1	-2,17	-5,35	**-5,54**	-6,59	-6,73	-6,81	-6,85	3,18
**Trimer-NH_2**	-1,69	-1,98	-2,03	-2,09	-2,17	-2,17	-5,36	**-5,53**	**-5,53**	-6,6	-6,61	-6,73	3,19
**Tetramer-NH_2**	-2,07	-2,08	-2,16	-2,18	-2,2	-2,26	-5,36	**-5,37**	**-5,53**	**-5,62**	-6,6	-6,73	3,1
**Pentamer-NH_2**	-2,08	-2,15	-2,16	-2,18	-2,18	-2,24	-5,36	**-5,36**	**-5,52**	**-5,52**	**-5,6**	-6,6	3,12
						EA	IP						
**Dimer-NH_2**						-2,17	-5,34						
**Trimer-NH_2**						-2,16	-5,35						
**Tetramer-NH_2**						-2,29	-5,34						
**Pentamer-NH_2**						-2,27	-5,34						

Supplementary Table 1. Frontier orbital energies and adiabatic Electron Affinities (EA) and Ionization Potentials (IP) for the different conformers (eV)

Supplementary Note 1: Synthetic procedures

Anthracene-9,10-diylmethanamine[1] and dimethyl 4-bromopyridine-2,6-dicarboxylate[2] were synthesized according to literature procedures.

Synthesis of **A-NHBoc** and **1**

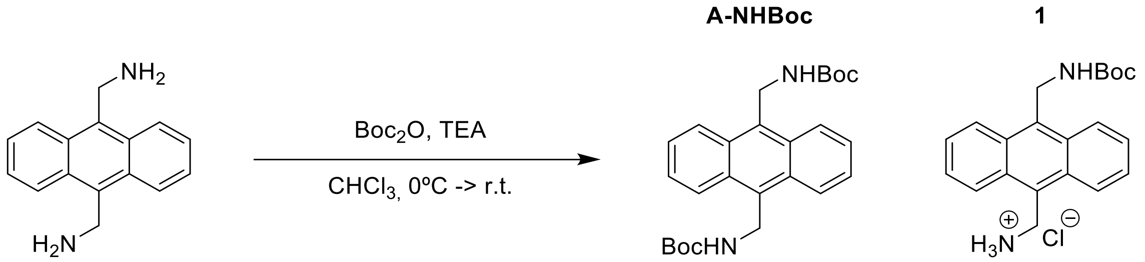

1.500 g of Anthracene-9,10-diylmethanamine (6.348 mmol) and 1.327 ml of TEA (9.521 mmol) were dissolved in 500 ml of $CHCl_3$ at 0ºC, a solution of 1.384 g of Boc_2O (6.348 mmol) in 250 ml of $CHCl_3$ was added dropwise during 2 hours. The mixture was stirred for 2 hours at room temperature, washed with water and chromatographed in gradient from $CHCl_3$ to $CHCl_3$/MeOH 97:3.

A-NHBoc was crystallized from boiling EtOAc. 434.6 mg (0.996 mmol) obtained, pale yellow crystals. 17%

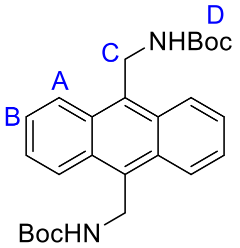

^{1}H NMR (400 MHz, $CDCl_3$) δ 8.39 (m, 4H, A), 7.59 (m, 4H, B), 5.32 (d, J = 5.3 Hz, 4H, C), 4.77 (s, 2H, NH), 1.45 (s, 18H, D); ^{13}C NMR (101 MHz, $CDCl_3$) δ 155.70, 130.55, 130.19, 126.44, 124.87, 79.81, 37.27, 28.54; MS (MALDI): m/z calcd. for $C_{26}H_{32}N_2O_4Na$ $[M+Na]^+$: 459.226, found 459.284; $C_{26}H_{32}N_2O_4K$ $[M+K]^+$: 475.200, found 475.283; $C_{26}H_{32}N_2O_4$ $[M]^+$: 436.236, found 436.250; UV-Vis (TCE 10^{-5}, 25ºC) λ max: 339.5, 356.0, 375.0, 396.0 nm; Fluorescence (TCE, 25ºC) A_{356nm}=0.1, λ max: 405.0, 425.5, 449.5, (sh) nm.

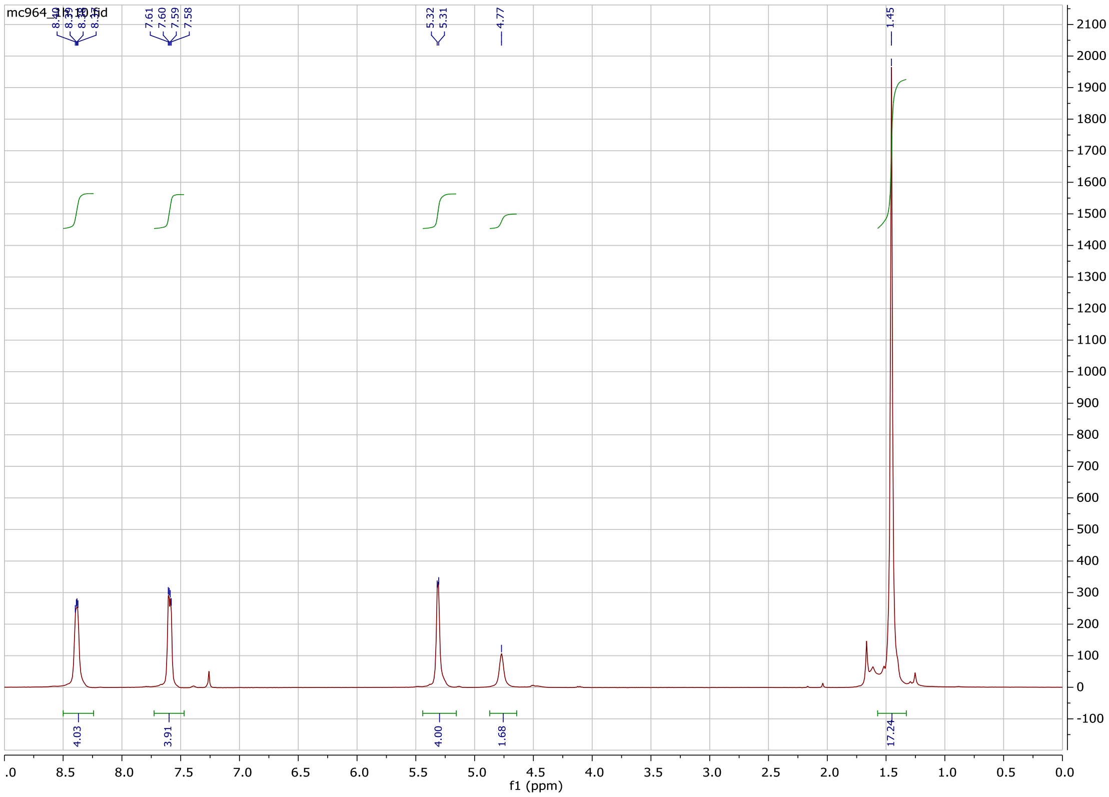
mc964_1h.10.fid
7.61
7.60
7.59
7.58
5.32
5.31
4.77
1.45
4.03
3.91
4.00
1.68
17.24
f1 (ppm)

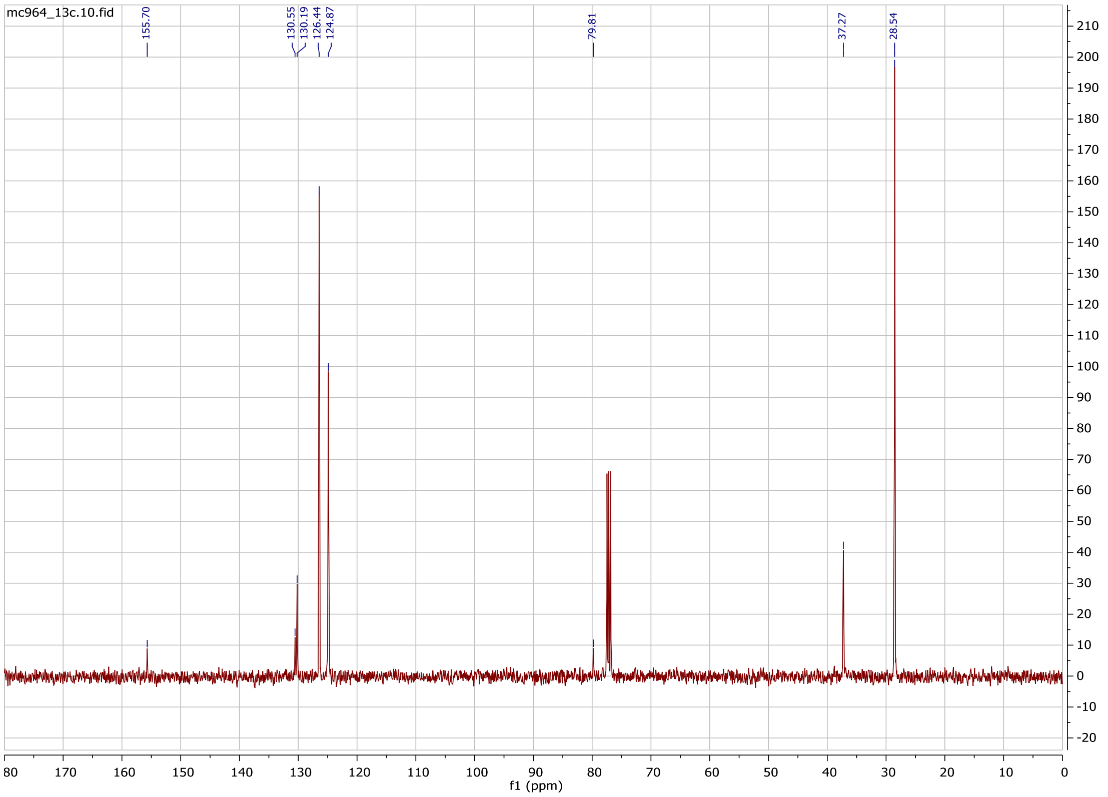
mc964_13c.10.fid
155.70
130.55
130.19
126.44
124.87
79.81
37.27
28.54
f1 (ppm)

1 was dissolved DCM / Et_2O at 0ºC and Et_2O saturated with HCl was added, the solvent was evaporated and the residue was suspended in few ml of MeOH at 0ºC and Et_2O was added. The precipitate was collected by filtration and washed with Et_2O.
1.1023 g (2.956 mmol) obtained, pale yellow powder. 47%

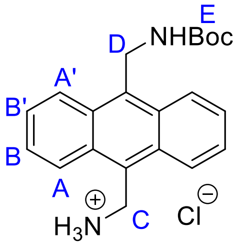

^{1}H NMR (400 MHz, DMSO-d_6) δ 8.60 (d, J = 8.7 Hz, 2H, A), 8.50 – 8.35 (m, 5H, A,NH_3^+), 7.67 (dt, J = 19.0, 6.9 Hz, 4H, B,B'), 7.54 (t, J = 6.0 Hz, 1H, NH), 5.19 (d, J = 5.5 Hz, 2H,D), 5.10 (s, 2H, C), 1.38 (s, 9H, E); ^{13}C NMR (101 MHz, DMSO-d_6) δ 155.64, 133.53, 129.95, 129.66, 126.47, 125.78, 125.75, 125.47, 124.52, 77.89, 36.53, 34.50, 28.22; MS (MALDI): m/z calcd. for $C_{21}H_{24}N_2O_2Na$ $[M+Na]^+$: 359.174, found 359.174.

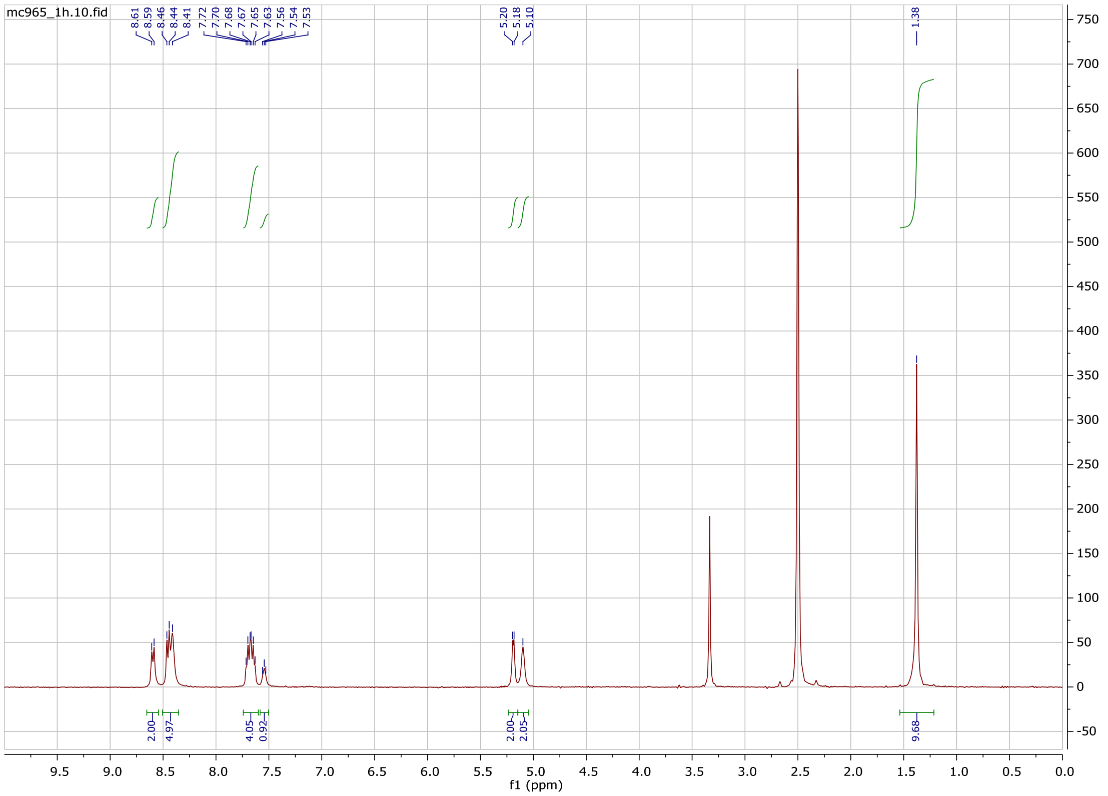

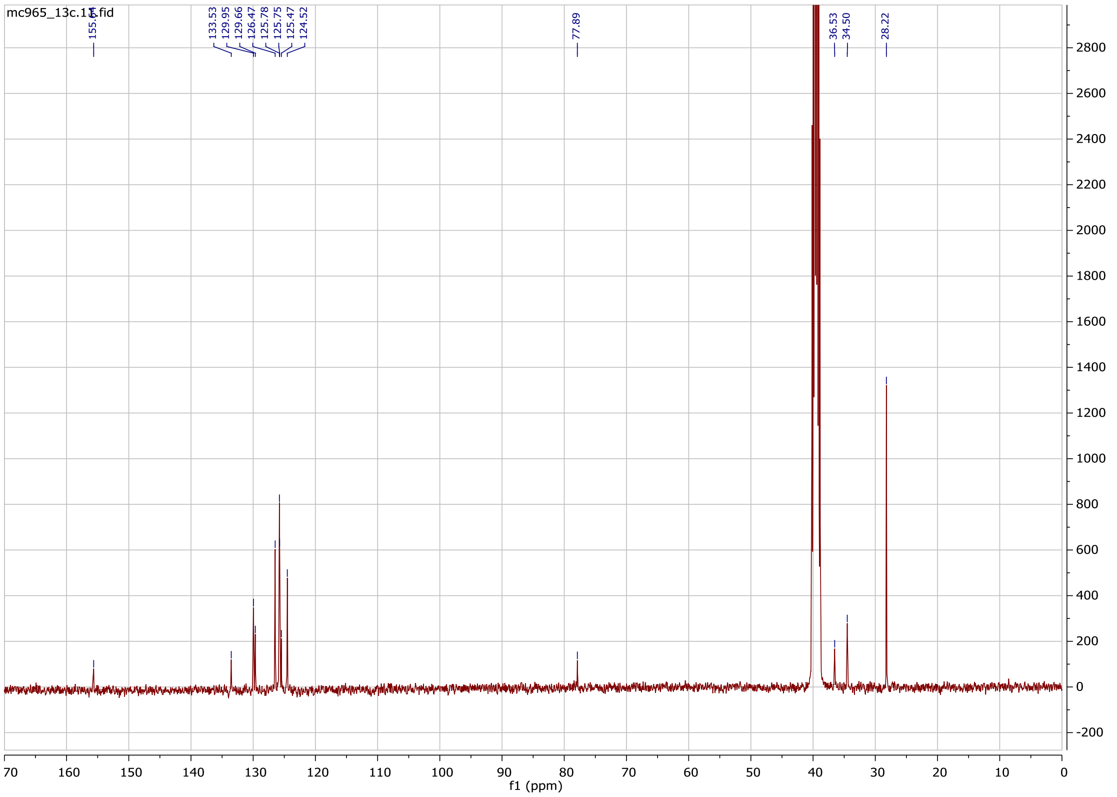
mc965_13c.11.fid
155.64
133.53
129.95
129.66
126.47
125.78
125.75
125.47
124.52
77.89
36.53
34.50
28.22
f1 (ppm)

Synthesis of **2**

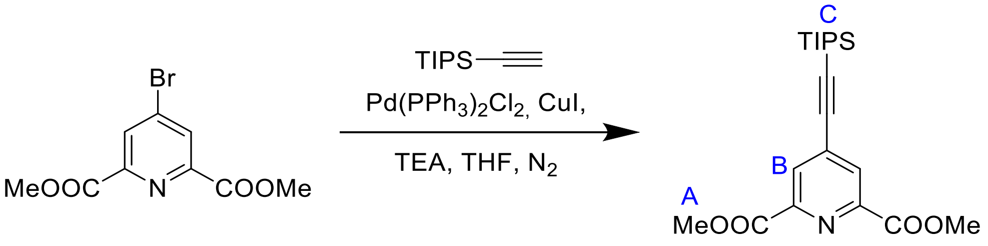

3.000 g of 4-bromopyridine-2,6-dicarboxylate (10.946 mmol), 480 mg of $Pd(PPh_3)_2Cl_2$ (0.684 mmol) and 83 mg of CuI (0.438 mmol) were suspended in 30 ml of dry THF and 10 ml of TEA, the mixture was degassed by means of 2 freeze-pump-thaw cycles and 3.68 ml of TIPS-acetylene (16.419) was added dropwise. The mixture was stirred for 2 hours, then was filtered over celite, solvent was evaporated and the product was purified by chromatography in gradient from Hex to Hex/EtOAc 85:15.
4.052 mg (10.789 mmol) obtained, pale yellow oil. 99%

^{1}H NMR (400 MHz, $CDCl_3$) δ 8.28 (s, 2H, B), 4.03 (s, 6H, A), 1.14 (m, 21H, C); ^{13}C NMR (101 MHz, $CDCl_3$) δ 164.89, 148.54, 134.55, 130.25, 102.40, 100.91, 53.48, 18.73, 11.29; MS (MALDI): m/z calcd. for $C_{20}H_{30}NO_4Si$ $[M+H]^+$: 376.194, found 376.077; $C_{20}H_{29}NO_4SiNa$ $[M+Na]^+$: 398.176, found 398.094.

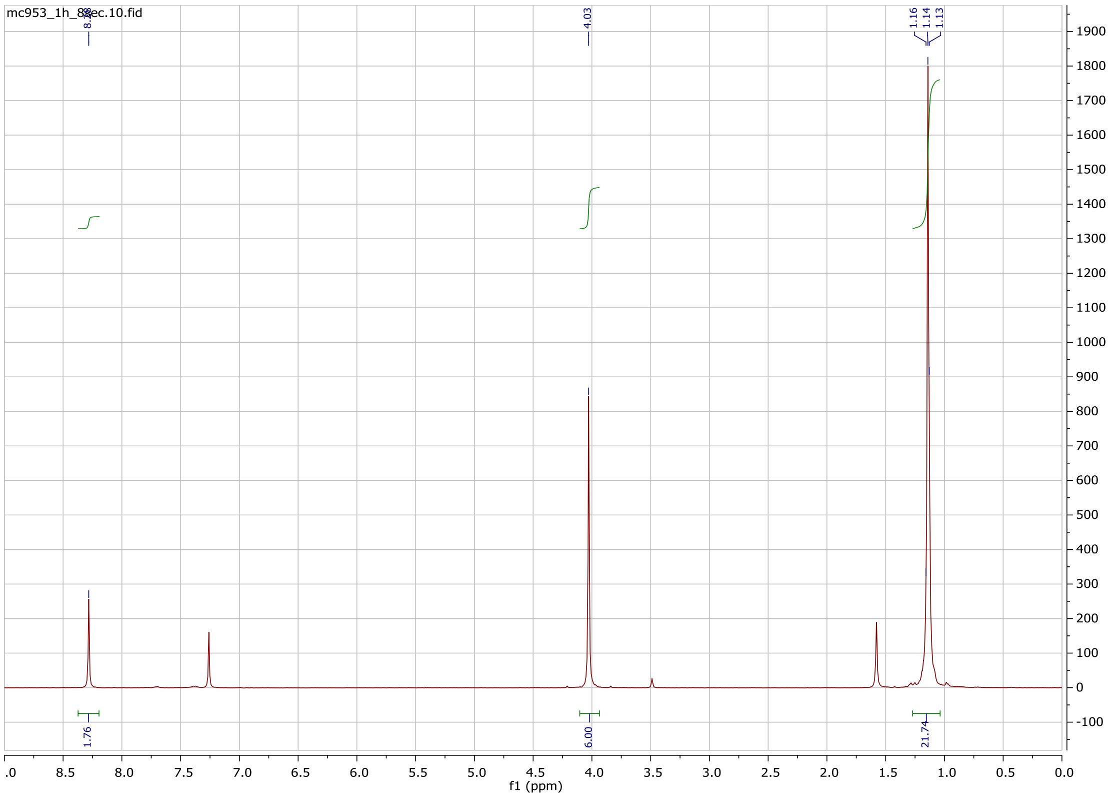

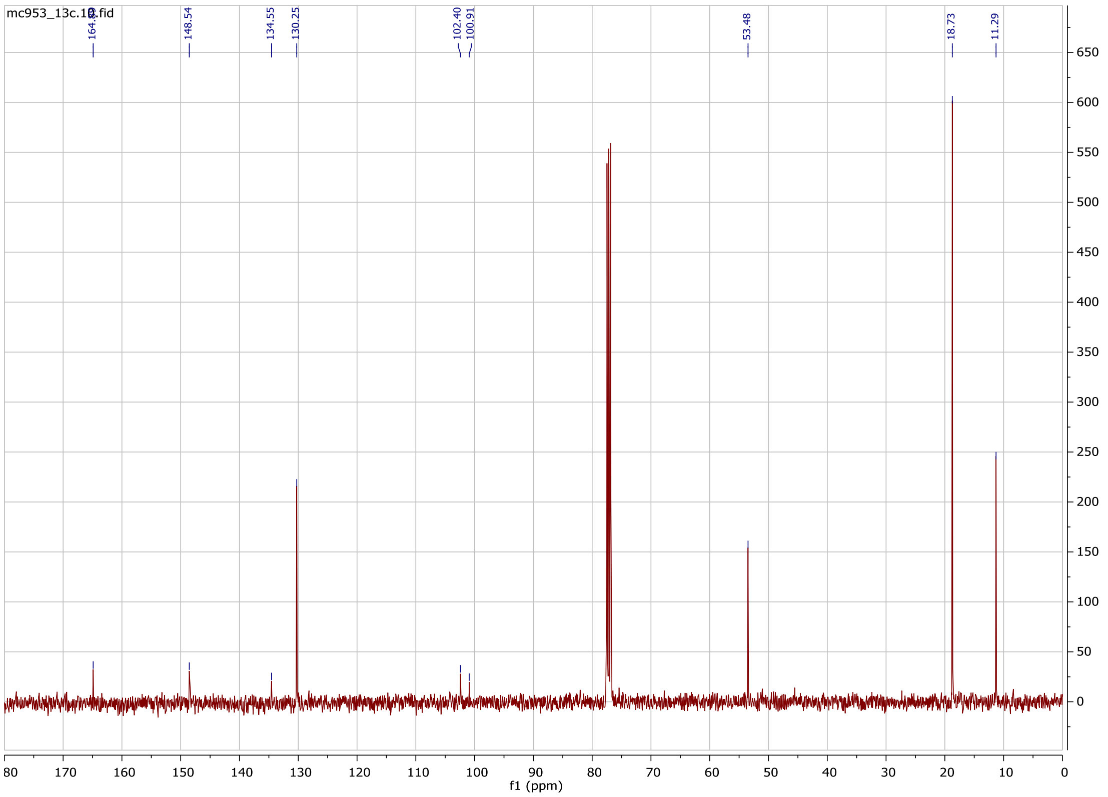

mc953_13c.10.fid
164.88
148.54
134.55
130.25
102.40
100.91
53.48
18.73
11.29
f1 (ppm)

Synthesis of **3**

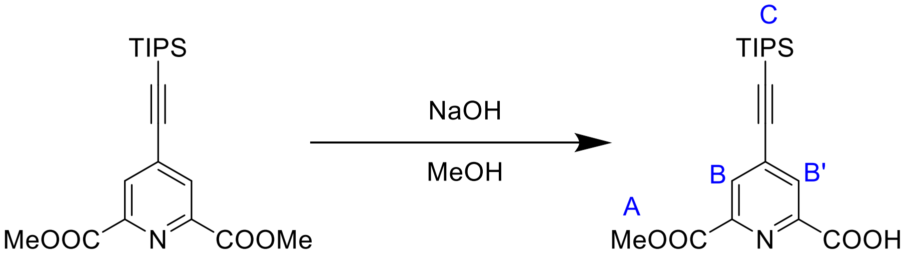

2.548 g of diester (6.785 mmol) were dissolved in 25 ml of MeOH and 271 mg of NaOH (6.785 mmol) were added in 5 ml of wet MeOH, the mixture was stirred for 3h. The residue was extracted with $CHCl_3$ / 0.5 M HCl. The mixture was separated by chromatography in gradient from $CHCl_3$ to $CHCl_3$ / MeOH 93:7.
1.619 g (4.478 mmol) obtained, white foam after co-evaporation with pentane. 66%

^{1}H NMR (400 MHz, DMSO-d_6) δ 8.10 (s, 1H, B), 8.08 (s, 1H, B), 3.94 (s, 3H, A), 1.11 (m, 21H, C); ^{13}C NMR (101 MHz, DMSO-d_6) δ 165.25, 164.60, 132.51, 128.89, 127.68, 102.94, 98.34, 53.08, 18.43, 10.56; MS (MALDI): m/z calcd. for $C_{19}H_{28}NO_4Si$ $[M+H]^+$: 362.179, found 362.034.

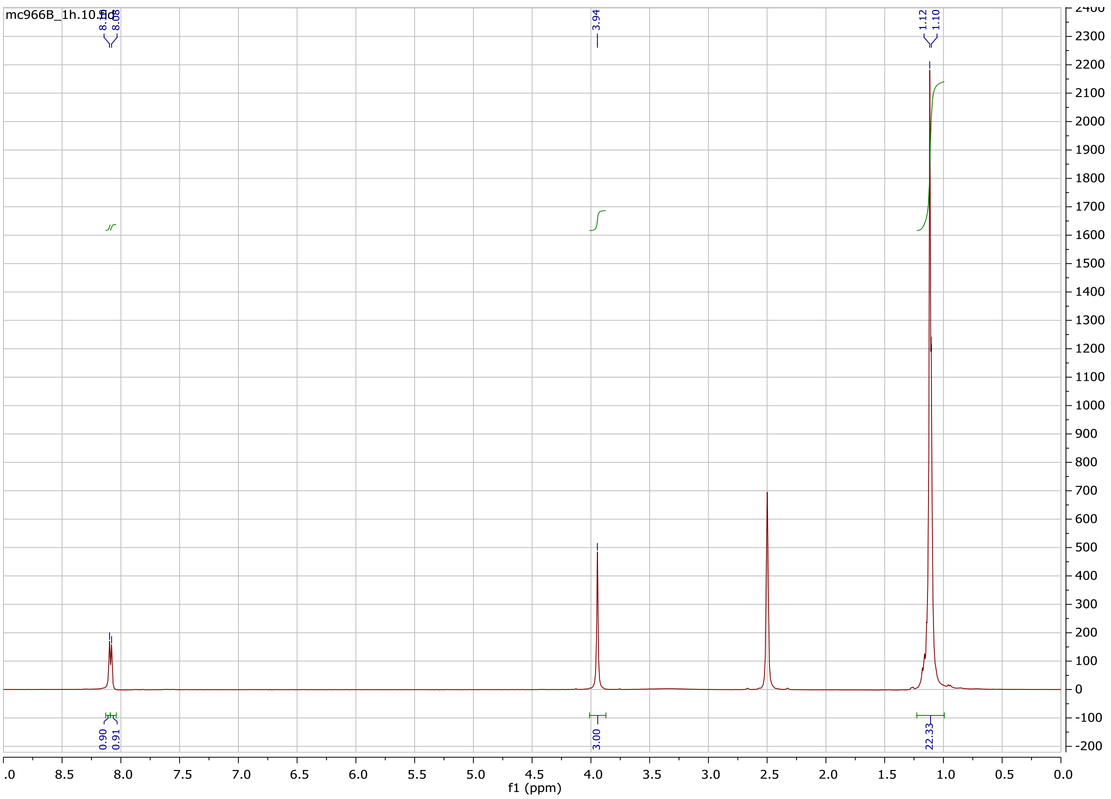

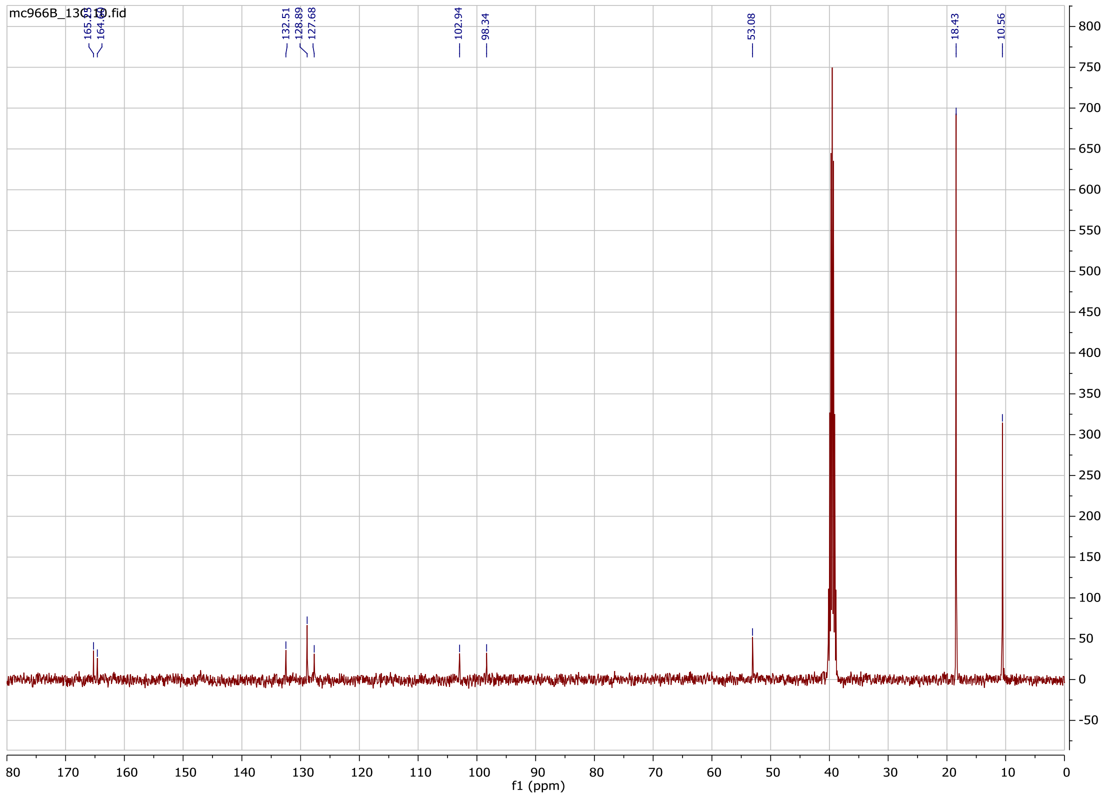

mc966B_13C.10.fid
165.13
164.63
132.51
128.89
127.68
102.94
98.34
53.08
18.43
10.56
f1 (ppm)

Synthesis of **4**

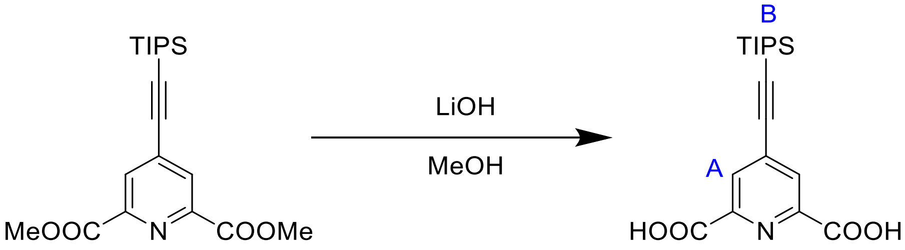

1.467 g of diester (3.907 mmol) were dissolved in 20 ml of MeOH, 492 mg of LiOH (11.721 mmol) dissolved in 10 ml of MeOH / H_2O 8:2 were added, the solution was stirred for 2h, The solvent was evaporated and the residue was suspended in water, the pH was adjusted to <2 with 2N HCl and the diacid was collected by filtration, washed with water and finally with hexane.

1.279 g (3.681 mmol) obtained, white solid. 94%

^{1}H NMR (400 MHz, DMSO-d_6) δ 8.11 (s, 2H, A), 1.11 (m, 21H, B); ^{13}C NMR (101 MHz, DMSO-d_6) δ 164.96, 149.17, 132.49, 128.71, 102.81, 98.44, 18.41, 10.55; MS (MALDI): m/z calcd. for $C_{18}H_{26}NO_4Si$ $[M+H]^+$: 348.163, found 347.992.

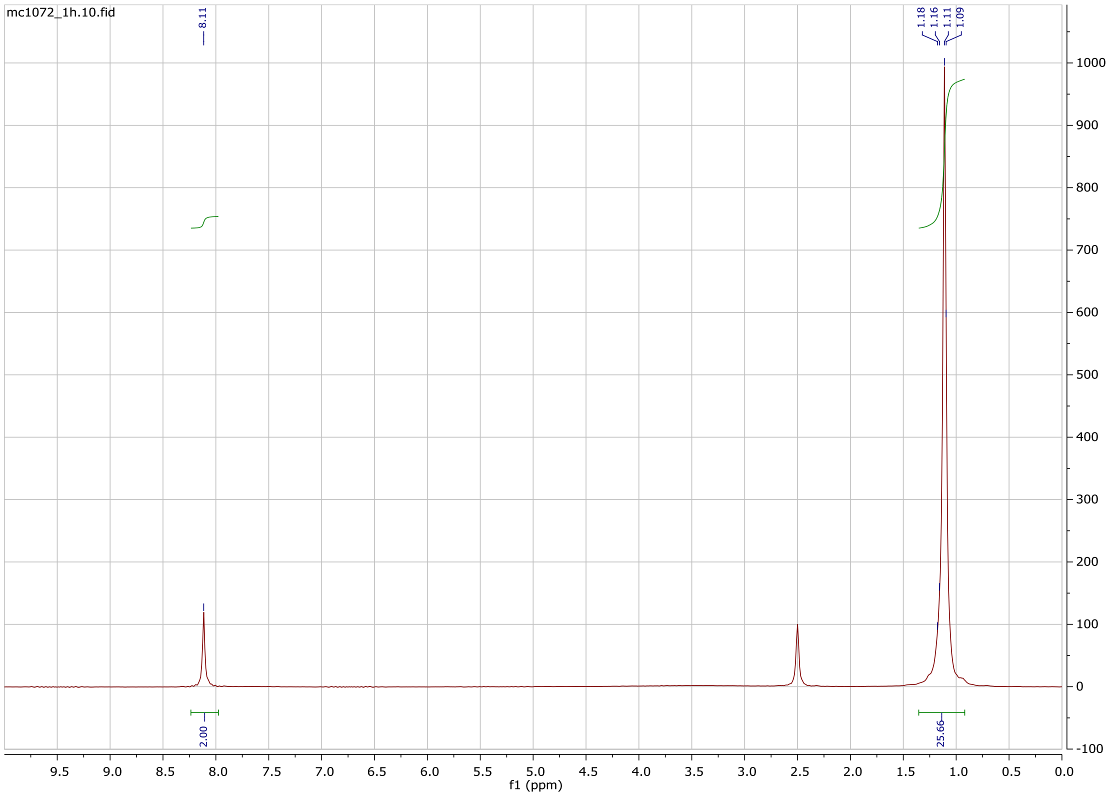

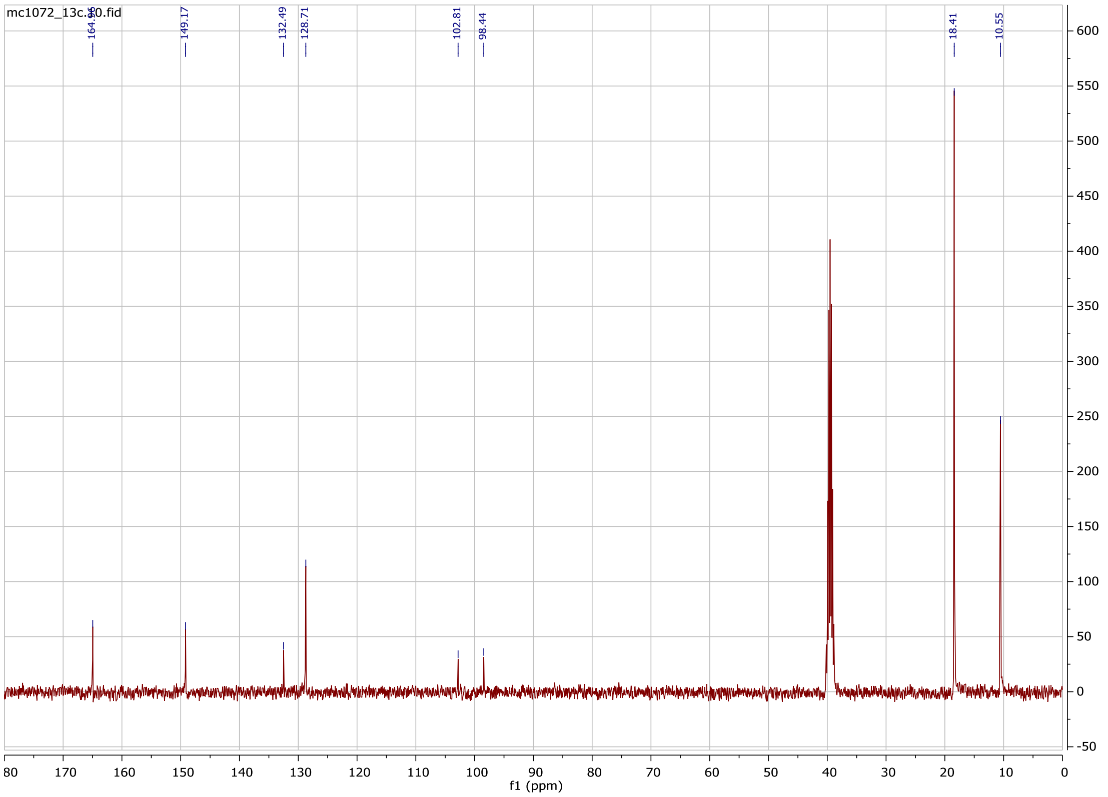
mc1072_13c.10.fid
164.95
149.17
132.49
128.71
102.81
98.44
18.41
10.55
f1 (ppm)

Synthesis of **5**

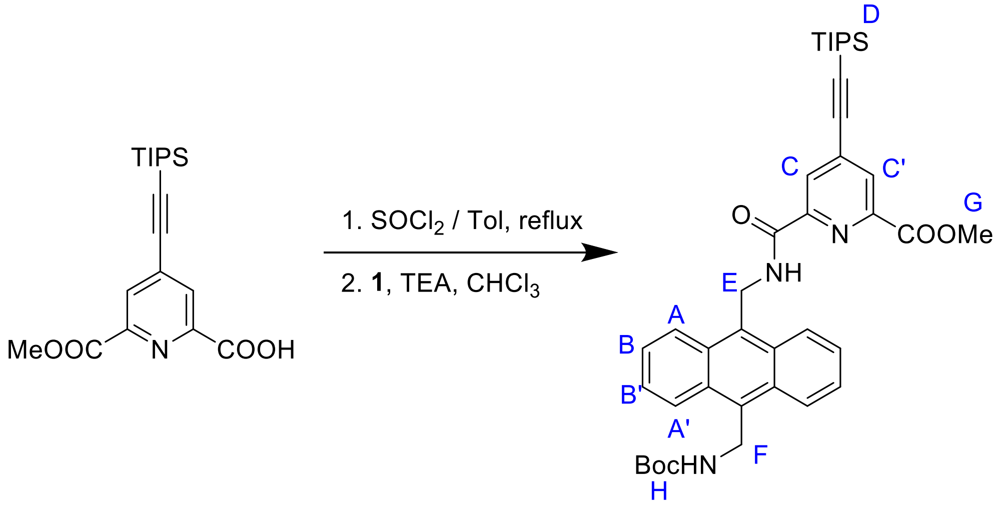

640 mg of **3** (1.770 mmol) were refluxed in 5 ml of Toluene and 3 ml of $SOCl_2$ for 3h30' under N_2 atmosphere. The solvent was evaporated and the residue was dissolved in 5 ml of dry DCM under N_2 at 0ºC. 600 mg of **1** (1.609 mmol) were added, followed by 673 µl of TEA (4.827 mmol) and the reaction was stirred overnight. The reaction was diluted with DCM and washed with 5% HCl.

The product was purified by chromatography in $CHCl_3$ and triturated with Pentane.

879.7 mg (1.294 mmol) obtained, lemon yellow powder. 80%

^{1}H NMR (400 MHz, DMSO-d_6) δ 8.68 (d, J = 5.8 Hz, 1H, NH amide), 8.57 (d, J = 7.7 Hz, 2H, A), 8.51 (d, J = 8.2 Hz, 2H, A), 8.17 (s, 1H, C'), 8.03 (s, 1H, C), 7.60 (dd, J = 7.8, 4.0 Hz, 4H, B,B'), 7.44 (t, J = 5.4 Hz, 1H, NH Boc), 5.59 (d, J = 5.7 Hz, 2H, E), 5.16 (d, J = 5.4 Hz, 2H, F), 3.73 (s, 3H, G), 1.38 (s, 9H, H), 1.11 (m, 21H, D); ^{13}C NMR (101 MHz, DMSO-d_6) δ 163.62, 162.05, 155.61, 150.43, 147.13, 132.83, 131.77, 129.80, 129.76, 128.48, 126.71, 126.02, 125.63, 125.52, 124.85, 102.76, 98.91, 77.77, 52.72, 36.54, 35.50, 28.22, 18.42, 10.55; MS (MALDI): m/z calcd. for $C_{40}H_{49}N_3O_5SiNa$ $[M+Na]^+$: 702.334, found 702.358.

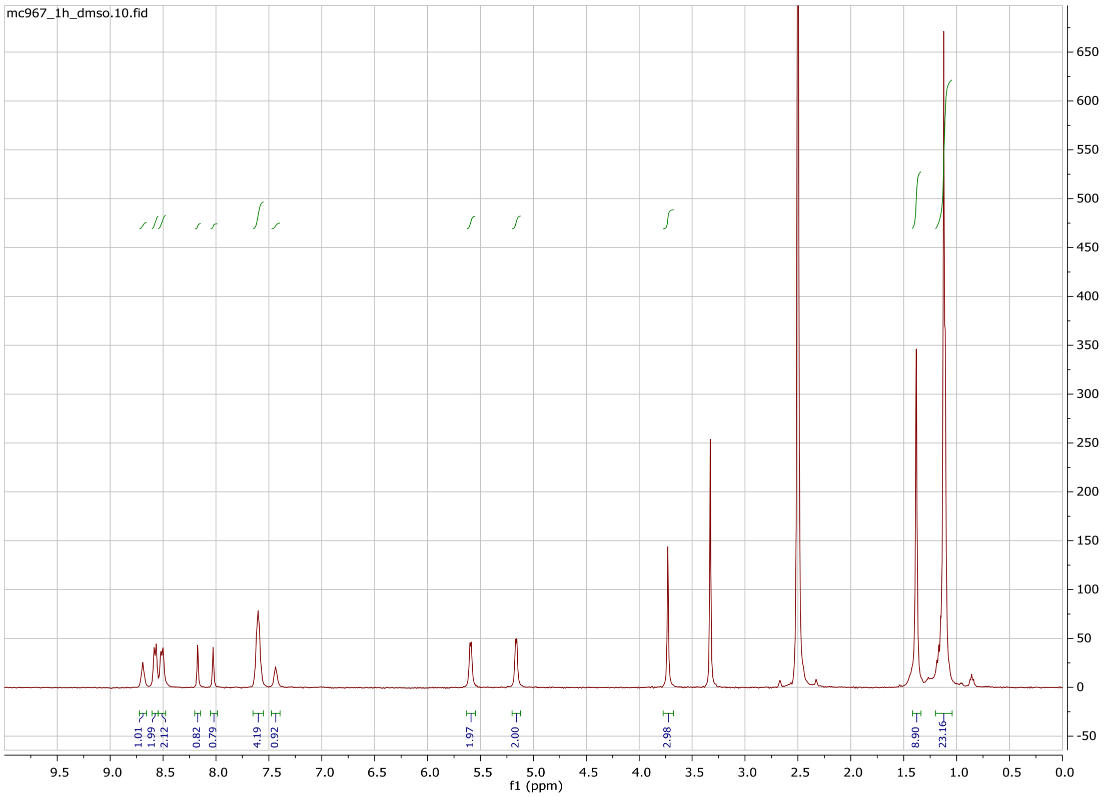
mc967_1h_dmso.10.fid
1.01
1.99
2.12
0.82
0.79
4.19
0.92
1.97
2.00
2.98
8.90
23.16
f1 (ppm)

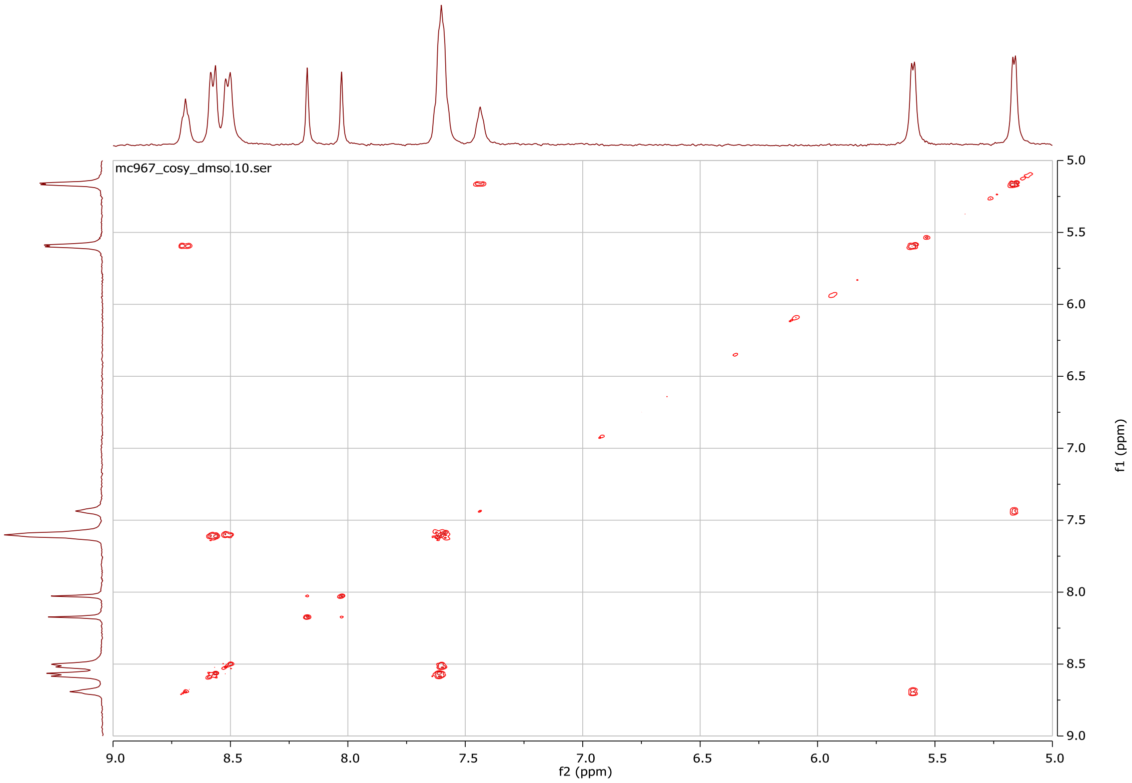
mc967_cosy_dmso.10.ser
f2 (ppm)
f1 (ppm)

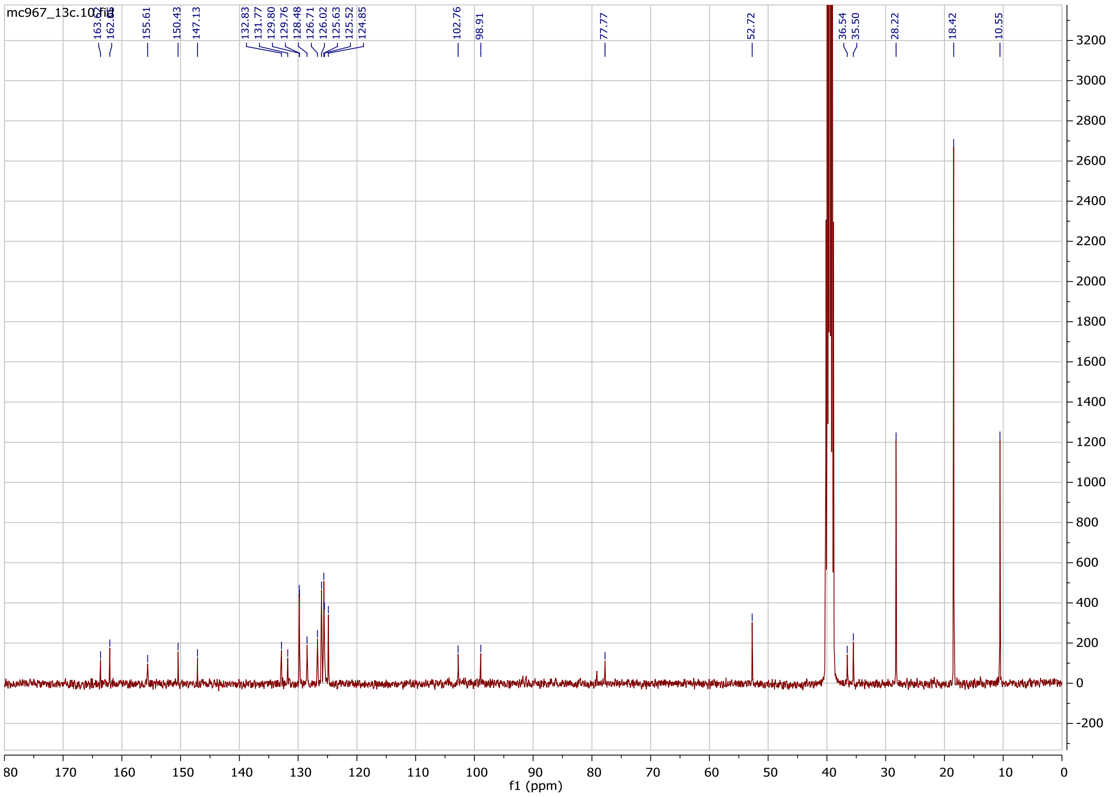
mc967_13c.10.fid
155.61
150.43
147.13
132.83
131.77
129.80
129.76
128.48
126.71
126.02
125.63
125.52
124.85
102.76
98.91
77.77
52.72
36.54
35.50
28.22
18.42
10.55
f1 (ppm)

Synthesis of **6**

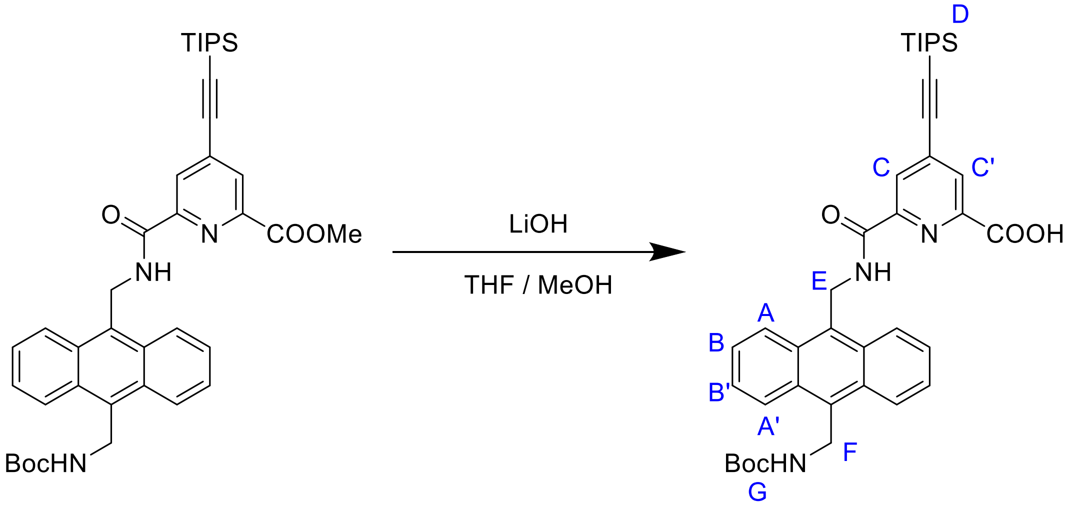

850 mg of **5** (1.250 mmol) were dissolved in 10 ml THF / 10 ml MeOH and 79 mg of LiOH (1.875 mmol) were added in 5 ml of wet MeOH. The mixture was stirred for 2 hours, then the pH was adjusted to ~3 with 5% HCl and the solvent was evaporated, the residue was suspended in water and filtered, the solid was washed with water and finally with 2 x 5 ml of cold MeOH.
773 mg (1.160 mmol) obtained, lemon yellow powder. 93%

^{1}H NMR (400 MHz, DMSO-d$_6$) δ 13.14 (s, 1H, COOH), 9.25 (d, J = 5.7 Hz, 1H, NH amide), 8.53 (t, J = 8.6 Hz, 4H, A,A'), 8.25 (s, 1H, C'), 8.07 (s, 1H, C), 7.60 (dd, J = 10.1, 5.7 Hz, 4H, B,B'), 7.45 (t, J = 6.2 Hz, 1H, NH Boc), 5.61 (d, J = 5.3 Hz, 2H, E), 5.18 (d, J = 5.4 Hz, 2H, F), 1.38 (s, 9H, G), 1.12 (d, J = 5.8 Hz, 21H, D); ^{13}C NMR (101 MHz, DMSO-d$_6$) δ 164.06, 161.98, 155.62, 149.58, 133.17, 131.89, 129.97, 129.83, 129.50, 127.97, 126.78, 126.10, 125.63, 125.58, 124.82, 102.89, 98.89, 79.16, 77.79, 36.51, 35.76, 28.23, 18.43, 10.56; MS (MALDI): m/z calcd. for $C_{39}H_{47}N_3O_5SiNa$ $[M+Na]^+$: 688.318, found 688.516.

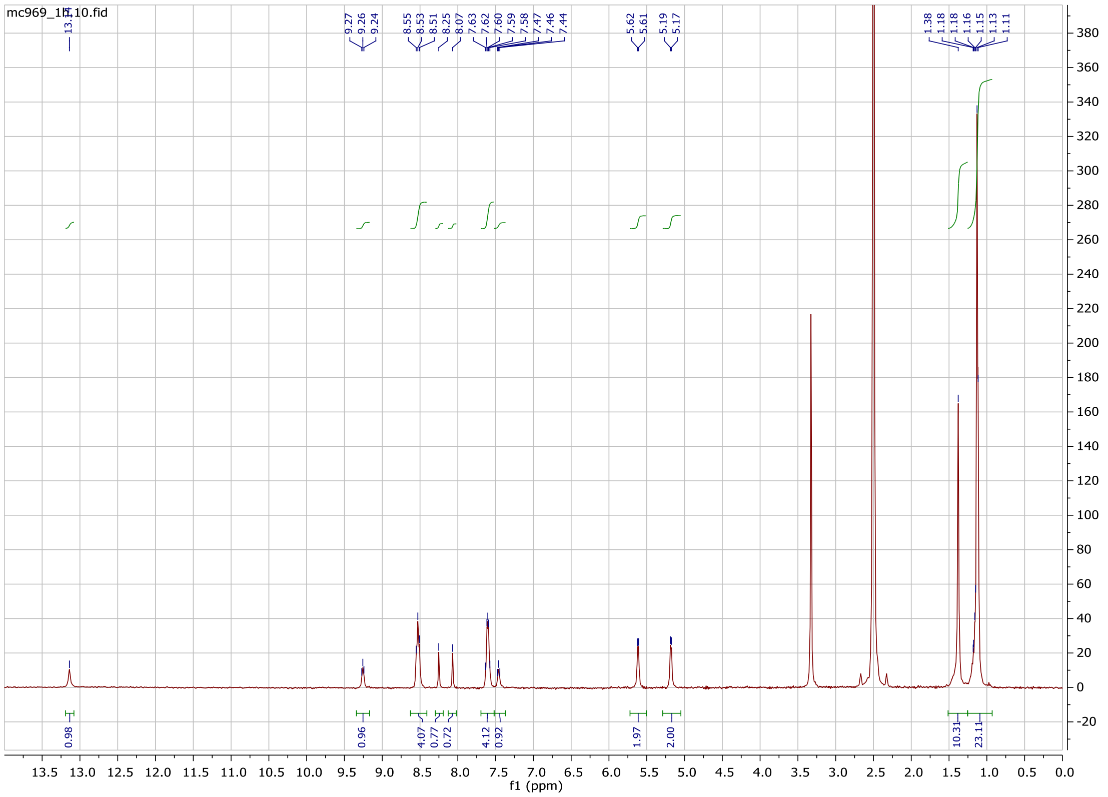
mc969_1H.10.fid
f1 (ppm)

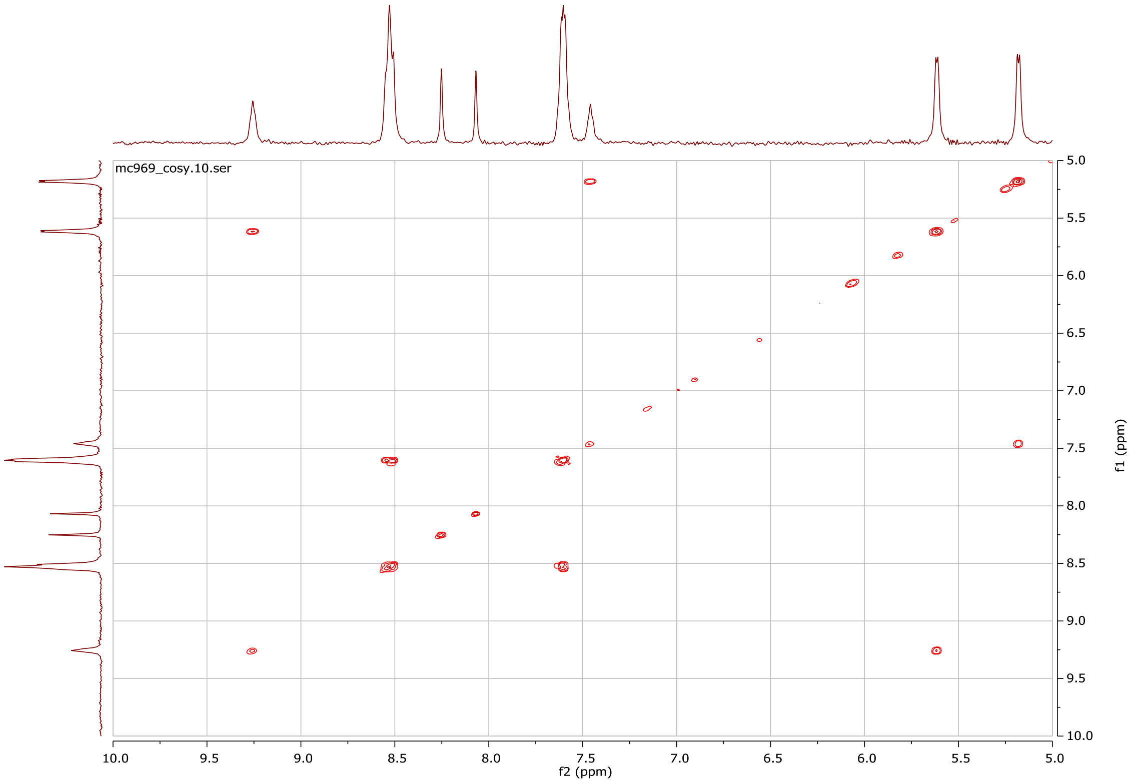
mc969_cosy.10.ser
f2 (ppm)
f1 (ppm)

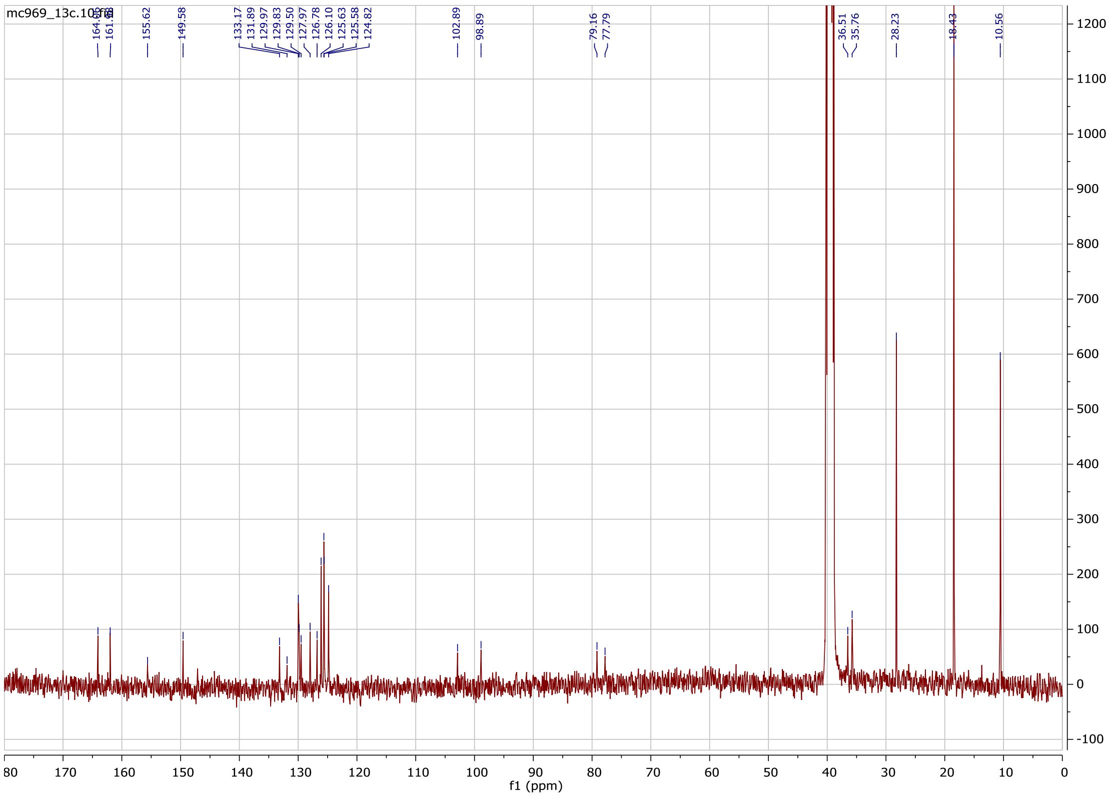
mc969_13c.10.fid
164.06
161.98
155.62
149.58
133.17
131.89
129.97
129.83
129.50
127.97
126.78
126.10
125.63
125.58
124.82
102.89
98.89
79.16
77.79
36.51
35.76
28.23
18.43
10.56
f1 (ppm)

Synthesis of **Dimer-NHBoc**

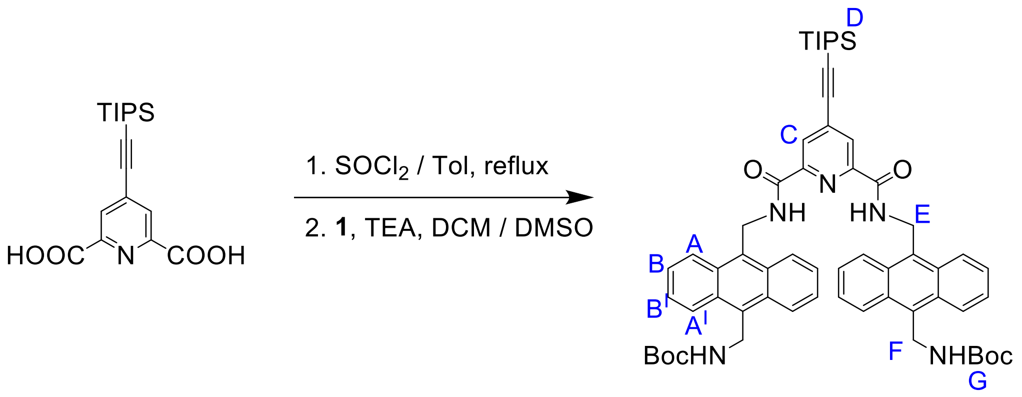

100 mg of **4** (0.288 mmol) were refluxed in 5 ml of Toluene and 3 ml of $SOCl_2$ for 3h30' under N_2 atmosphere. The solvent was evaporated and the residue was dissolved in 3 ml of dry DCM under N_2 at 0ºC. 194 mg of **1** (0.576 mmol) were added, followed by 140 µl of TEA (1.007 mmol) and the reaction was stirred overnight. The reaction was diluted with $CHCl_3$ and washed with 5% HCl.
The product was purified by chromatography in $CHCl_3$ and triturated with Pentane.
157 mg (0.1596 mmol) obtained, lemon yellow powder. 55%

^{1}H NMR (500 MHz, DMSO-d_6) δ 9.53 (s, 2H, NH amide), 8.49 (d, J = 8.9 Hz, 8H, A,A'), 8.10 (s, 2H,C), 7.54 (t, J = 8.1 Hz, 4H, *B*), 7.38 (s, 2H, NH carbamate), 7.33 (t, J = 7.6 Hz, 4H, *B*), 5.49 (s, 4H, E), 5.14 (d, J = 5.3 Hz, 4H, F), 1.39 (s, 18H, G), 1.12 (d, J = 6.5 Hz, 21H, D); ^{13}C NMR (126 MHz, DMSO-d_6) δ 162.19, 155.59, 149.33, 132.78, 131.54, 129.87, 129.67, 126.00, 125.60, 125.46, 125.27, 125.16, 103.23, 98.25, 77.77, 36.46, 35.58, 28.23, 18.50, 18.41, 10.65, 10.55; MS (MALDI): m/z calcd. for $C_{60}H_{69}N_5O_6SiNa$ $[M+Na]^+$: 1006.491, found 1006.532; UV-Vis (TCE 10^{-5}, 25ºC) λ max: 340.0, 357.0, 376.0, 397.0 nm; Fluorescence (TCE, 25ºC) A_{356nm}=0.1, λ max: 406.5, 427.0, 450.5, (sh) nm.

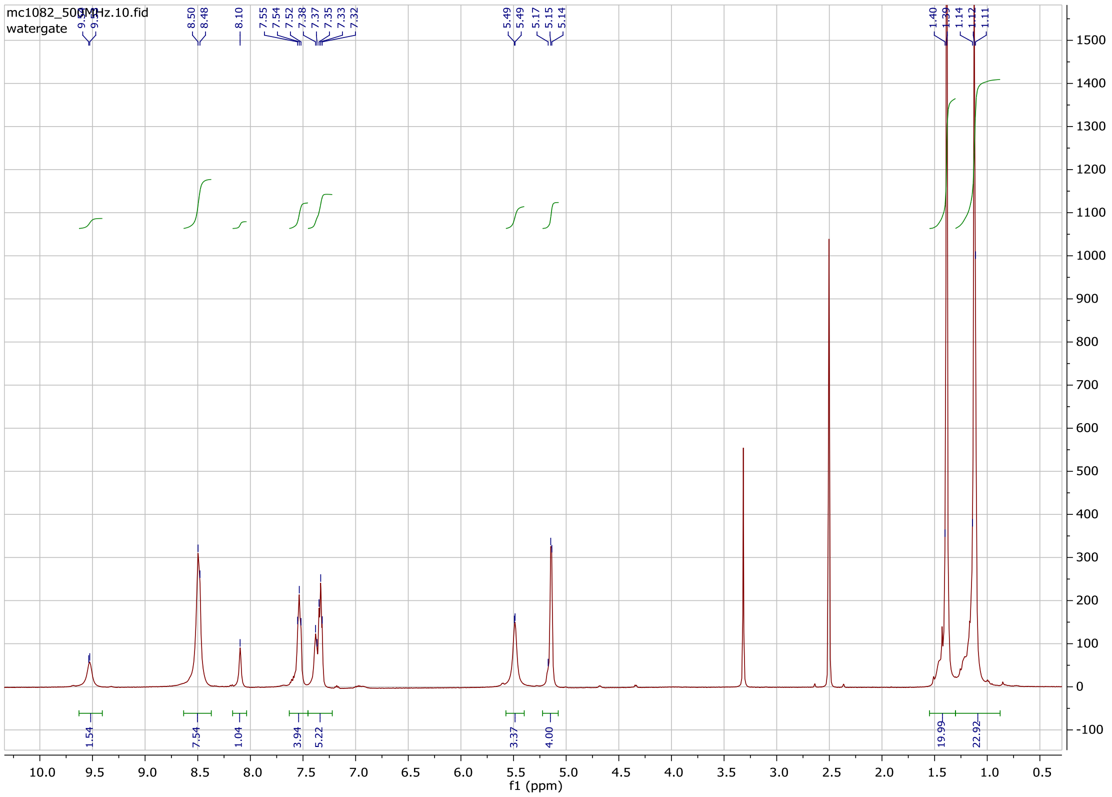
mc1082_500MHz.10.fid
watergate
f1 (ppm)

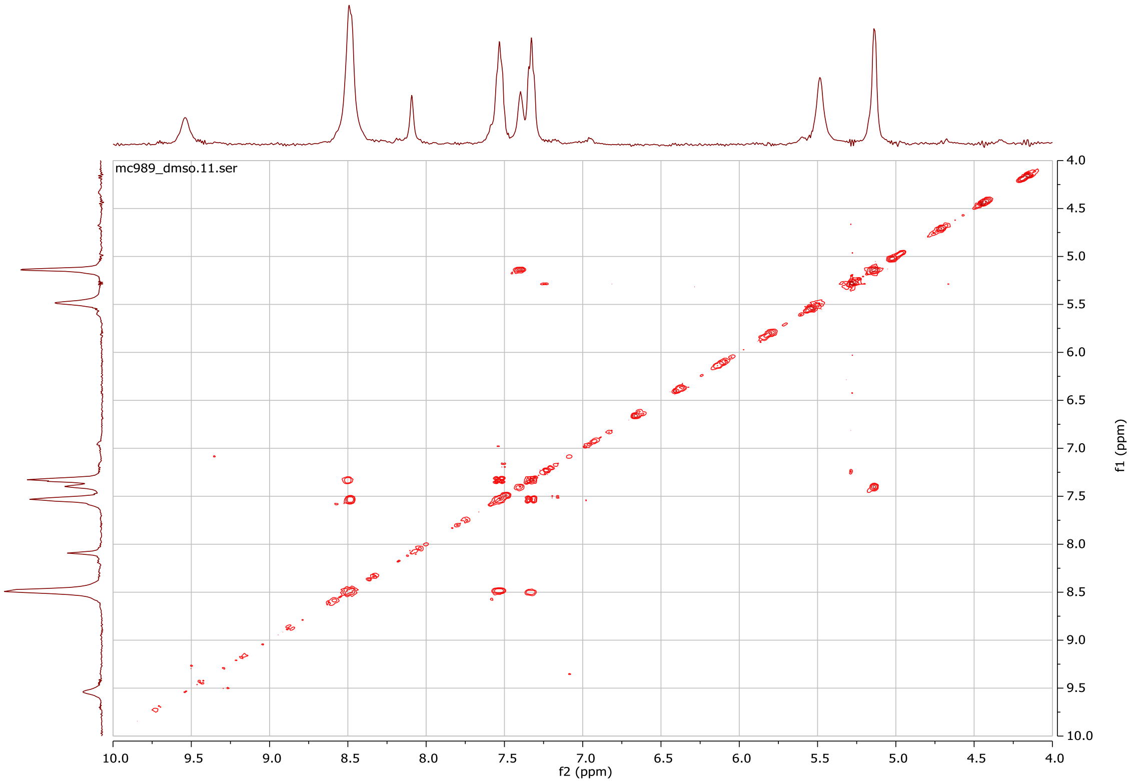
mc989_dmso.11.ser
f2 (ppm)
f1 (ppm)

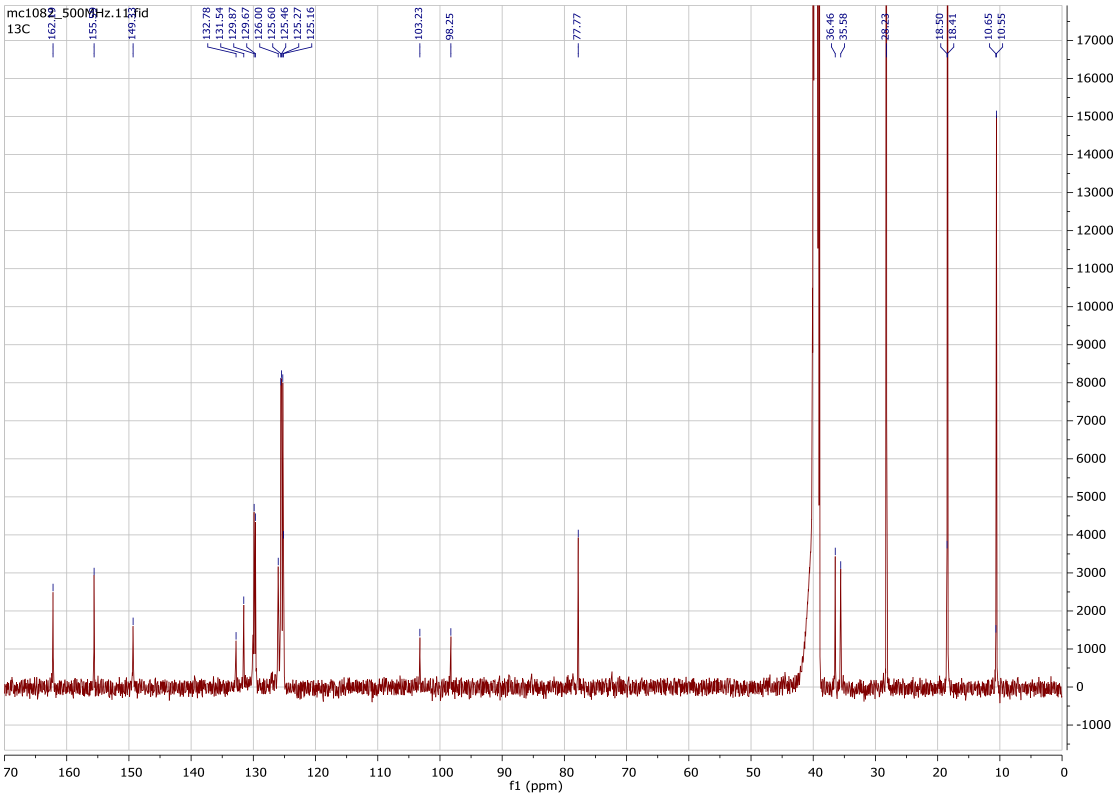

mc1082_500MHz.11.fid
13C
162.1
155.
149.13
132.78
131.54
129.87
129.67
126.00
125.60
125.46
125.27
125.16
103.23
98.25
77.77
36.46
35.58
28.23
18.50
18.41
10.65
10.55
f1 (ppm)

Synthesis of **Trimer-NHBoc**

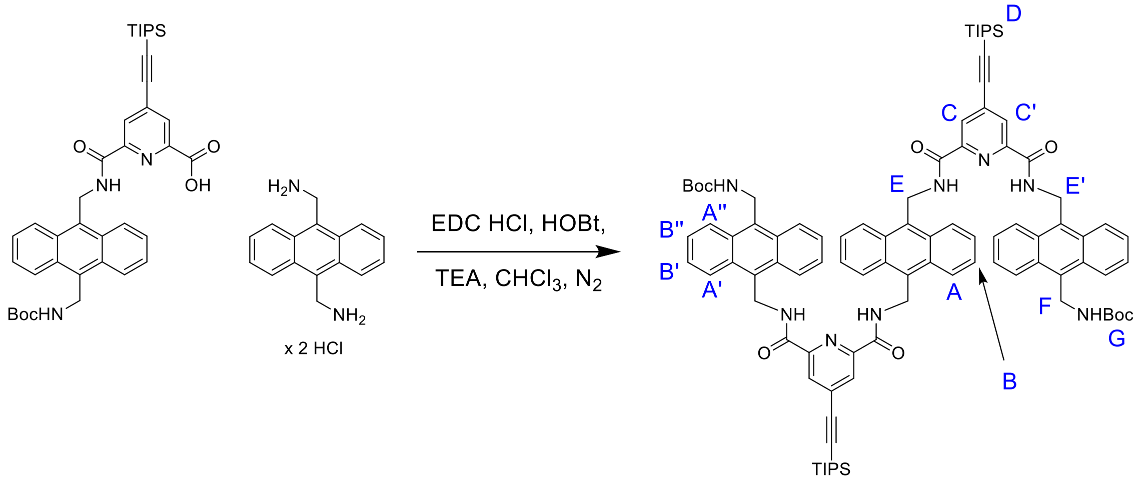

620 mg of **6** (0.931 mmol) and 132 mg HOBt (0.973 mmol) were vacuum dried for 1 hour, then were suspended in 10 ml of dry $CHCl_3$ under N_2 at 0ºC. 187 mg of EDC•HCl (0.973 mmol) were added and the mixture was stirred at 0ºC for 40 minutes until it changes into a clear solution. 100 mg of Anthracene-9,10-diylmethanamine (0.423 mmol) were added, followed by 136 µl of TEA (0.973 mmol) and the mixture was allowed to warm to room temperature and was stirred overnight with protection from light. The product was purified by column chromatography in $CHCl_3$ and was triturated with Hexane.

275.3 mg (0.180 mmol) obtained, pale yellow solid. 42%

^{1}H NMR (500 MHz, DMSO-d_6) δ 9.50 (s, 4H, NH amide), 8.60 – 8.33 (m, 12H, *A*), 8.11 (d, J = 5.1 Hz, 4H, C,C'), 7.49 (t, J = 7.9 Hz, 4H, B), 7.34 (t, J = 6.8 Hz, 2H, NH Boc), 7.29 (dd, J = 9.4, 5.2 Hz, 8H, B), 5.47 (s, 8H, E,E'), 5.11 (d, J = 5.5 Hz, 4H,F), 1.36 (s, 18H, G), 1.11 (m, 42H, D); ^{13}C NMR (126 MHz, DMSO-d_6) δ 162.20, 155.56, 149.33, 132.81, 131.58, 130.48, 130.01, 129.84, 129.64, 126.00, 125.60, 125.53, 125.39, 125.24, 125.15, 103.21, 98.28, 77.73, 36.41, 35.58, 28.20, 18.41, 10.54; MS (MALDI): m/z calcd. for $C_{94}H_{106}N_8O_8Si_2Na$ $[M+Na]^+$: 1554.760, found 1554.719; UV-Vis (TCE 10^{-5}, 25ºC) λ max: 340.0, 357.5, 376.5, 397.5 nm; Fluorescence (TCE, 25ºC) A_{356nm}=0.1, λ max:,407.0, 427.5, 452.0, (sh) nm.

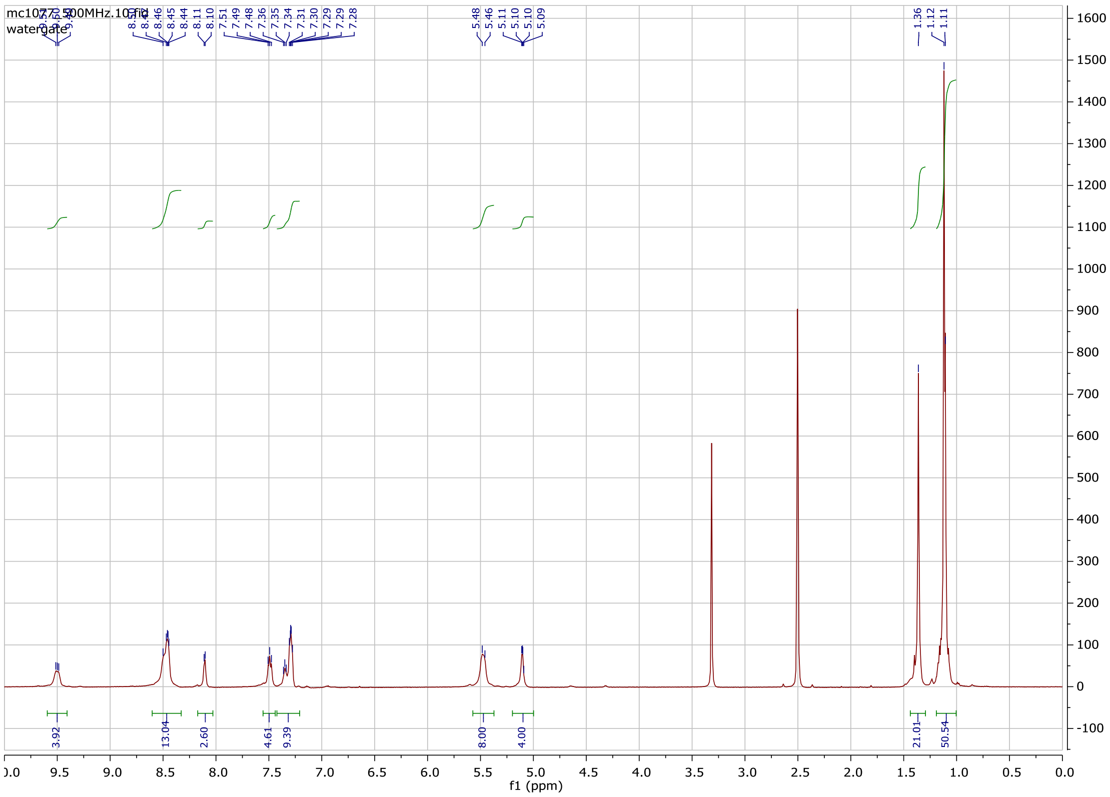
mc1077.500MHz.10.fid
watergate
f1 (ppm)

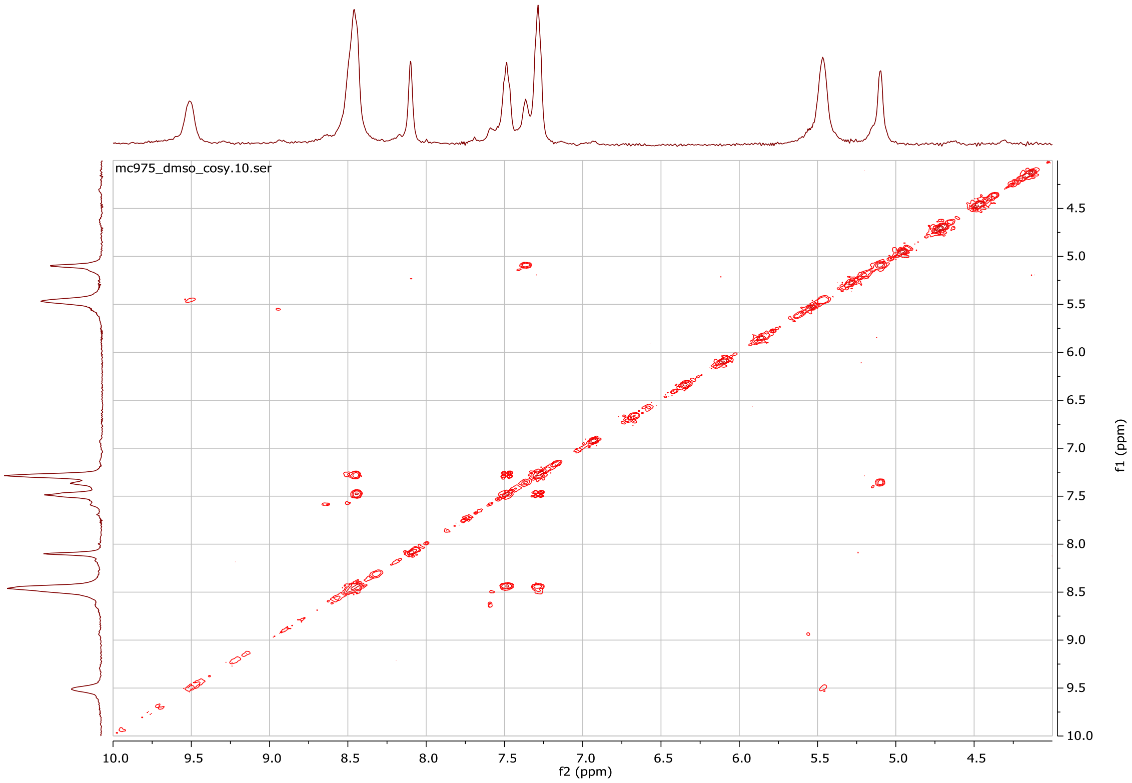
mc975_dmso_cosy.10.ser
f2 (ppm)
f1 (ppm)

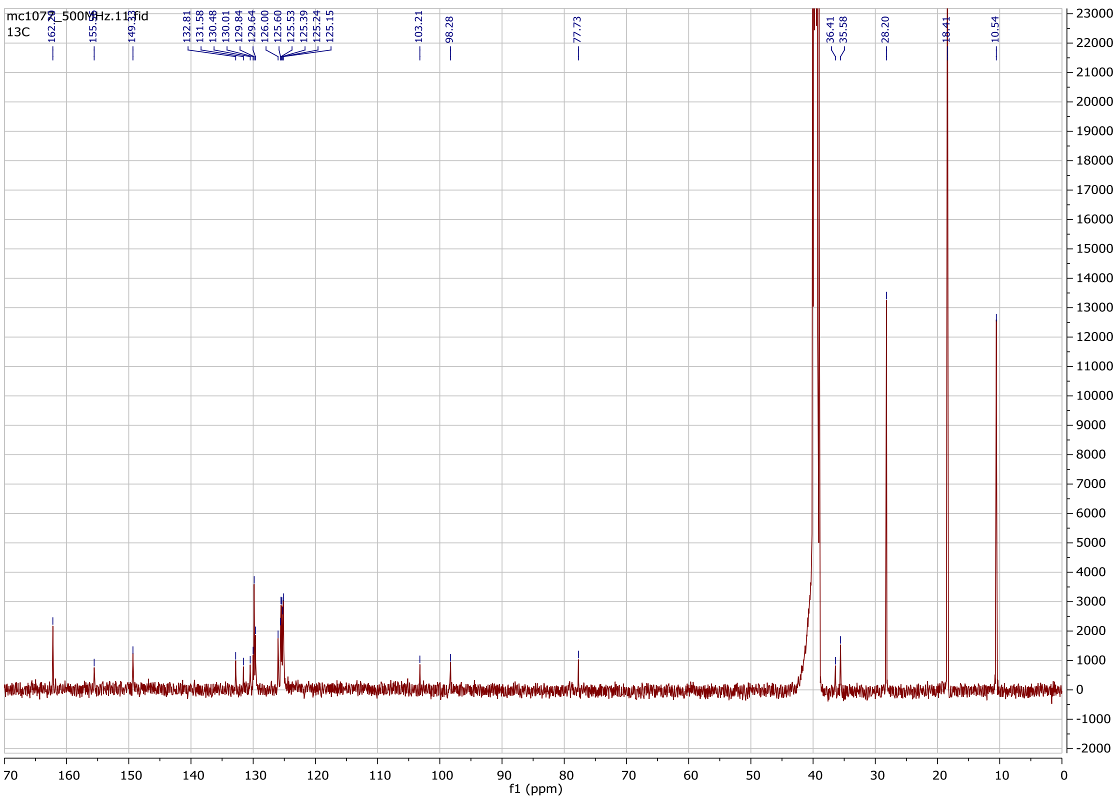

mc1077_500MHz.11.fid
13C
162.7
155.9
149.33
132.81
131.58
130.48
130.01
129.84
129.64
126.00
125.60
125.53
125.39
125.24
125.15
103.21
98.28
77.73
36.41
35.58
28.20
18.41
10.54
f1 (ppm)

Synthesis of **Tetramer-NHBoc**

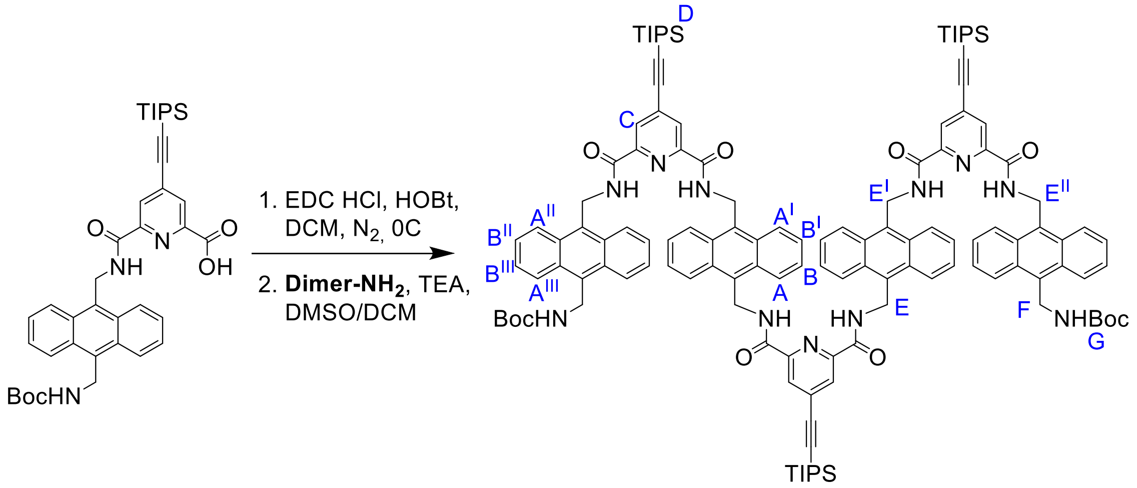

99 mg of **6** (0.1482 mmol) and 20 mg of HOBt•H_2O (0.1482 mmol) were vacuum dried for 2 hours and then suspended in 3 ml of dry DCM under N_2 at 0ºC. 29 mg of EDC•HCl (0.1482 mmol) were added and the mixture was stirred at 0ºC until it changed into a clear solution (45 min). 50.0 mg of **Dimer-NH$_2$** trifluoroacetate (0.0494 mmol) and 34 µl of TEA (0.2470 mmol) were dissolved in 750 µl of DMSO and 1 ml of dry DCM under N_2 and added to the reaction. The mixture was stirred overnight at r.t. with protection from light. The reaction was diluted with $CHCl_3$ and washed with water + HCl and BRINE and the product was purified by flash chromatography in $CHCl_3$. Product was finally precipitated from DCM to Pentane.
75.5 mg (0.0363 mmol) obtained, pale yellow solid. 73%

^{1}H NMR (500 MHz, DMSO-d_6, 35ºC) δ 9.36 (broad m, 6H, NH amide), 8.41 (m, 16H, *A*), 8.14 – 8.01 (m, 6H, C), 7.51 – 7.41 (m, 4H, *B*), 7.34 – 7.16 (m, 14H, B, NH Boc), 5.42 (s, 8H, *E*), 5.35 (s, 4H, F), 5.11 (d, J = 5.5 Hz, 4H, E), 1.36 (s, 18H, G), 1.10 (m, 63H, D); ^{13}C NMR (126 MHz, DMSO-d_6, 35ºC) δ 162.11, 155.49, 149.26, 132.76, 129.79, 129.66, 129.55, 125.93, 125.43, 125.26, 125.12, 124.95, 103.13, 98.16, 77.67, 36.70, 35.61, 28.12, 18.31, 18.27, 10.47; MS (MALDI): m/z calcd. for $C_{128}H_{143}N_{11}O_{10}Si_3Na$ $[M+Na]^+$: 2101.022, found 2101.489; UV-Vis (TCE 10^{-5}, 25ºC) λ max: 341.0, 358.0, 377.0, 398.0 nm; Fluorescence (TCE, 25ºC) A_{356nm}=0.1, λ max: 407.5, 429.5, 458.5, 488.5 nm.

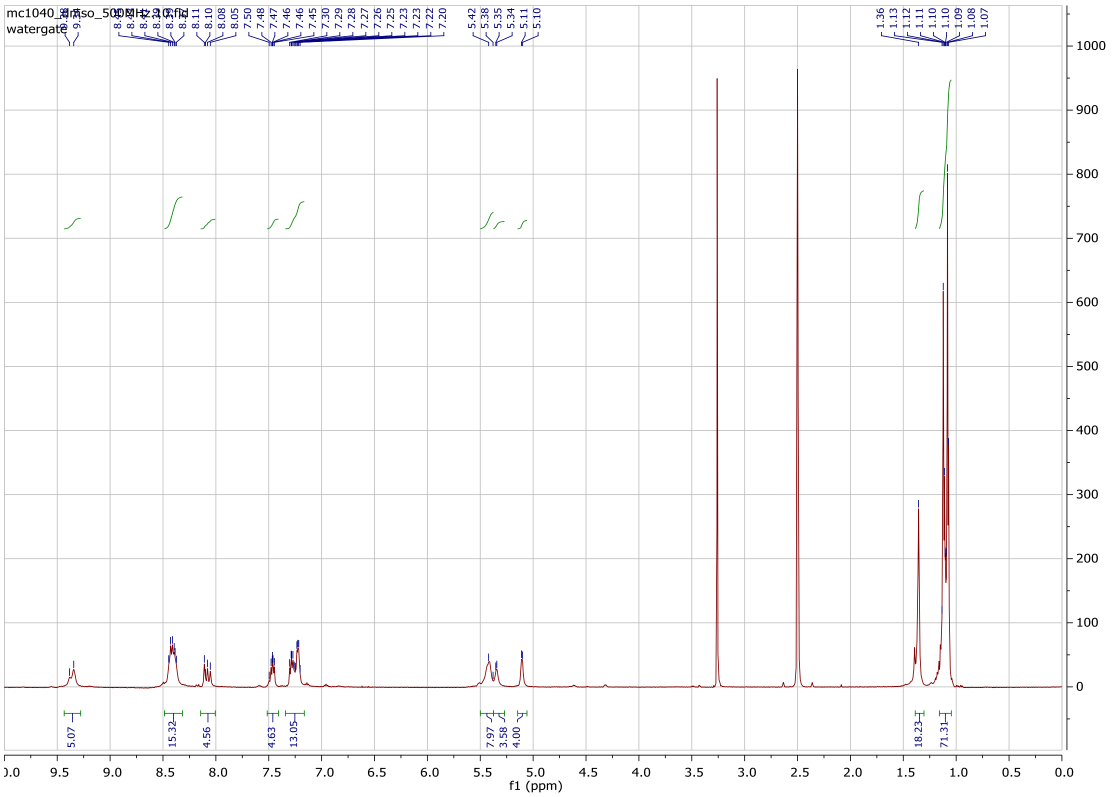
mc1040_dmso_500MHz.10.fid
watergate
f1 (ppm)

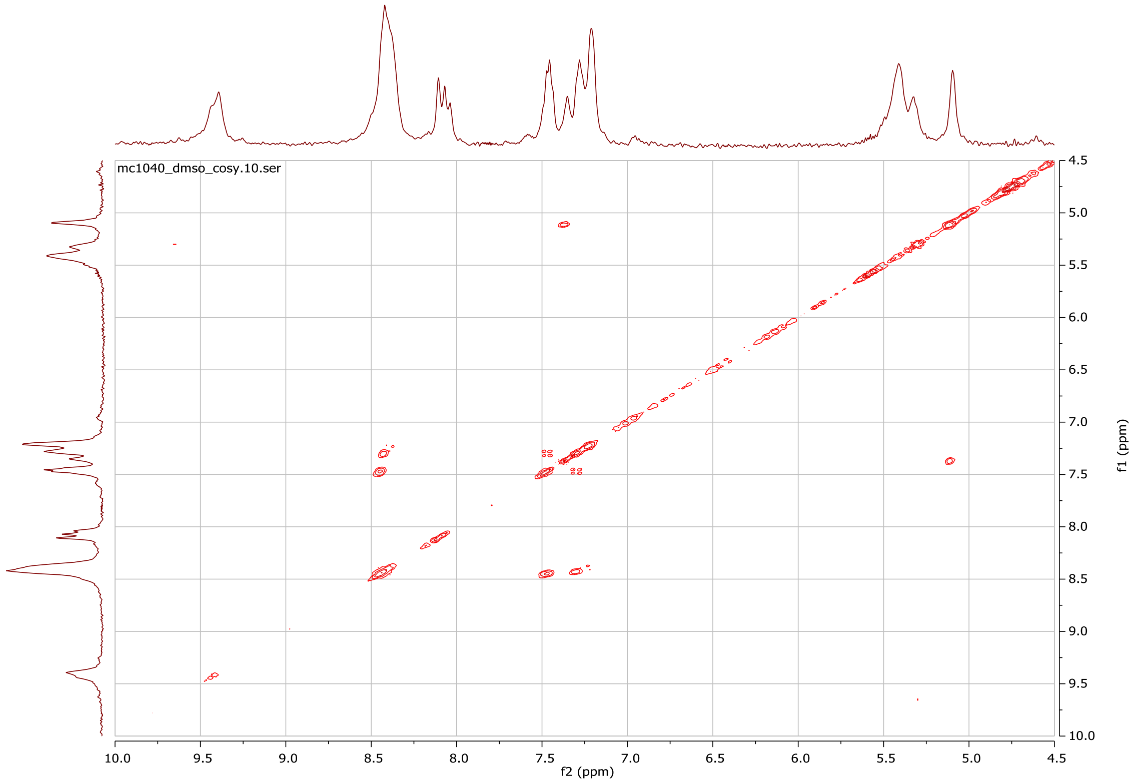
mc1040_dmso_cosy.10.ser
f2 (ppm)
f1 (ppm)

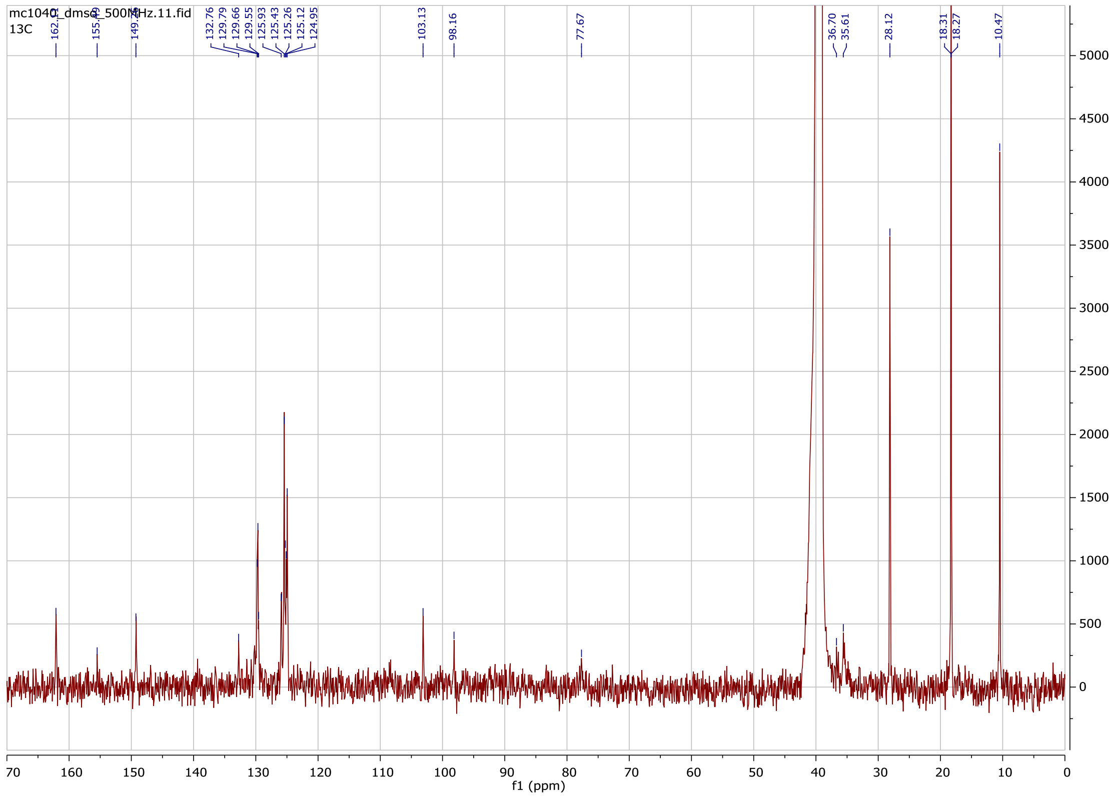
mc1040_dmso_500MHz.11.fid
13C
f1 (ppm)

Synthesis of **Pentamer-NHBoc**

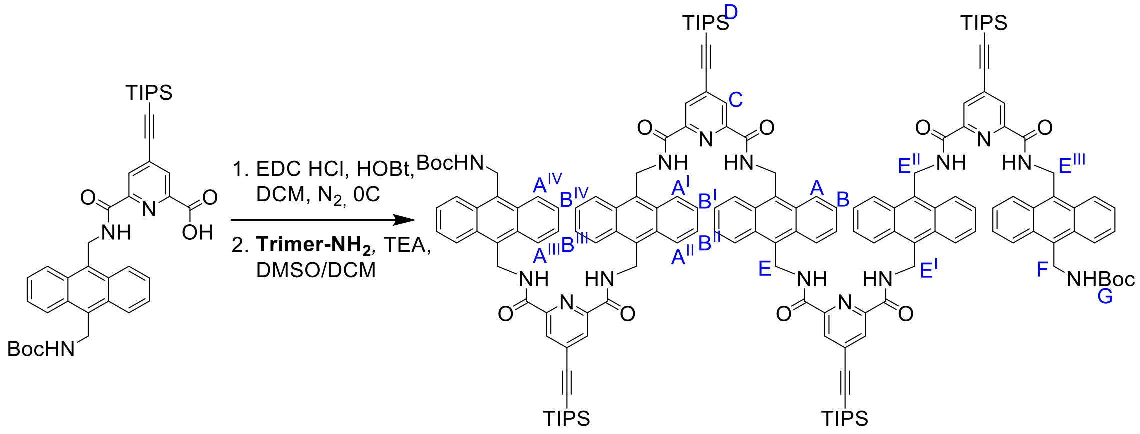

64.0 mg of **6** (0.962 mmol) and 13.0 mg of HOBt•H_2O (0.0962 mmol) were vacuum dried for 30 minutes and then suspended in 3 ml of dry DCM under N_2 at 0ºC. 18.4 mg of EDC•HCl (0.0962 mmol) were added and the mixture was stirred at 0ºC until it changed into a clear solution (45 min). 50.0 mg of **Trimer-NH$_2$** trifluoroacetate (0.0320 mmol) and 22 µl of TEA (0.1603) were dissolved in 500 µl of DMSO and 1 ml of dry DCM under N_2 and added to the reaction. The mixture was stirred overnight at r.t. with protection from light. The reaction was diluted with $CHCl_3$ and washed with water and BRINE and the product was purified by flash chromatography in $CHCl_3$. Product was finally precipitated from DCM to Pentane

63.2 mg (0.0240 mmol) obtained, pale yellow solid. 75%

^{1}H NMR (500 MHz, DMSO-d_6, 60ºC) δ 9.23 (broad m, 8H, NH amide), 8.50 – 8.22 (m, 20H, *A*), 8.17 – 7.93 (m, 8H, C), 7.45 (m, 6H, *B*), 7.33 – 7.08 (m, 14H, *B*), 7.09 – 6.93 (m, 2H, NH Boc), 5.54 – 5.20 (m, 16H, *E*), 5.12 (d, J = 5.6 Hz, 4H, F), 1.37 (s, 18H, G), 1.10 (m, 84H, D); ^{13}C NMR (126 MHz, DMSO-d_6, 60ºC) δ 162.13, 161.96, 149.11, 132.57, 132.40, 129.89, 129.48, 125.65, 125.14, 124.99, 124.86, 124.60, 103.03, 98.20, 77.59, 36.48, 35.54, 35.38, 27.95, 18.06, 10.58, 10.36; MS (MALDI): m/z calcd. for $C_{162}H_{180}N_{14}O_{12}Si_4Na$ $[M+Na]^+$: 2649.291, found 2649.270; UV-Vis (TCE 10^{-5}, 25ºC) λ max: 343.0, 360.0, 377.5, 401.5 nm; Fluorescence (TCE, 25ºC) A_{356nm}=0.1, λ max: (sh), 433.5, 460.0, 483.5 nm.

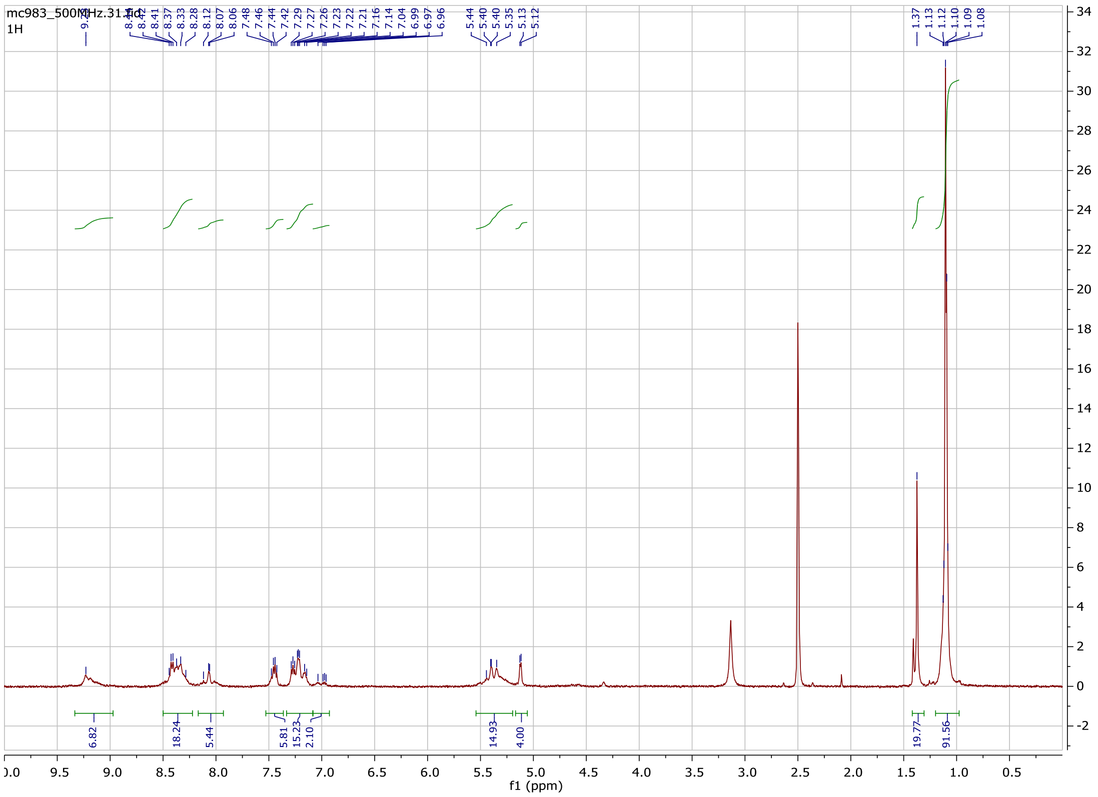
mc983_500MHz.31.fid
1H
f1 (ppm)

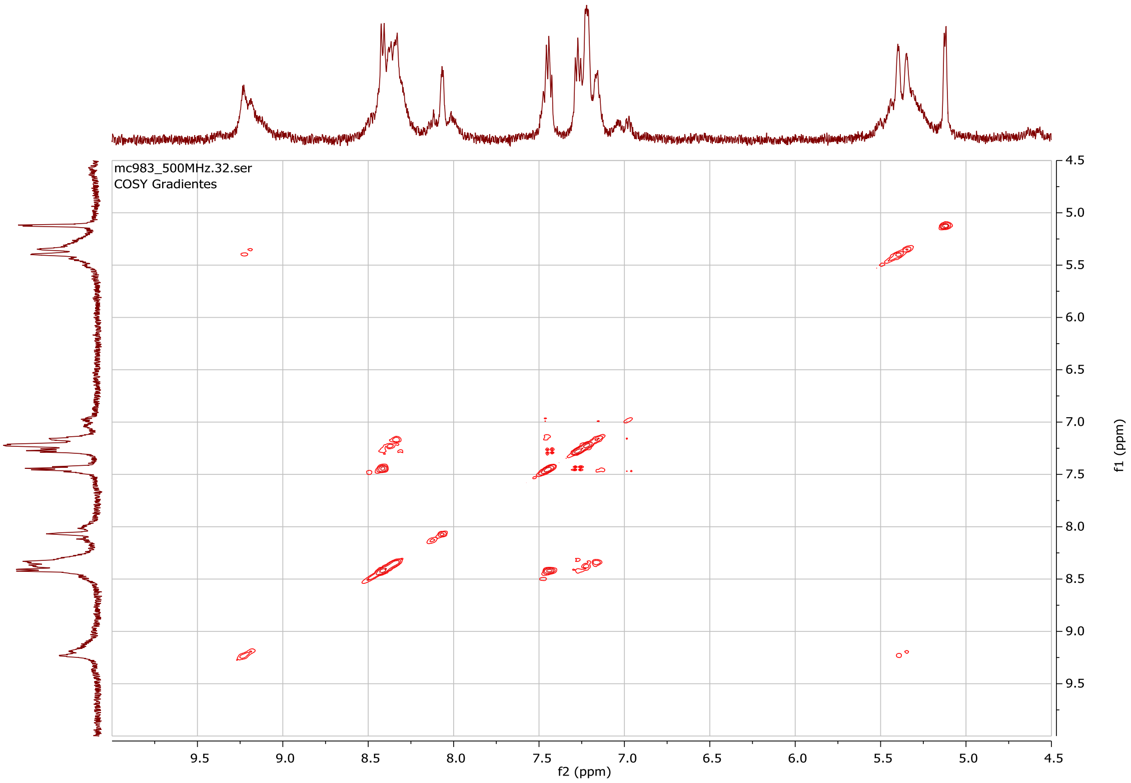
mc983_500MHz.32.ser
COSY Gradientes
f2 (ppm)
f1 (ppm)

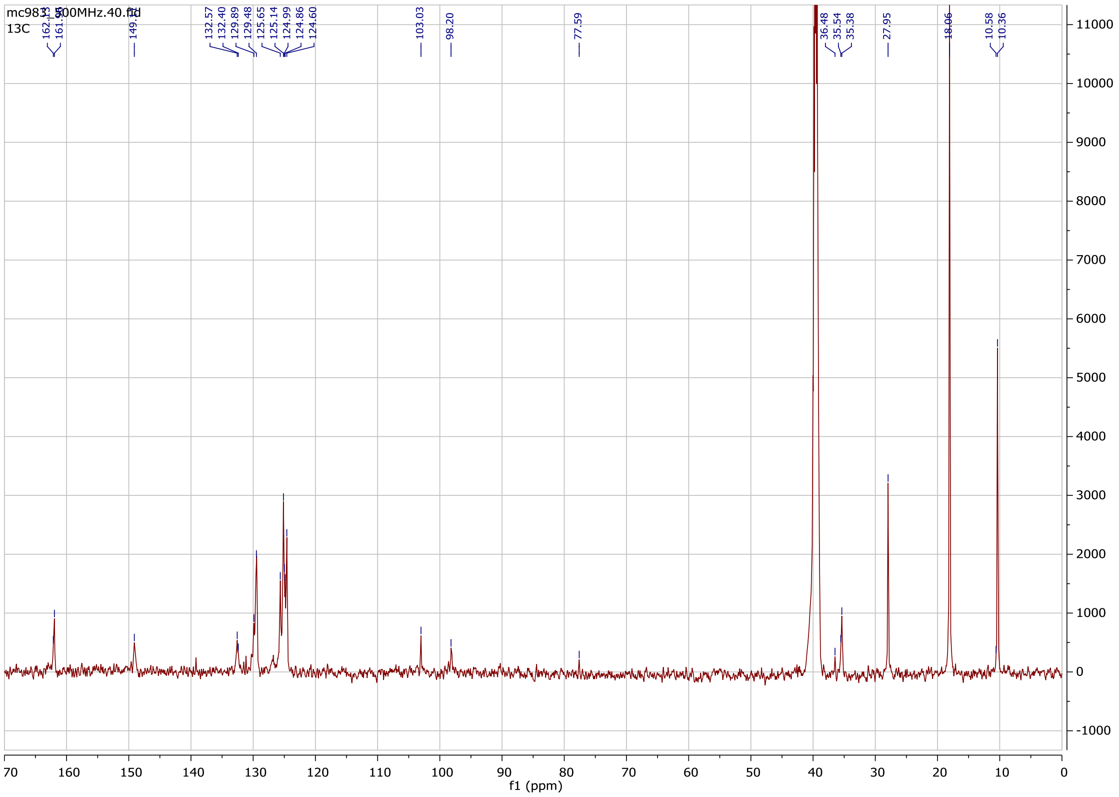
mc983_500MHz.40.fid
13C
132.57
132.40
129.89
129.48
125.65
125.14
124.99
124.86
124.60
103.03
98.20
77.59
36.48
35.54
35.38
27.95
18.06
10.58
10.36
f1 (ppm)

Synthesis of **Dimer-NH_2**

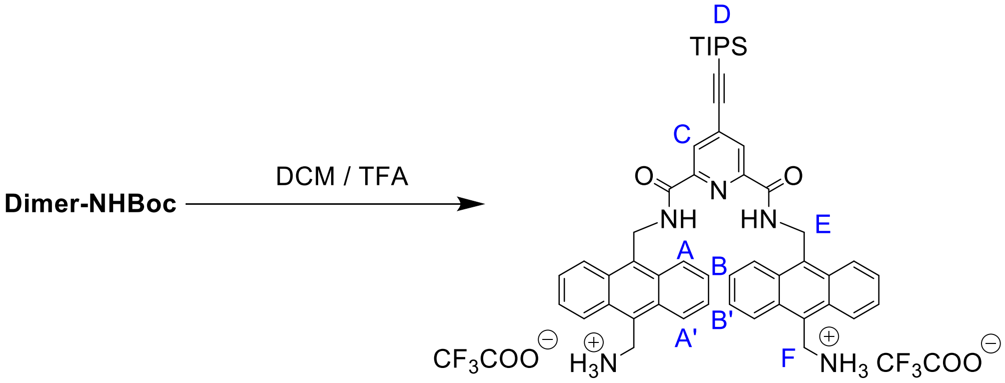

89.5 mg of **Dimer-NHBoc** (0.0909 mmol) were dissolved in 5 ml of DCM and 5 ml of TFA, the mixture was stirred at r.t. for 3 hours, the solvent was evaporated and the product was precipitated from MeOH to Et_2O.
70.3 mg (0.0695 mmol) obtained, pale yellow solid. 76%

^{1}H NMR (400 MHz, DMSO-d_6) δ 9.64 (t, J = 6.3 Hz, 2H, NH amide), 8.65 (d, J = 9.1 Hz, 4H, *A*), 8.46 (d, J = 9.1 Hz, 4H, *A*), 8.31 (s, 6H, NH_3^+), 8.09 (s, 2H, C), 7.69 (t, J = 7.9 Hz, 4H, *B*), 7.44 (t, J = 7.9 Hz, 4H, *B*), 5.56 (d, J = 6.1 Hz, 4H, E), 5.14 (s, 4H, F), 1.10 (m, 21H, D); ^{13}C NMR (101 MHz, DMSO-d_6) δ 162.29, 149.24, 132.90, 132.61, 129.95, 129.82, 126.50, 126.05, 125.94, 125.81, 125.71, 124.53, 103.17, 98.43, 35.70, 34.55, 18.41, 10.53; MS (MALDI): m/z calcd. for $C_{50}H_{53}N_5O_2SiNa$ $[M+Na]^+$: 806.387, found 806.473.

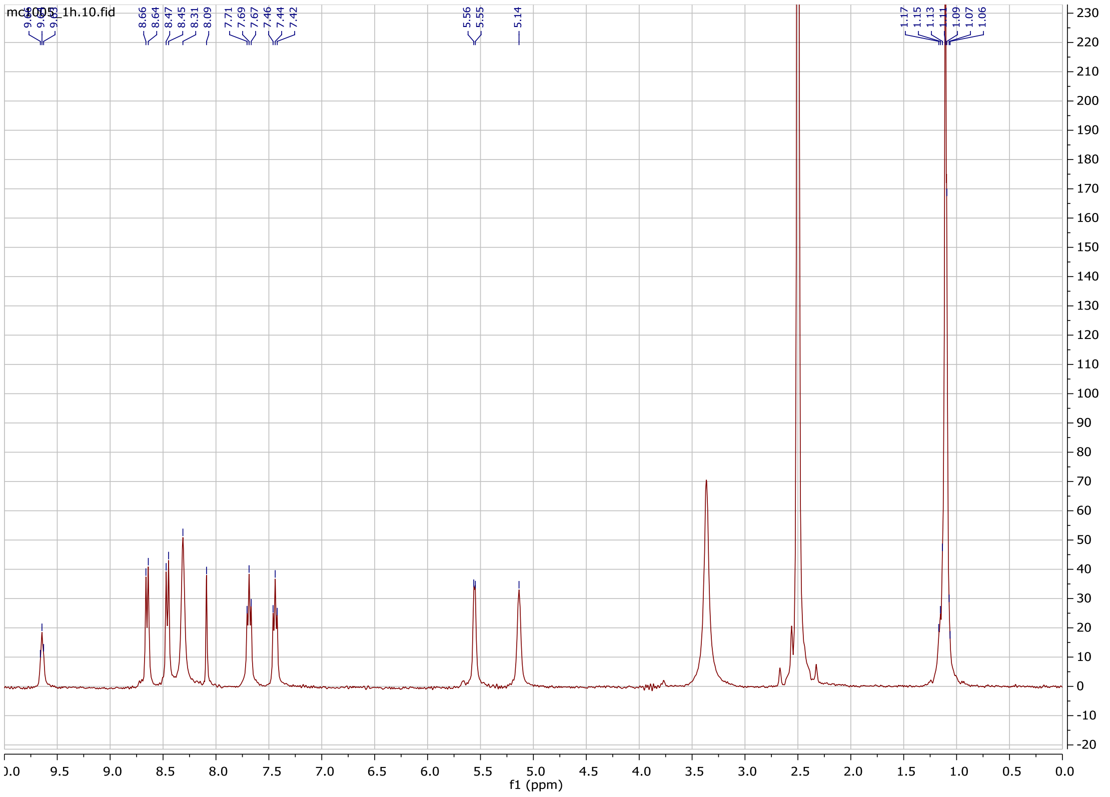

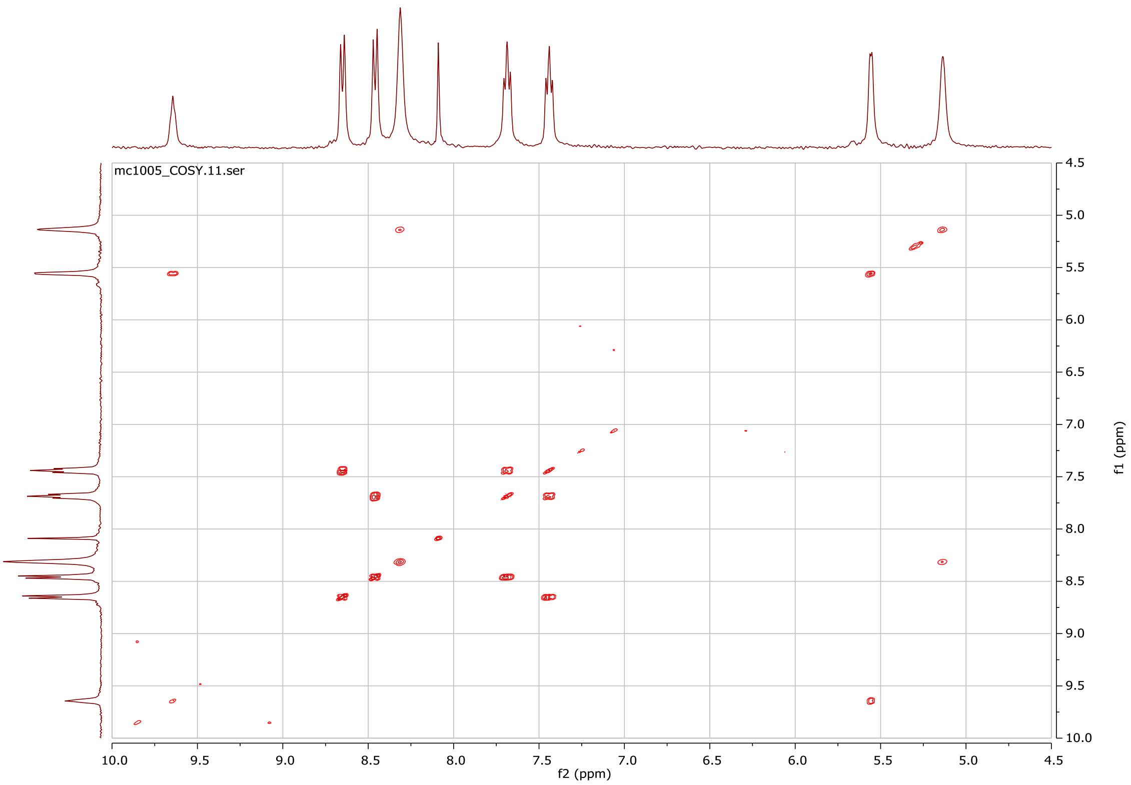
mc1005_COSY.11.ser
f2 (ppm)
f1 (ppm)

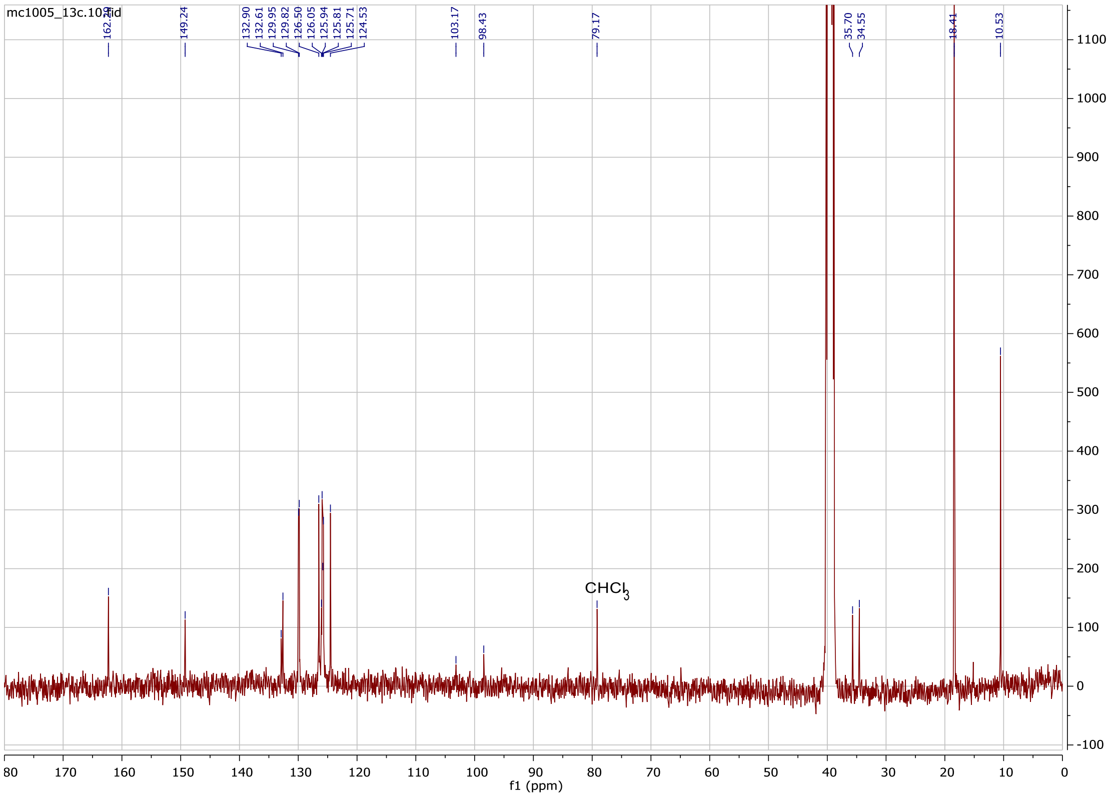
mc1005_13c.10.fid
162.27
149.24
132.90
132.61
129.95
129.82
126.50
126.05
125.94
125.81
125.71
124.53
103.17
98.43
79.17
35.70
34.55
18.41
10.53
CHCl3
f1 (ppm)

Synthesis of **Trimer-NH$_2$**

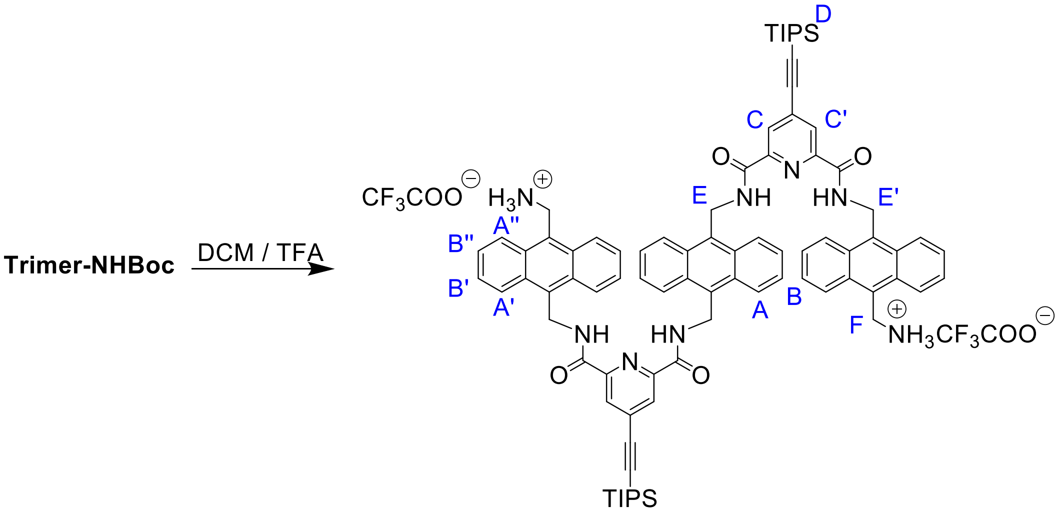

124 mg of **Trimer-NHBoc** (0.0811 mmol) were dissolved in 5 ml of DCM / 5 ml of TFA, the mixture was stirred for 3 hours, then the solvent was evaporated and co-evaporated with Et_2O and MeOH/Et_2O. The product was precipitated from MeOH to Et_2O.

112 mg (0.0715 mmol) obtained, pale yellow powder. 88%

^{1}H NMR (400 MHz, DMSO-d$_6$) δ 9.73 (s, 2H, NH amide), 9.59 (s, 2H, NH amide), 8.68 – 8.49 (m, 8H, *A*), 8.17 (m, 10H, A, NH_3^+), 8.10 (s, 4H, C,C'), 7.48 (m, 8H, *B*), 7.13 (d, J = 8.0 Hz, 4H, *B*), 5.62 (d, J = 6.1 Hz, 4H, E), 5.46 (d, J = 7.0 Hz, 4H, E), 4.77 (s, 4H, F), 1.10 (d, J = 5.6 Hz, 42H, D); ^{13}C NMR (101 MHz, DMSO-d$_6$) δ 162.40, 162.20, 157.93, 157.63, 149.30, 149.23, 132.95, 132.48, 130.66, 130.05, 129.63, 129.58, 126.26, 126.15, 126.05, 125.79, 125.70, 125.61, 125.49, 125.34, 124.17, 103.22, 98.41, 35.69, 35.61, 34.21, 18.41, 10.54; MS (MALDI): m/z calcd. for $C_{84}H_{90}N_8O_4Si_2Na$ [M+Na]$^+$: 1353.652, found 1353.644.

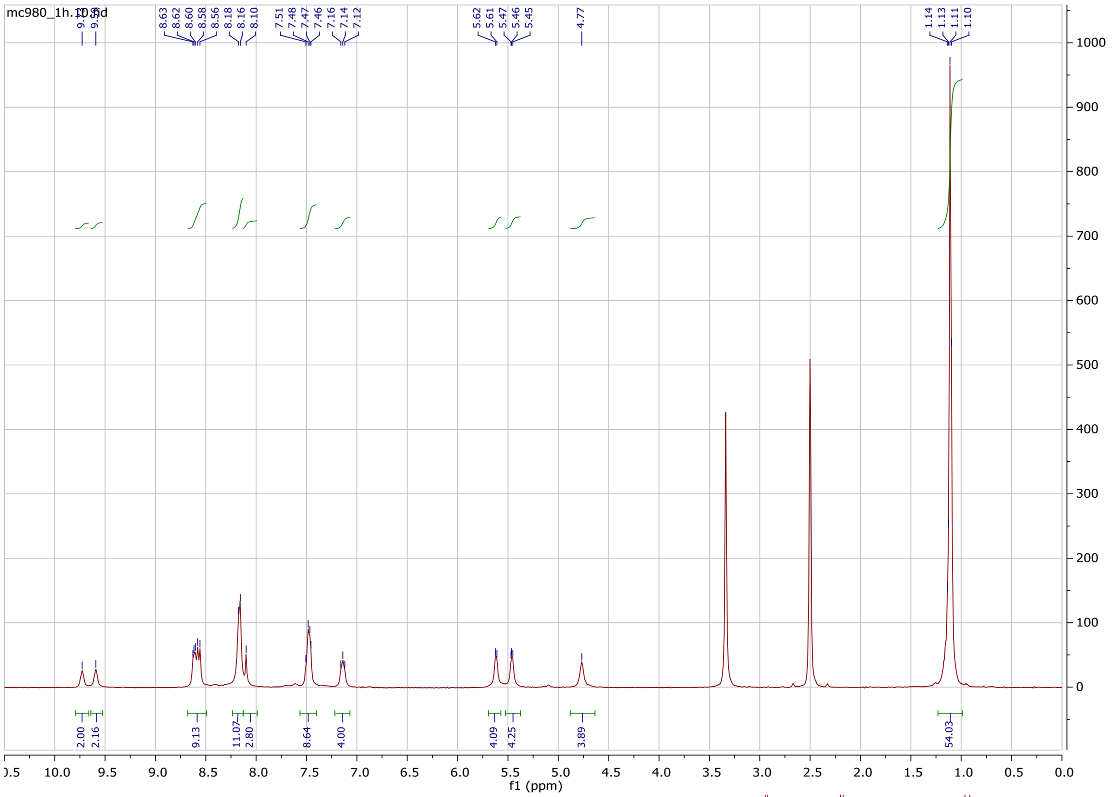
mc980_1h.10.fid
8.63
8.62
8.60
8.58
8.56
8.18
8.16
8.10
7.51
7.48
7.47
7.46
7.16
7.14
7.12
5.62
5.61
5.47
5.46
5.45
4.77
1.14
1.13
1.11
1.10
2.00
2.16
9.13
11.07
2.80
8.64
4.00
4.09
4.25
3.89
54.03
f1 (ppm)

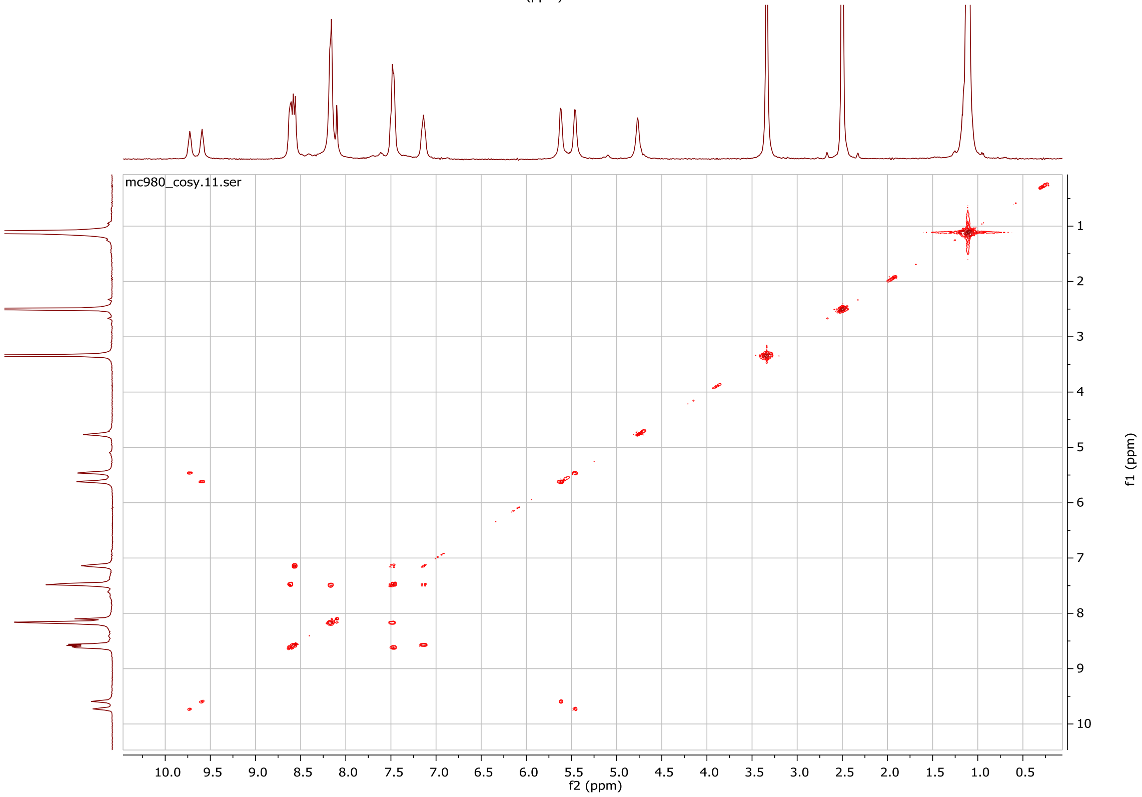
mc980_cosy.11.ser
f2 (ppm)
f1 (ppm)

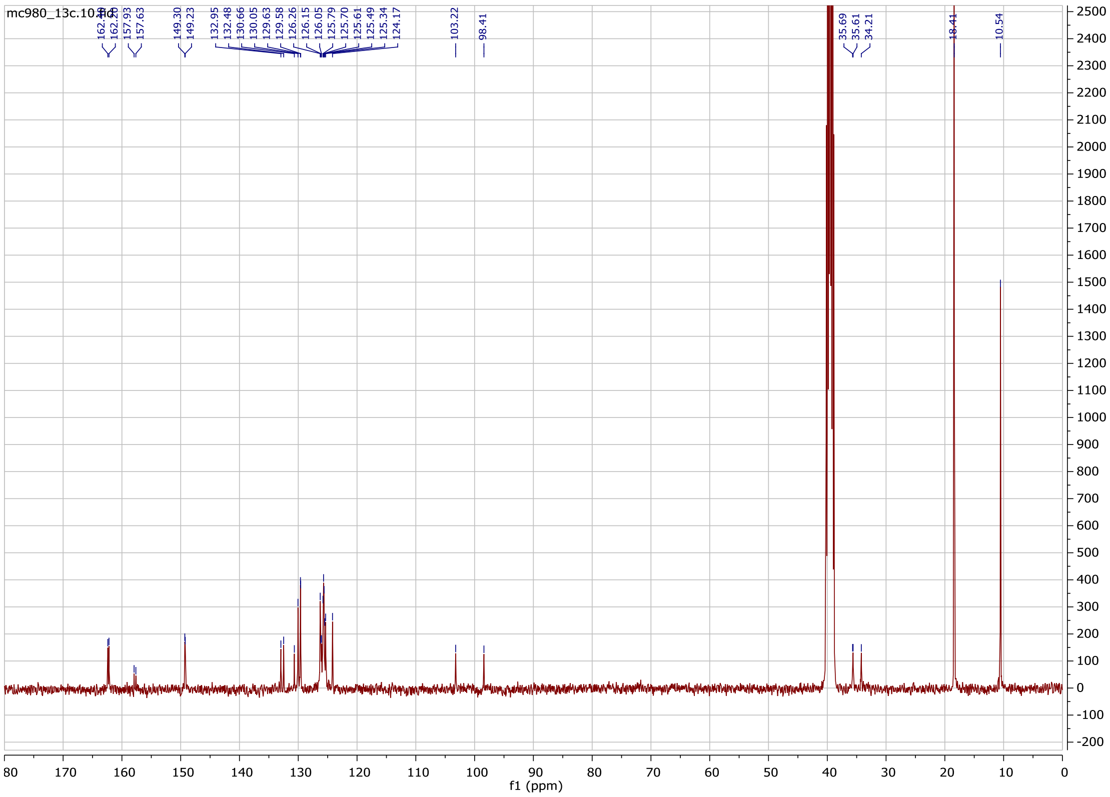
mc980_13c.10.fid
162.28
162.23
157.93
157.63
149.30
149.23
132.95
132.48
130.66
130.05
129.63
129.58
126.26
126.15
126.05
125.79
125.70
125.61
125.49
125.34
124.17
103.22
98.41
35.69
35.61
34.21
18.41
10.54
f1 (ppm)

Synthesis of **Tetramer-NH_2**

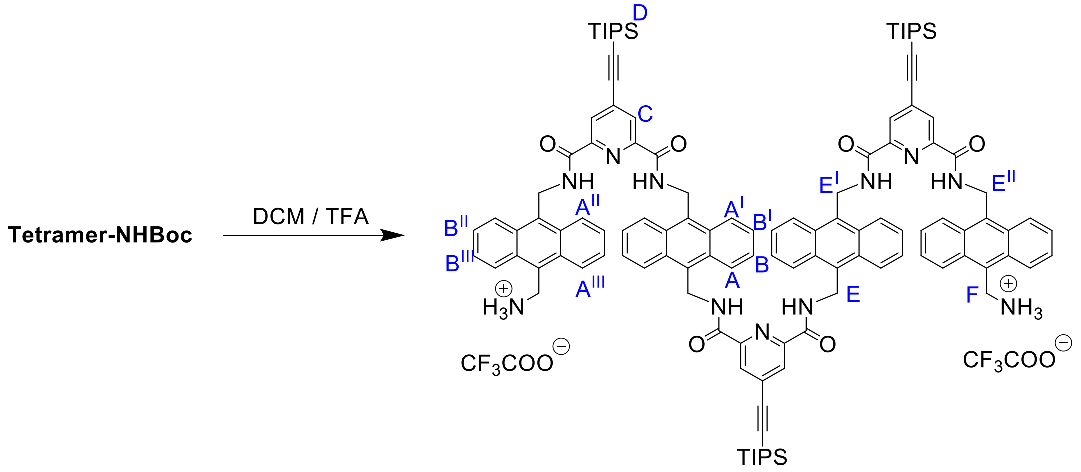

50.0 mg of **Tetramer-NHBoc** (0.0240 mmol) were dissolved in 5 ml of DCM and 5 ml of TFA, the mixture was stirred at r.t. for 3 hours, the solvent was evaporated and the product was precipitated from MeOH to Et_2O.
45.5 mg (0.0216 mmol) obtained, pale yellow solid. 90%

^{1}H NMR (400 MHz, DMSO-d_6) δ 9.74 – 9.26 (m, 4H, NH amide), 8.77 – 7.90 (m, 28H, *A*, C, NH_3^+), 7.61 (m, 8H, *B*), 7.28 (m, 8H, *B*), 5.78 – 5.28 (m, 12H, *E*), 5.28 – 4.85 (m, 4H, F), 1.42 – 0.72 (m, 63H, D); ^{13}C NMR (101 MHz, DMSO-d_6) δ 162.25, 157.93, 149.23, 132.86, 130.41, 129.80, 129.69, 126.35, 126.11, 125.63, 124.36, 103.19, 98.32, 35.67, 34.49, 30.69, 18.42, 18.37, 10.54; MS (MALDI): m/z calcd. for $C_{118}H_{127}N_{11}O_6Si_3Na$ $[M+Na]^+$: 1901.921, found 1901.364.

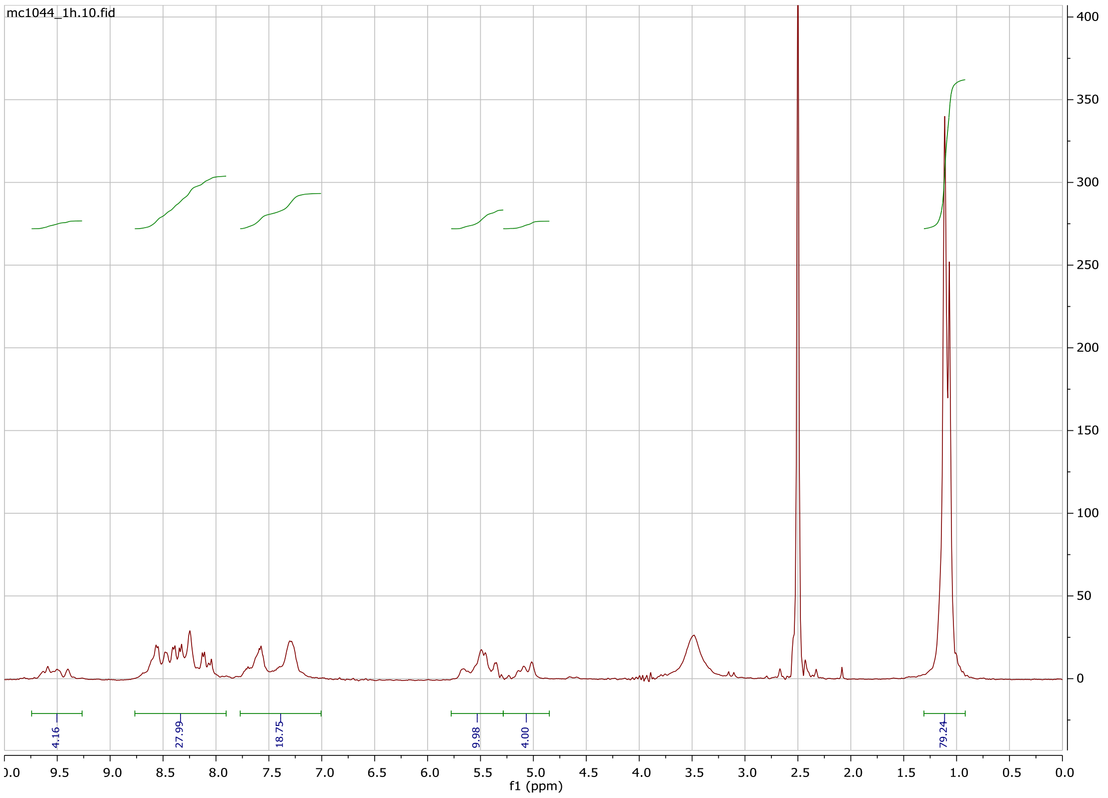

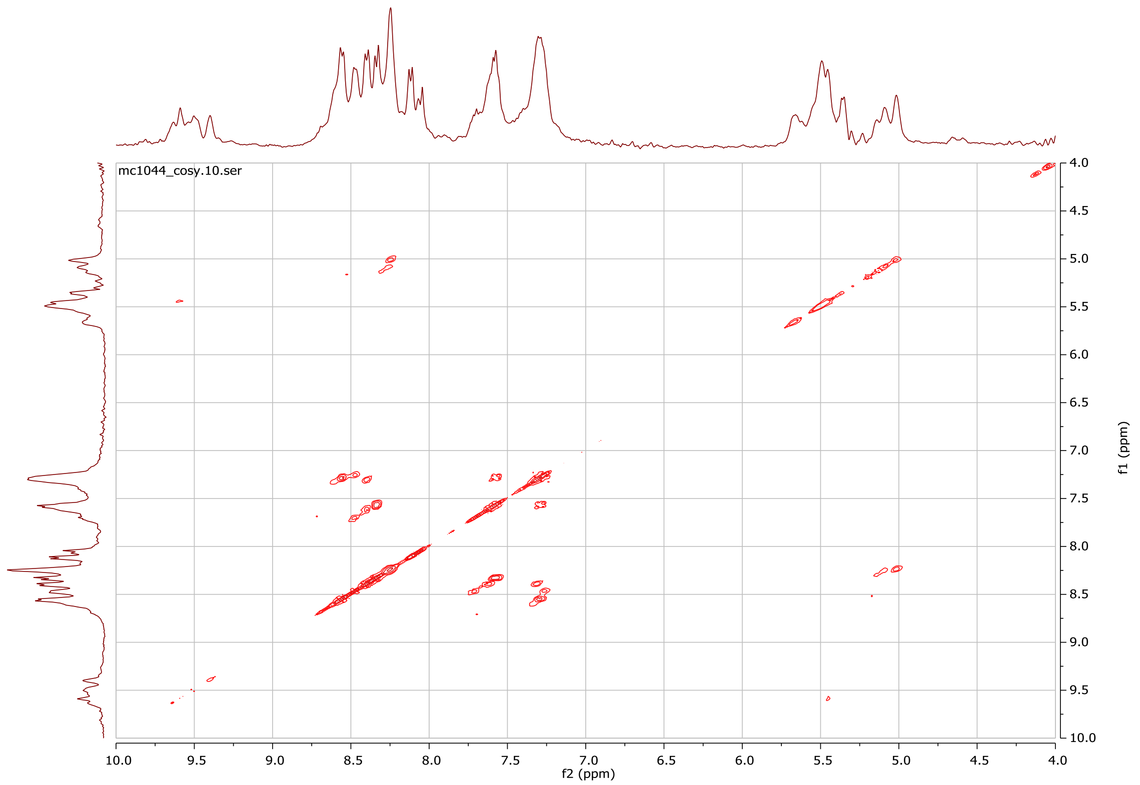
mc1044_cosy.10.ser
f2 (ppm)
f1 (ppm)

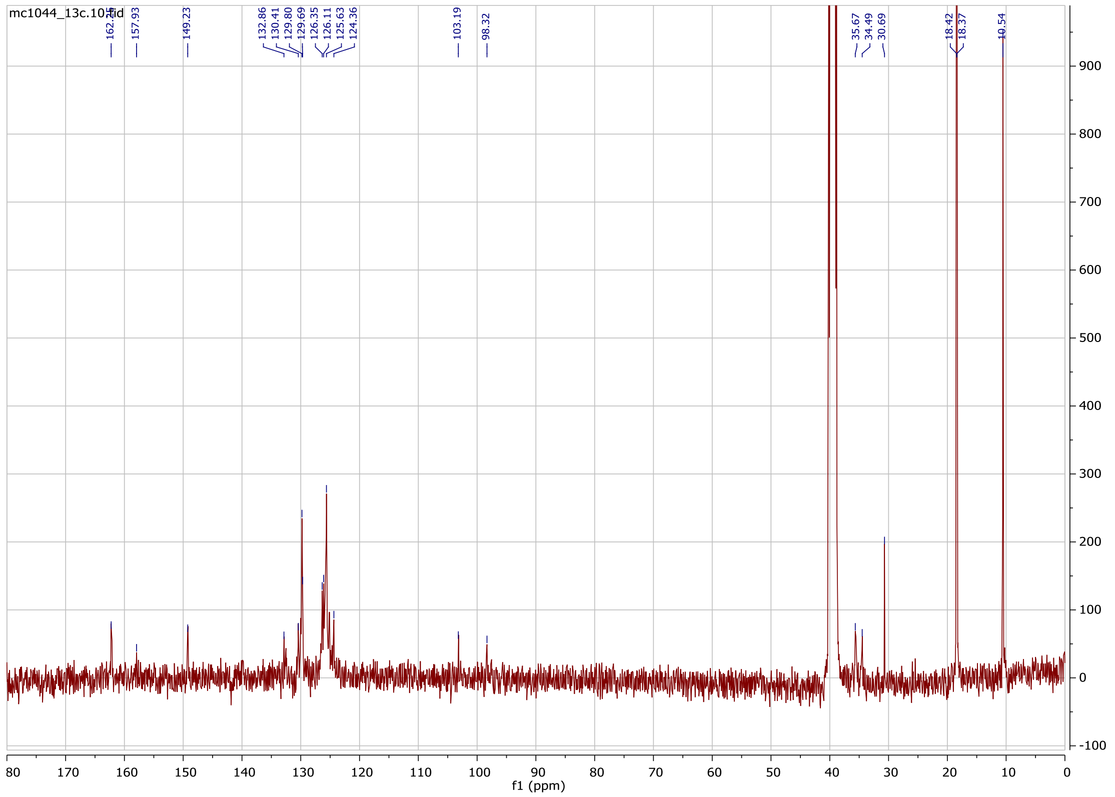
mc1044_13c.10.fid
162.24
157.93
149.23
132.86
130.41
129.80
129.69
126.35
126.11
125.63
124.36
103.19
98.32
35.67
34.49
30.69
18.42
18.37
10.54
f1 (ppm)

Synthesis of **Pentamer-NH2**

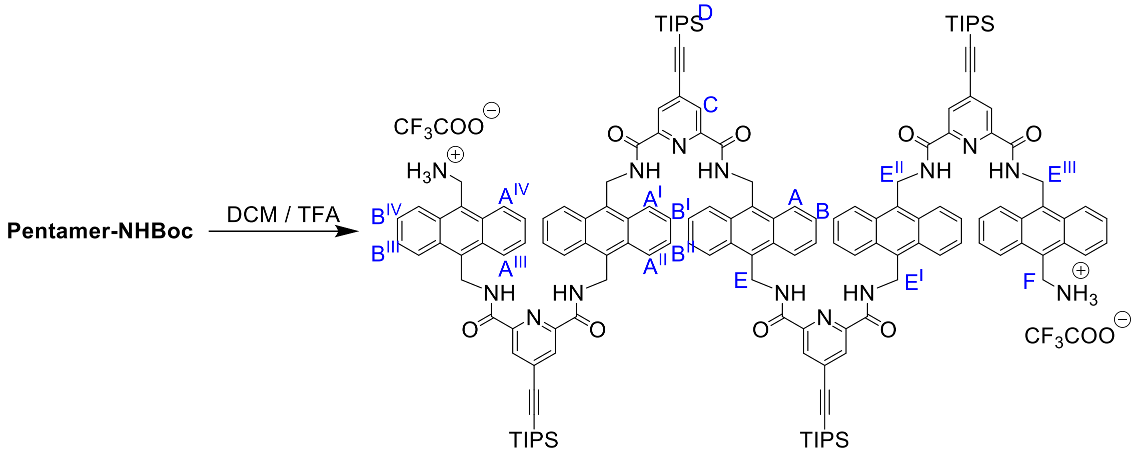

40.0 mg of **Pentamer-NHBoc** (0.0152 mmol) were dissolved in 3 ml of DCM and 3 ml of TFA were added, the mixture was stirred for 3 hours, then the solvent was evaporated and co-evaporated with Et_2O, MeOH/Et_2O, and Hexane. The product was triturated with Et_2O.
37.9 mg (0.0143 mmol) obtained, pale yellow solid. 94%

^{1}H NMR (500 MHz, DMSO-d$_6$, 50ºC) δ 9.45 – 9.10 (m, 8H, NH amide), 8.41 (m, 20H, *A*), 8.21 (s, 6H, NH_3^+), 8.07 (m, 8H, C), 7.57 (dd, J = 9.0, 6.3 Hz, 4H, *B*), 7.39 – 7.22 (m, 12H, *B*), 7.22 – 7.07 (m, 4H, *B*), 5.58 – 5.24 (m, 16H, *E*), 5.03 (s, 4H, F), 1.30 – 0.89 (m, 84H, D); ^{13}C NMR (126 MHz, DMSO-d$_6$, 50ºC) δ 162.13, 162.09, 162.00, 149.15, 132.64, 132.20, 130.02, 129.62, 129.58, 129.46, 126.05, 125.68, 125.46, 125.22, 125.12, 124.77, 124.68, 124.61, 123.93, 102.98, 98.29, 98.23, 35.51, 34.41, 18.06, 10.35, 10.12; MS (MALDI): m/z calcd. for $C_{152}H_{164}N_{14}O_8Si_4Na$ [M+Na]$^+$: 2449.186, found 2449.155.

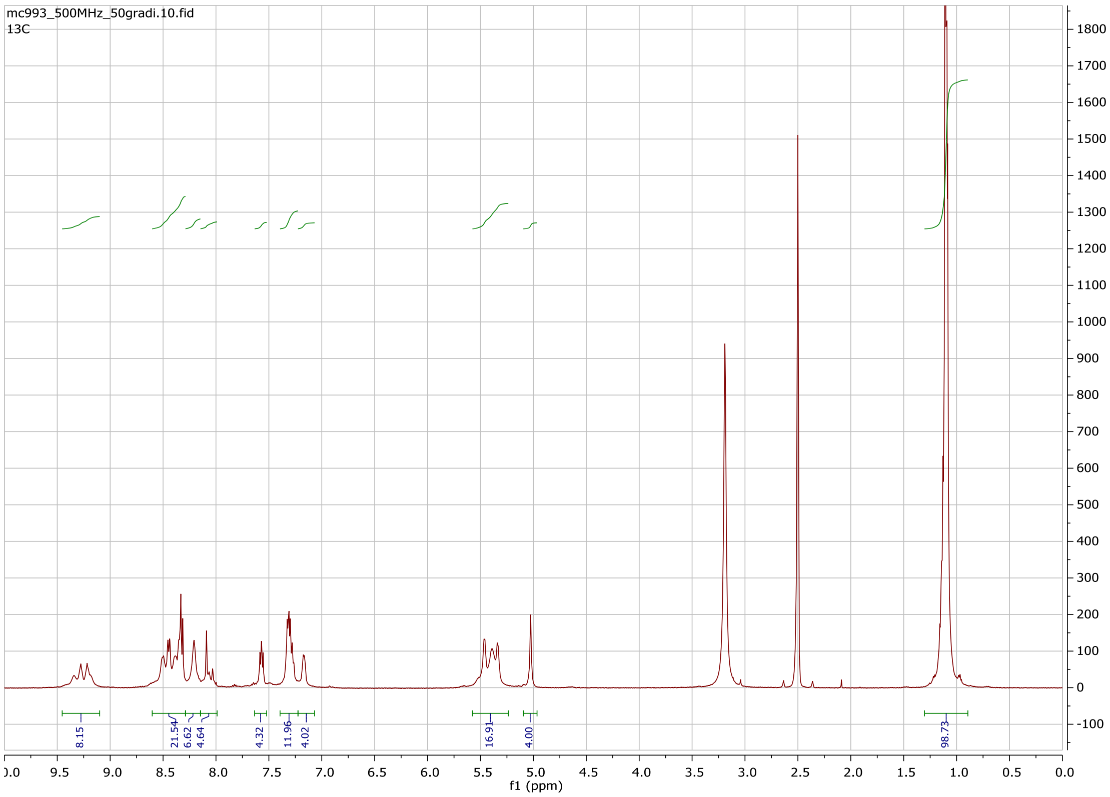
mc993_500MHz_50gradi.10.fid
13C
8.15
21.54
6.62
4.64
4.32
11.96
4.02
16.91
4.00
98.73
f1 (ppm)

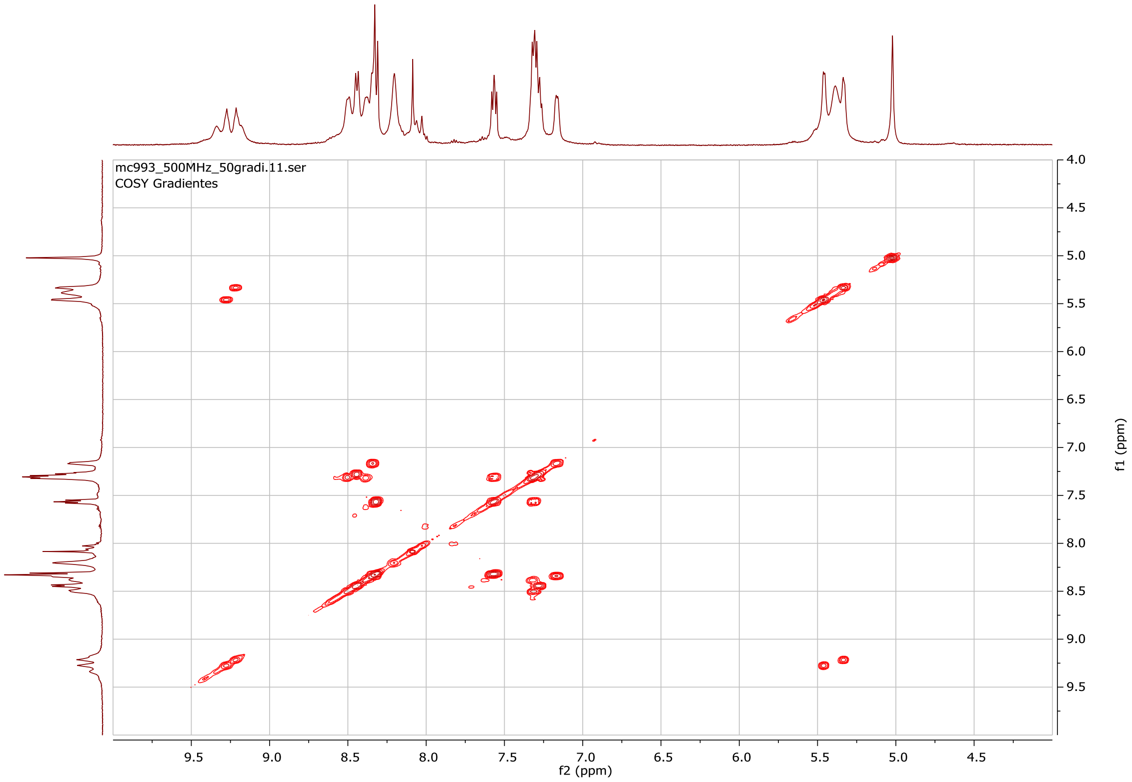
mc993_500MHz_50gradi.11.ser
COSY Gradientes
f2 (ppm)
f1 (ppm)

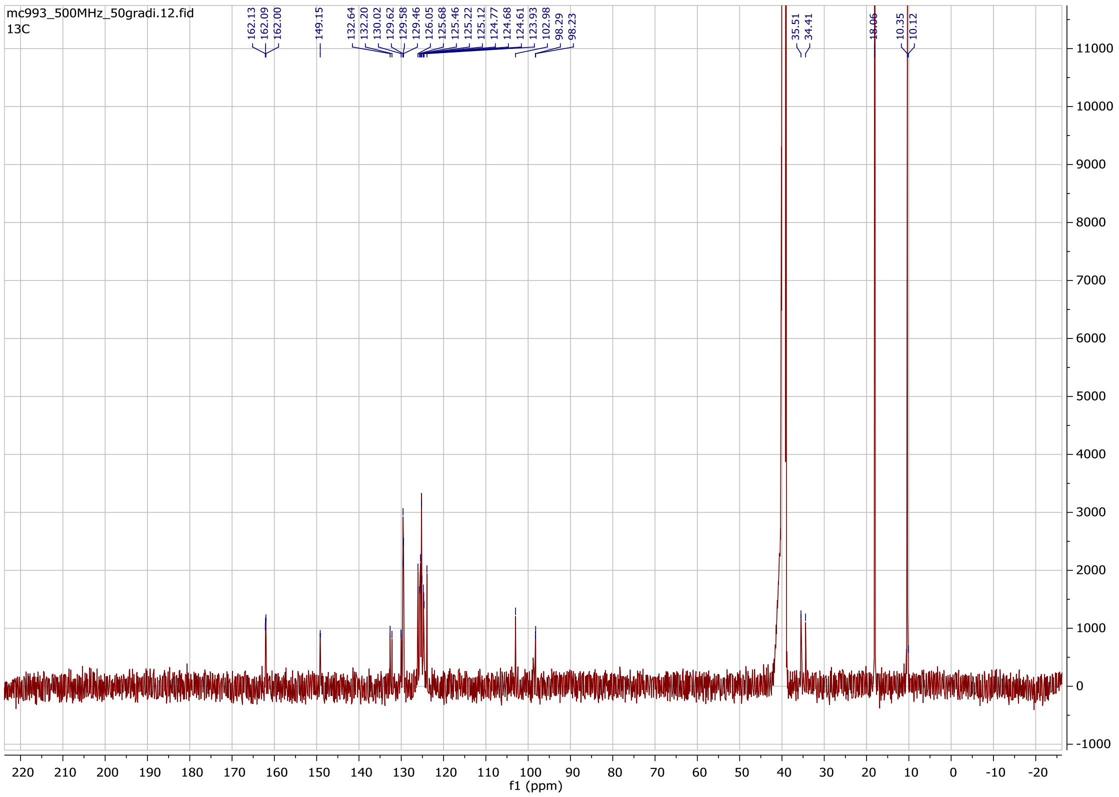
mc993_500MHz_50gradi.12.fid
13C
162.13
162.09
162.00
149.15
132.64
132.20
130.02
129.62
129.58
129.46
126.05
125.68
125.46
125.22
125.12
124.77
124.68
124.61
123.93
102.98
98.29
98.23
35.51
34.41
18.06
10.35
10.12
f1 (ppm)

Synthesis of **#-Trimer-NHBoc**

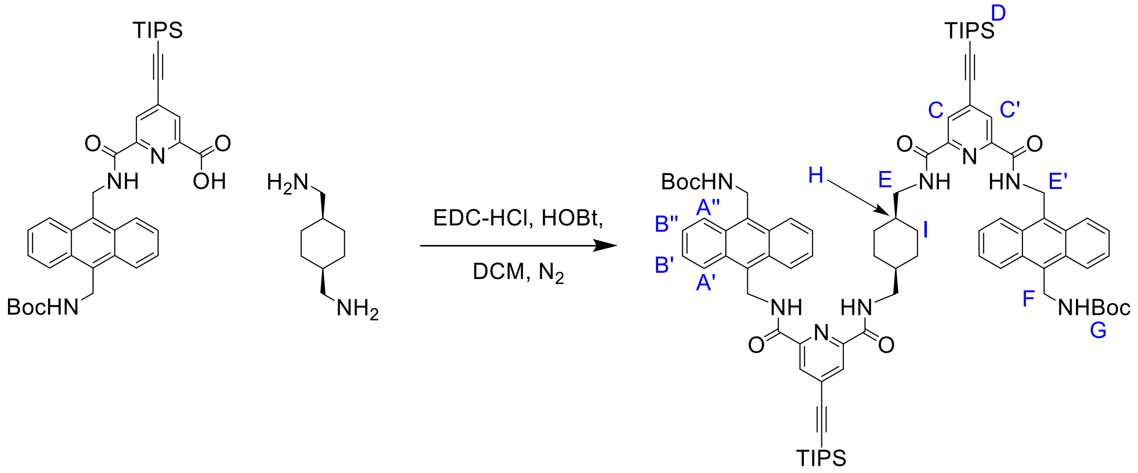

234 mg of **6** (0.351 mmol) and 47 mg of HOBt•H_2O (0.351 mmol) were vacuum dried together and suspended in 5 ml of dry DCM under nitrogen atmosphere. The mixture was cooled to 0ºC and 67 mg of EDC•HCl (0.351 mmol) were added. The reaction was stirred at 0ºC until it became a clear solution (45 min), then 21 μl of cis-1,4-bis(aminomethyl)cyclohexane (0.141 mmol) were added and the reaction was stirred at r.t. overnight with protection from light. The product was purified by chromatography in $CHCl_3$ and precipitation from DCM / Hexane.

108 mg (0.075 mmol) obtained, pale yellow solid. 53%

^{1}H NMR (500 MHz, DMSO-d_6, 70ºC) δ 9.36 (t, J = 5.8 Hz, 2H, NH amide$^{E'}$), 8.87 (t, J = 6.2 Hz, 2H, NH amide^{E}), 8.67 – 8.61 (m, 4H, *A*), 8.52 – 8.46 (m, 4H, *A*), 8.14 (d, J = 1.6 Hz, 2H, C), 8.03 (d, J = 1.6 Hz, 2H, C), 7.60 – 7.48 (m, 8H, B,B'), 7.00 (s, 2H, NH Boc), 5.61 (d, J = 5.6 Hz, 4H, E'), 5.17 (d, J = 5.5 Hz, 4H, F), 3.19 (t, J = 6.8 Hz, 4H, E), 1.61 (m, 2H, H), 1.39 (s, 18H, G), 1.28 (m, 8H, I), 1.21 – 1.06 (m, 42H, D); ^{13}C NMR (126 MHz, DMSO-d_6, 70ºC) δ 162.18, 162.16, 155.12, 149.59, 149.13, 132.60, 131.24, 129.76, 129.70, 129.52, 125.38, 125.25, 125.13, 125.02, 124.92, 124.76, 103.04, 98.10, 77.55, 42.20, 36.44, 35.83, 34.87, 27.88, 25.65, 17.98, 10.30; MS (MALDI): m/z calcd. for $C_{86}H_{108}N_8O_8Si_2Na$ $[M+Na]^+$: 1459.776, found 1460.243; UV-Vis (TCE 10^{-5}, 25ºC) λ max: 340.5, 357.0, 376.0, 397.0 nm; Fluorescence (TCE, 25ºC) A_{356nm}=0.1, λ max: 406.0, 427.0, 451.0, (sh) nm.

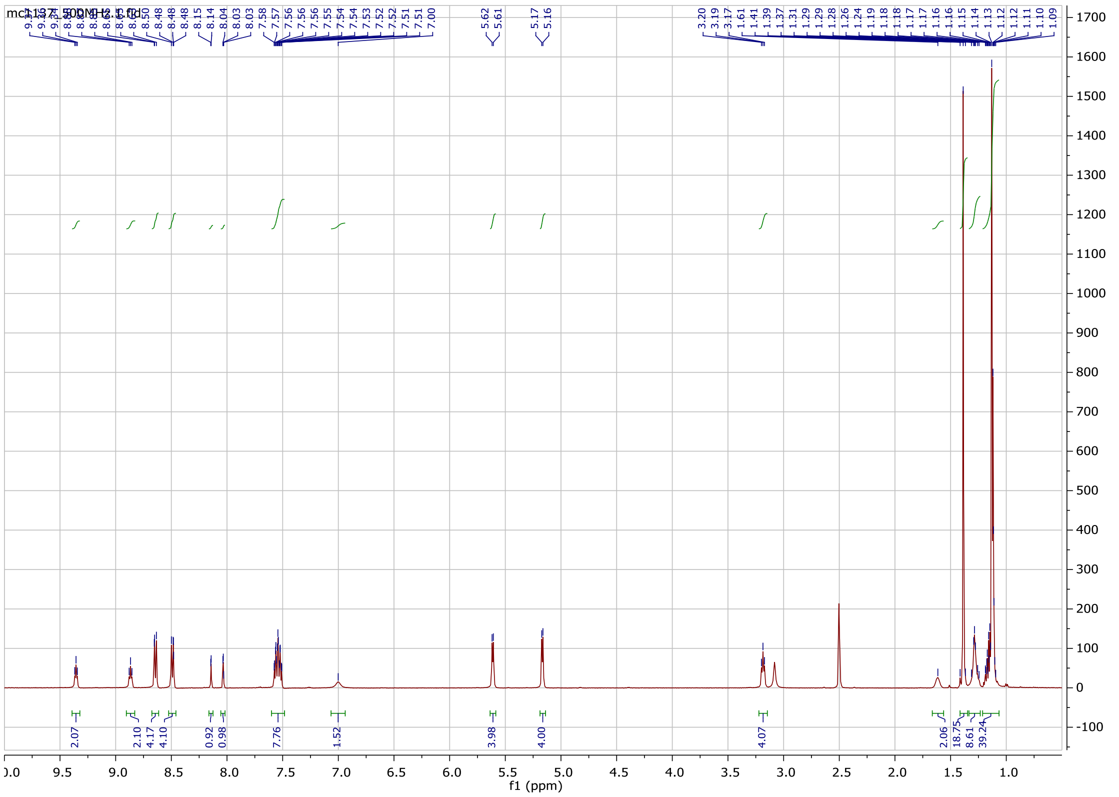

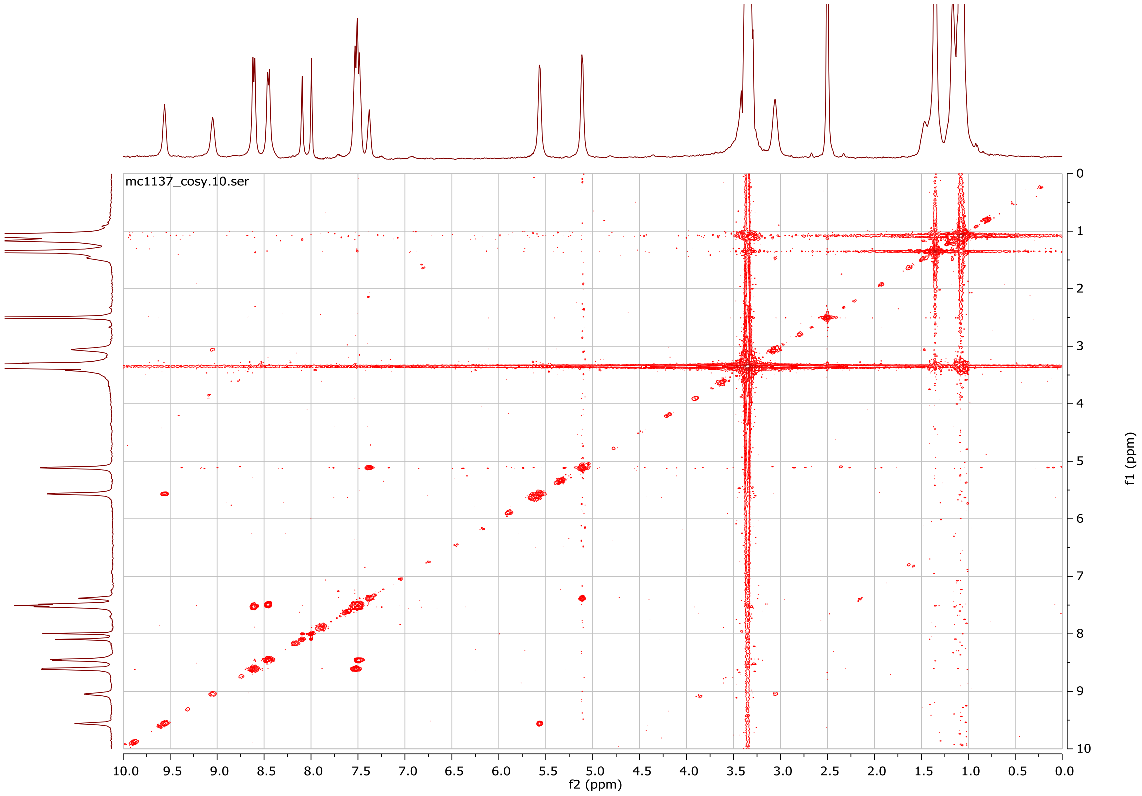

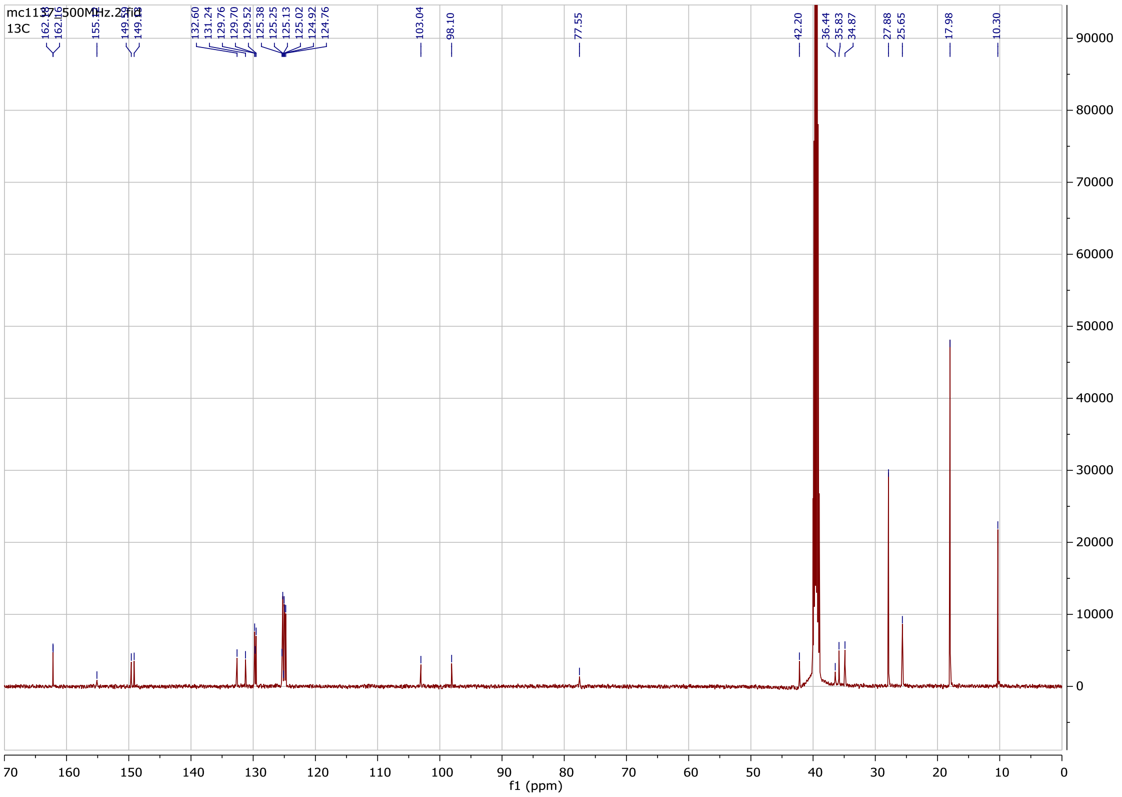
mc1137-500MHz.2.fid
13C
155.1
132.60
131.24
129.76
129.70
129.52
125.38
125.25
125.13
125.02
124.92
124.76
103.04
98.10
77.55
42.20
36.44
35.83
34.87
27.88
25.65
17.98
10.30
f1 (ppm)

Synthesis of **#-Trimer-NH$_2$**

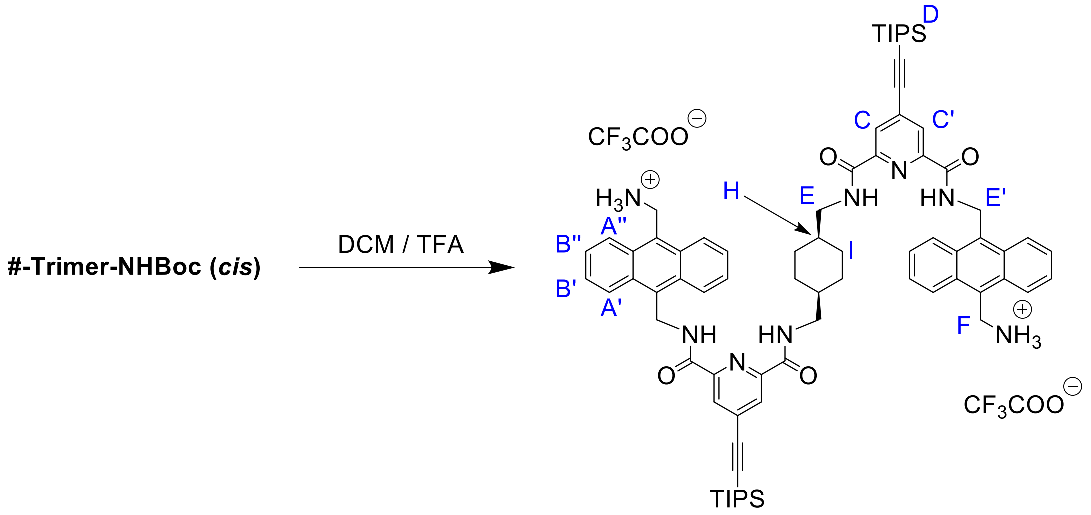

66.7 mg of **#-Trimer-NHBoc** (0.0464 mmol) were dissolved in 3 ml of DCM and 3 ml of TFA. The mixture was stirred for 3 hours and the solvents were evaporated. The product was precipitated from MeOH / Et$_2$O.
64.2 mg (0.0438 mmol) obtained, pale yellow solid. 94%

^{1}H NMR (400 MHz, DMSO-d$_6$) δ 9.73 (t, J = 6.0 Hz, 2H, NH amide$^{E'}$), 9.21 (t, J = 6.5 Hz, 2H, NH amide^E), 8.87 – 8.69 (m, 4H, *A*), 8.49 – 8.38 (m, 4H, *A*), 8.31 (s, 6H, NH_3^+), 8.11 (s, 2H, C), 8.03 (s, 2H, C), 7.76 – 7.49 (m, 8H, B,B'), 5.74 – 5.56 (m, 4H, E'), 5.09 (s, 4H, F), 3.23 (m, 4H, E), 1.63 (m, 2H, H), 1.32 (m, 8H, I), 1.07 (m, 42H, D); ^{13}C NMR (101 MHz, DMSO-d$_6$) δ 162.49, 162.42, 149.66, 149.16, 132.92, 132.67, 130.01, 129.93, 126.48, 126.01, 125.85, 125.79, 124.59, 103.25, 98.37, 42.41, 36.12, 35.39, 34.59, 25.98, 18.40, 10.54; MS (MALDI): m/z calcd. for $C_{76}H_{92}N_8O_4Si_2Na$ [M+Na]$^+$: 1259.668, found 1260.087.

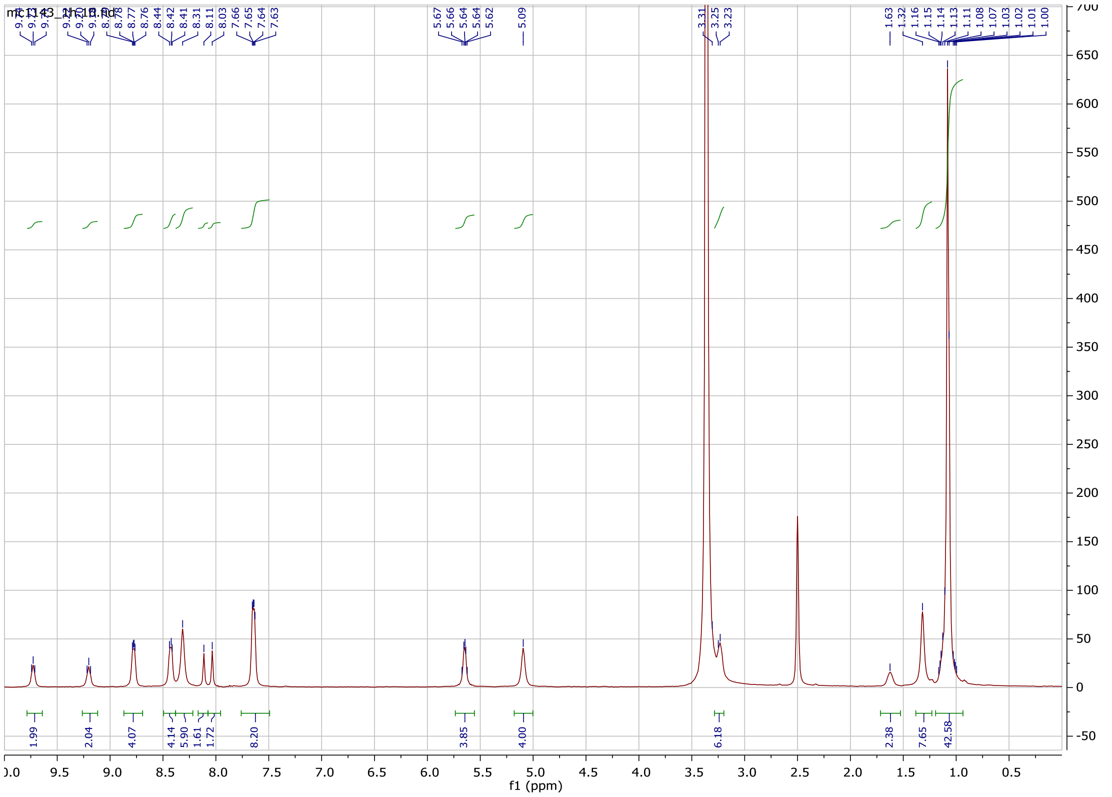

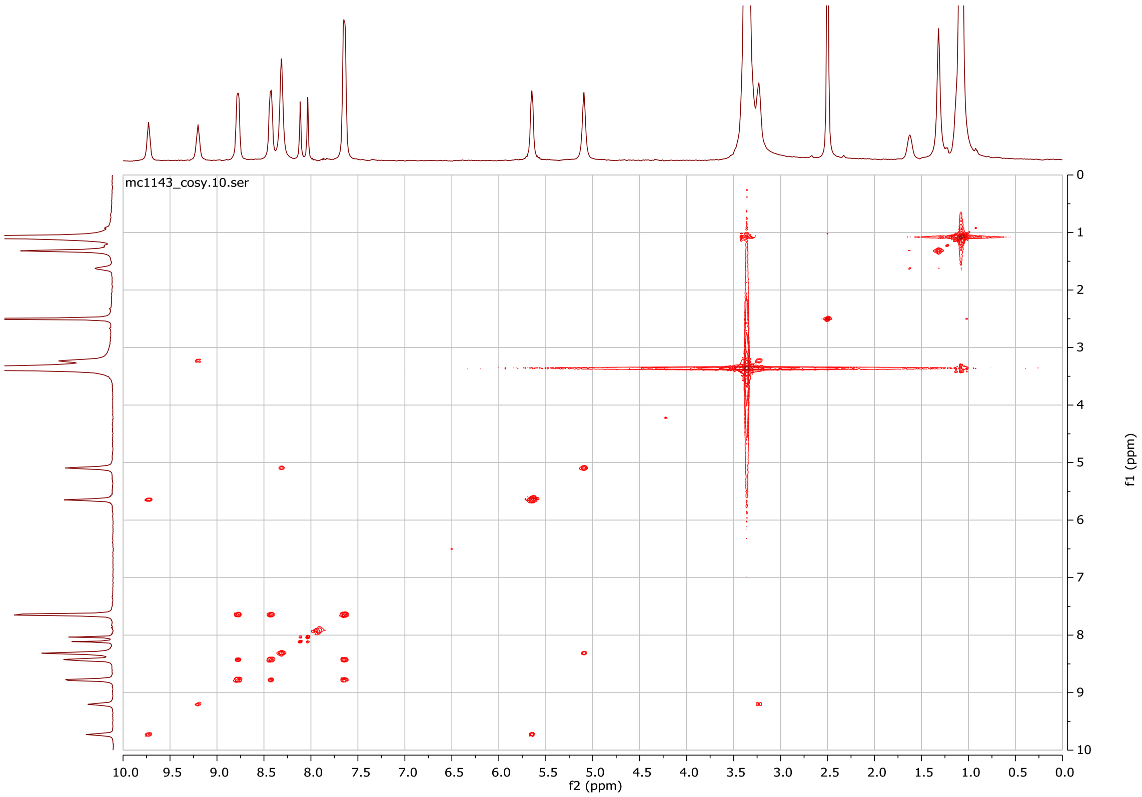
mc1143_cosy.10.ser
f2 (ppm)
f1 (ppm)

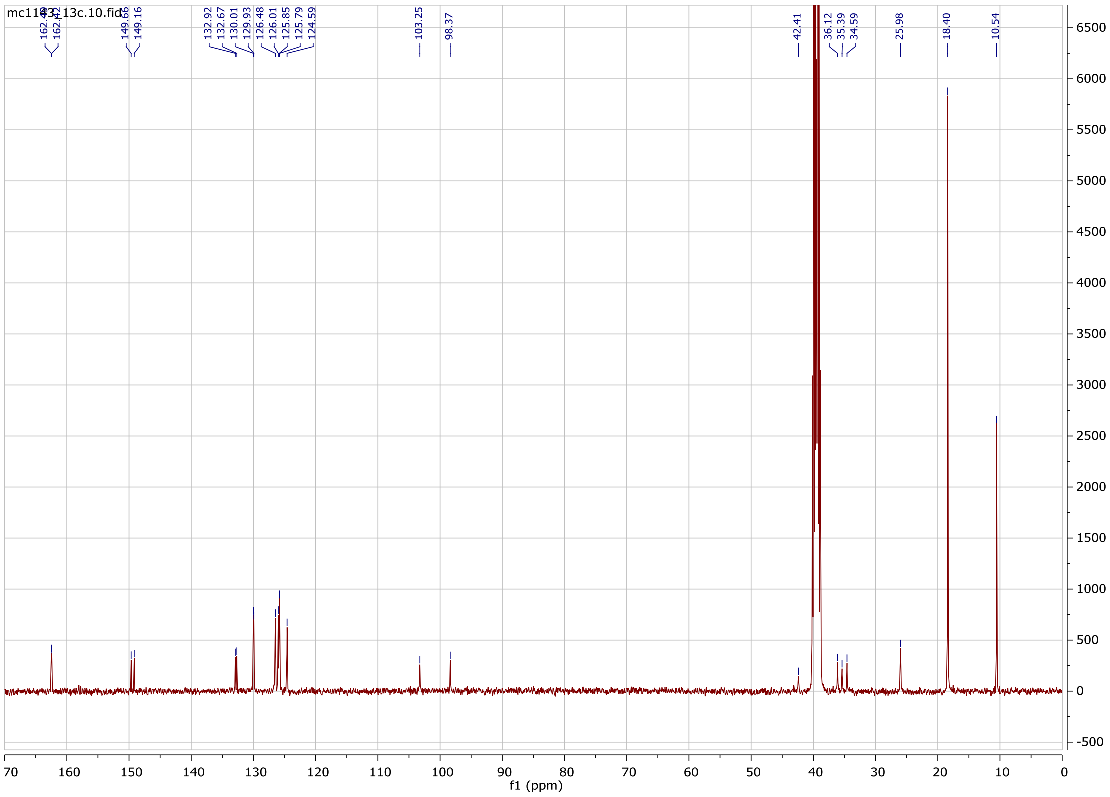
149.16
132.92
132.67
130.01
129.93
126.48
126.01
125.85
125.79
124.59
103.25
98.37
42.41
36.12
35.39
34.59
25.98
18.40
10.54
f1 (ppm)

DOSY-NMR (500MHz, TCE-d_2)

Dimer-NHBoc

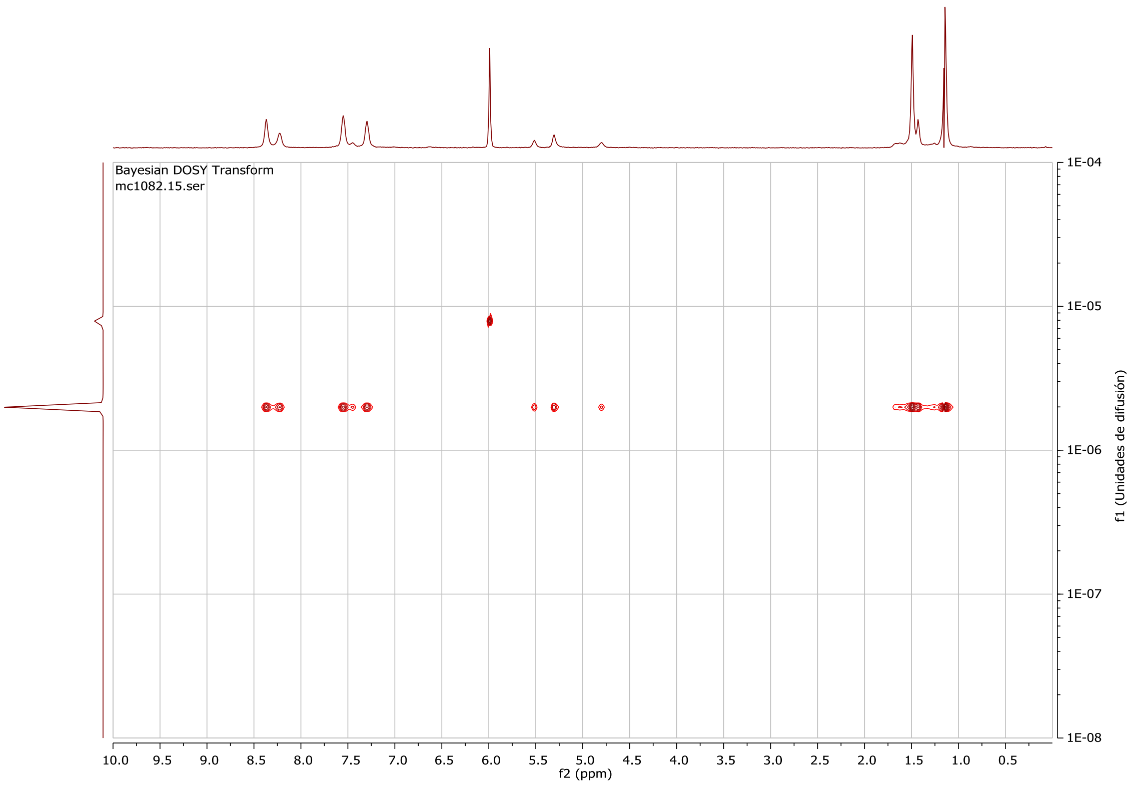

Trimer-NHBoc

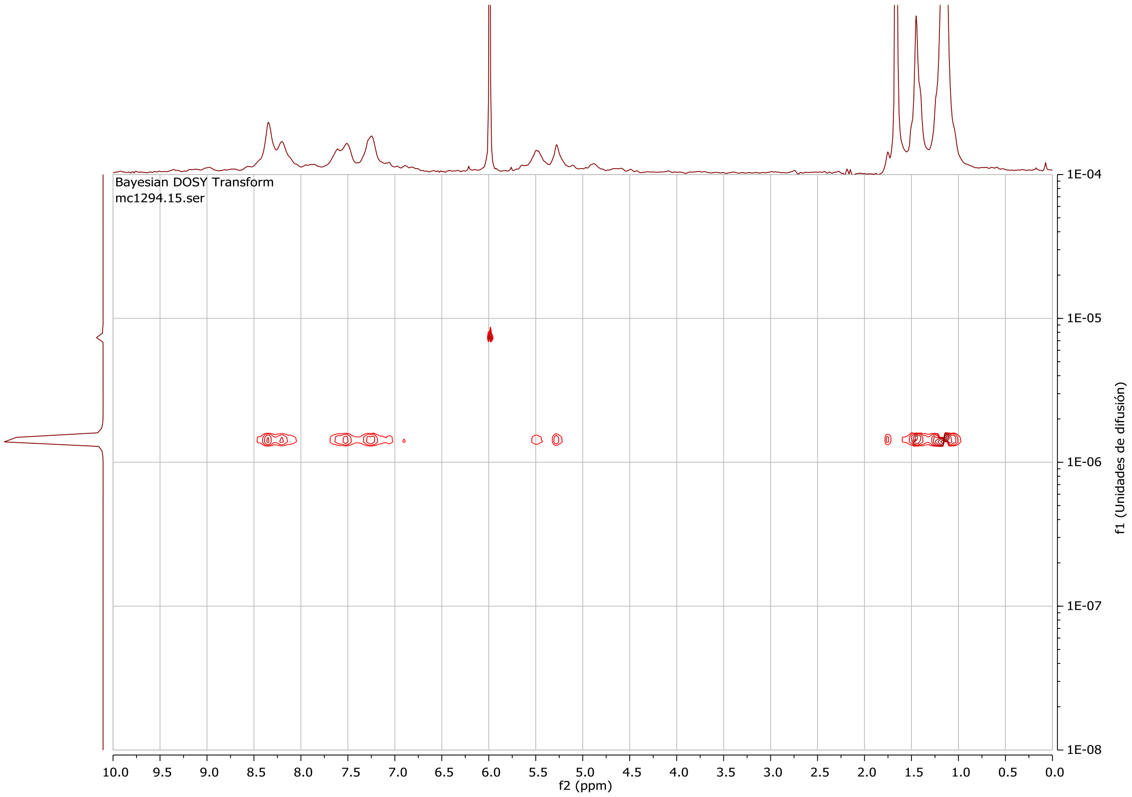

Tetramer-NHBoc

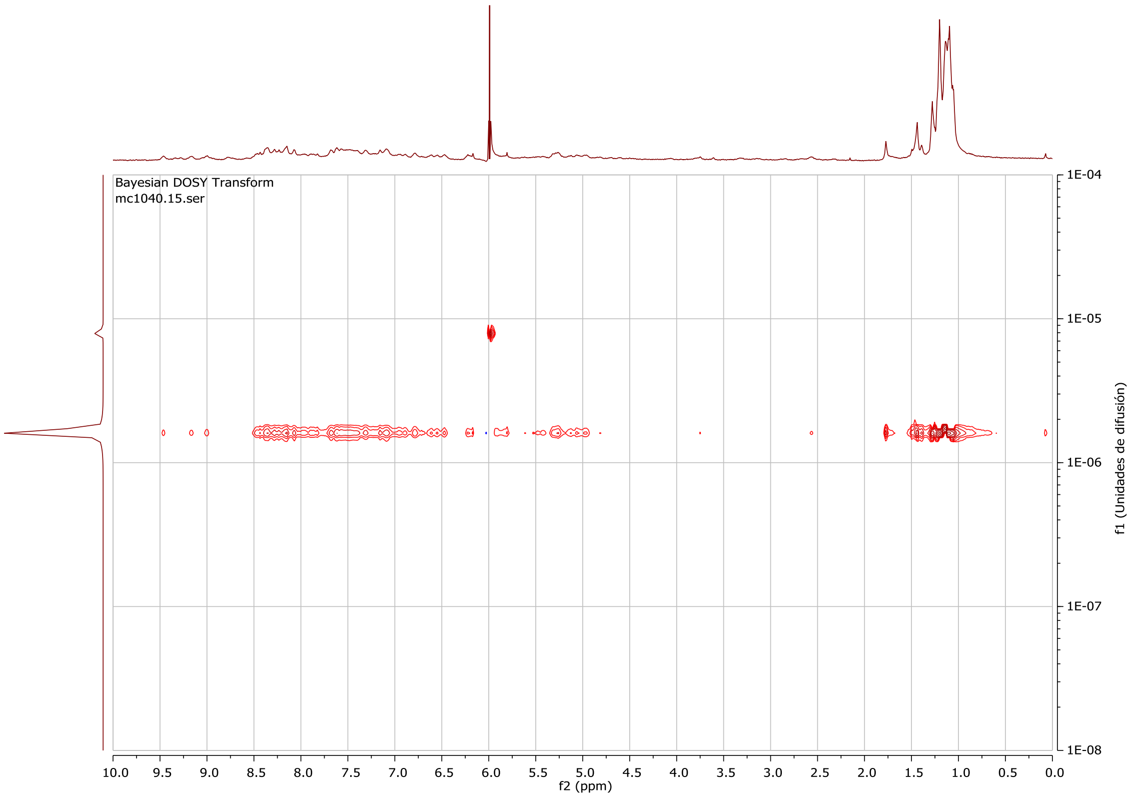

Pentamer-NHBoc

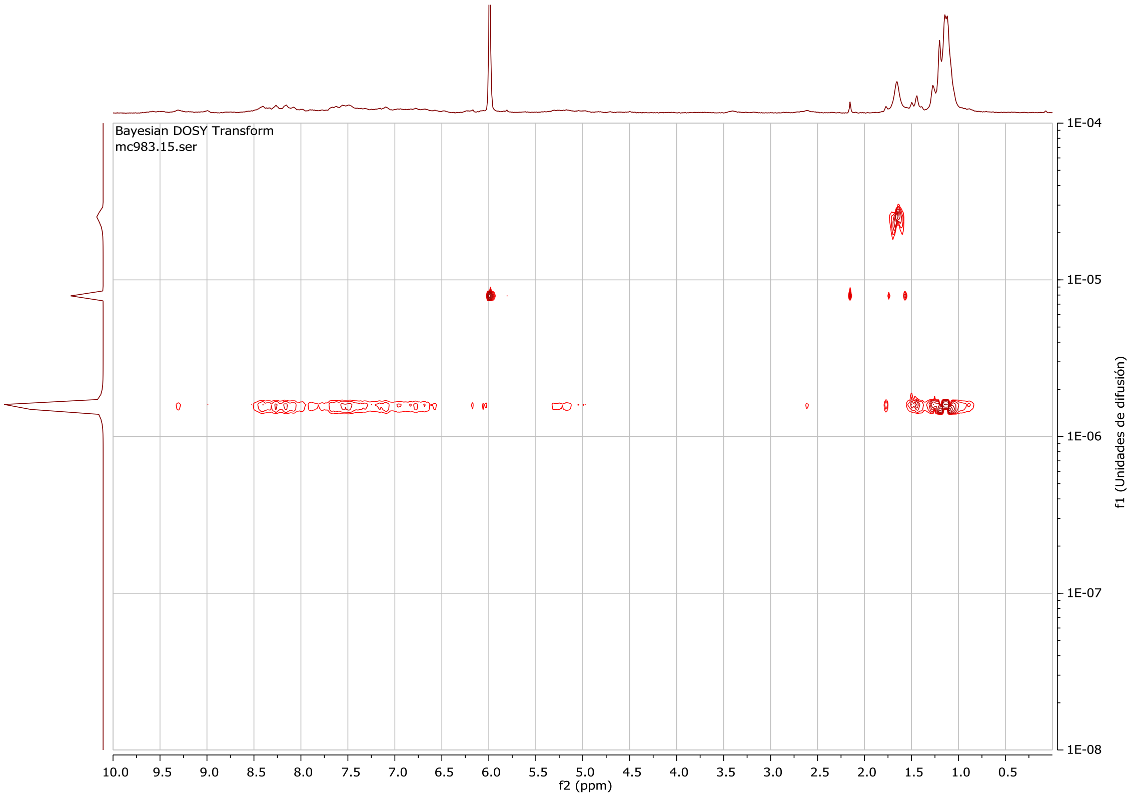

Pentamer-NHBoc (VT-NMR, 500MHz, TCE-d_2, N_2, sealed tube)

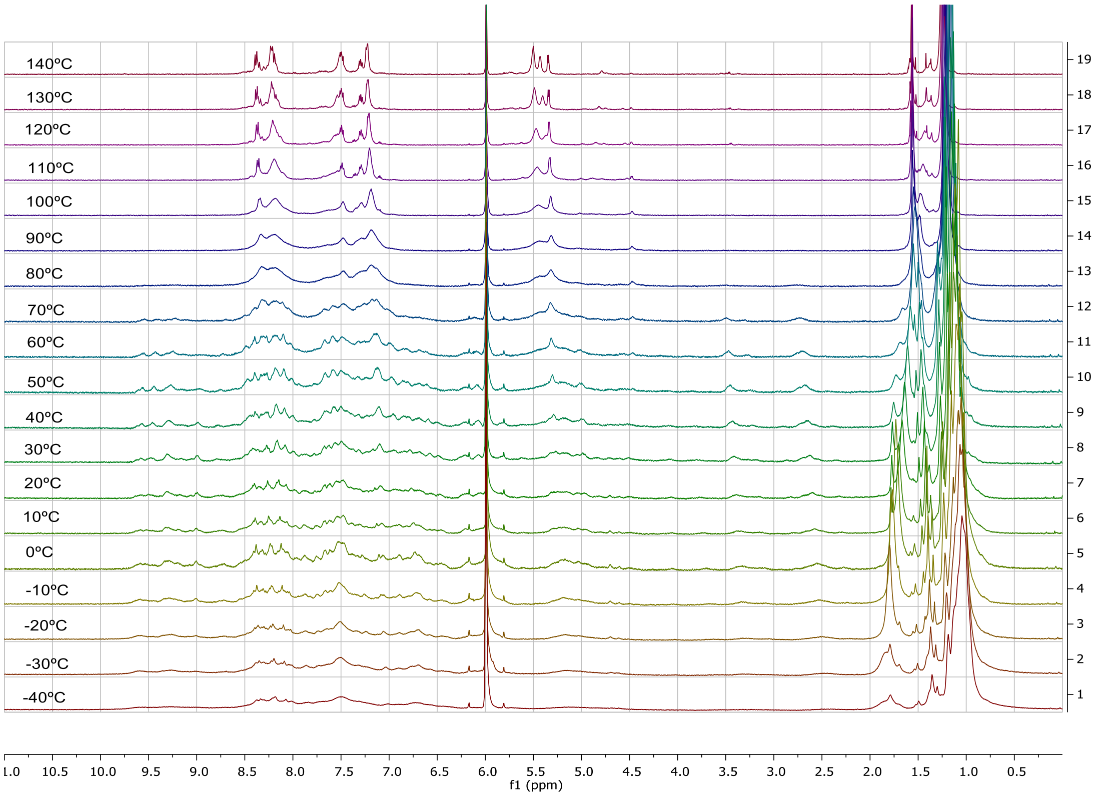

Pentamer-NHBoc (^{1}H and COSY-NMR, 500MHz, TCE-d_2, N_2, sealed tube, 140ºC)

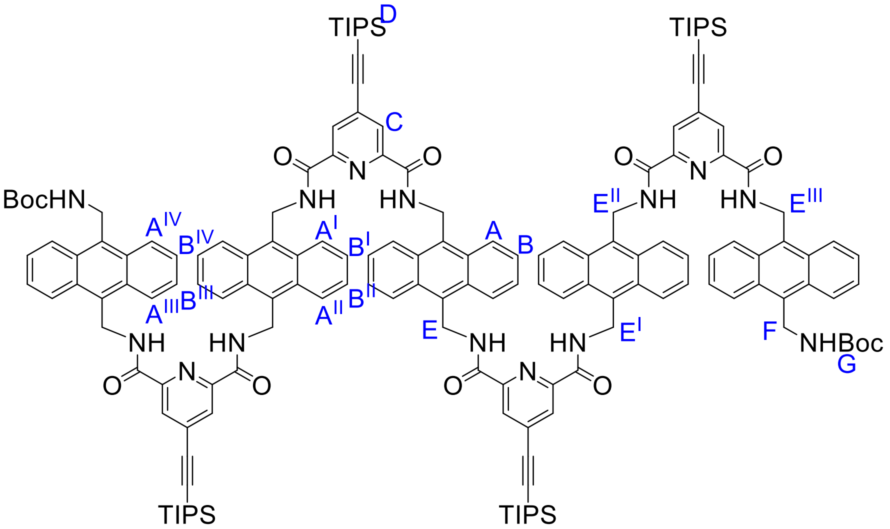

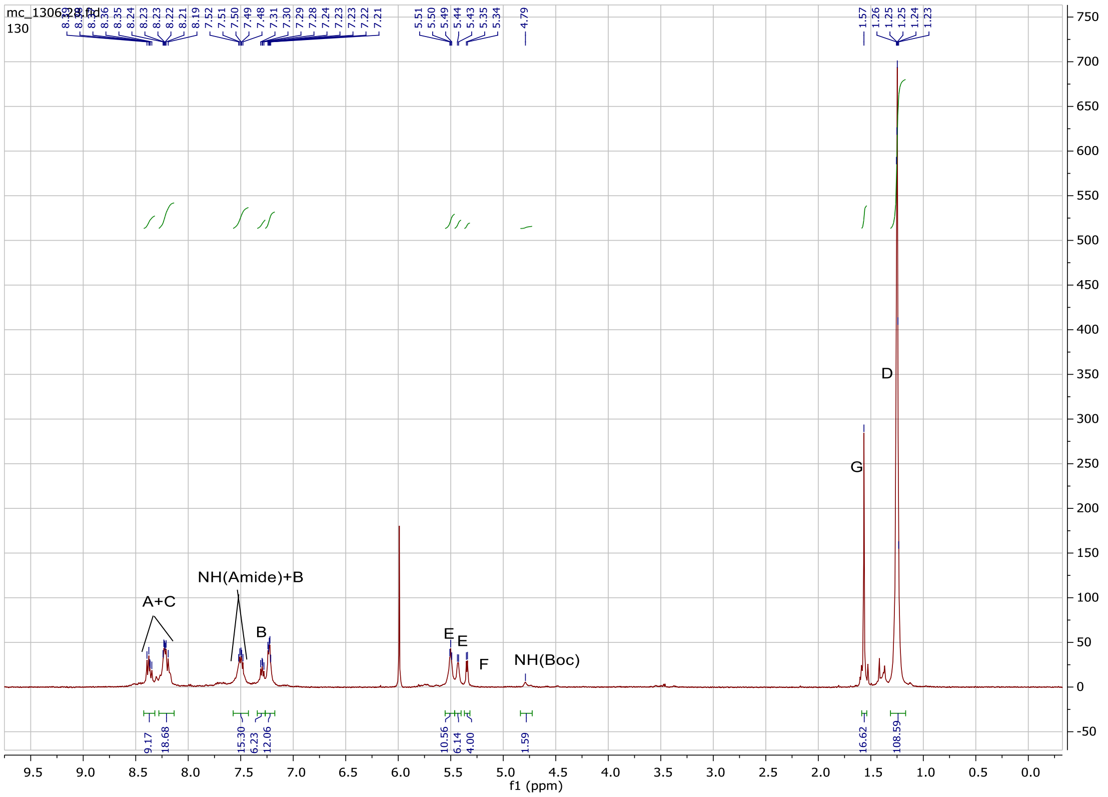
mc_1306.28.fid
130
A+C
NH(Amide)+B
B
E
E
F
NH(Boc)
G
D
f1 (ppm)

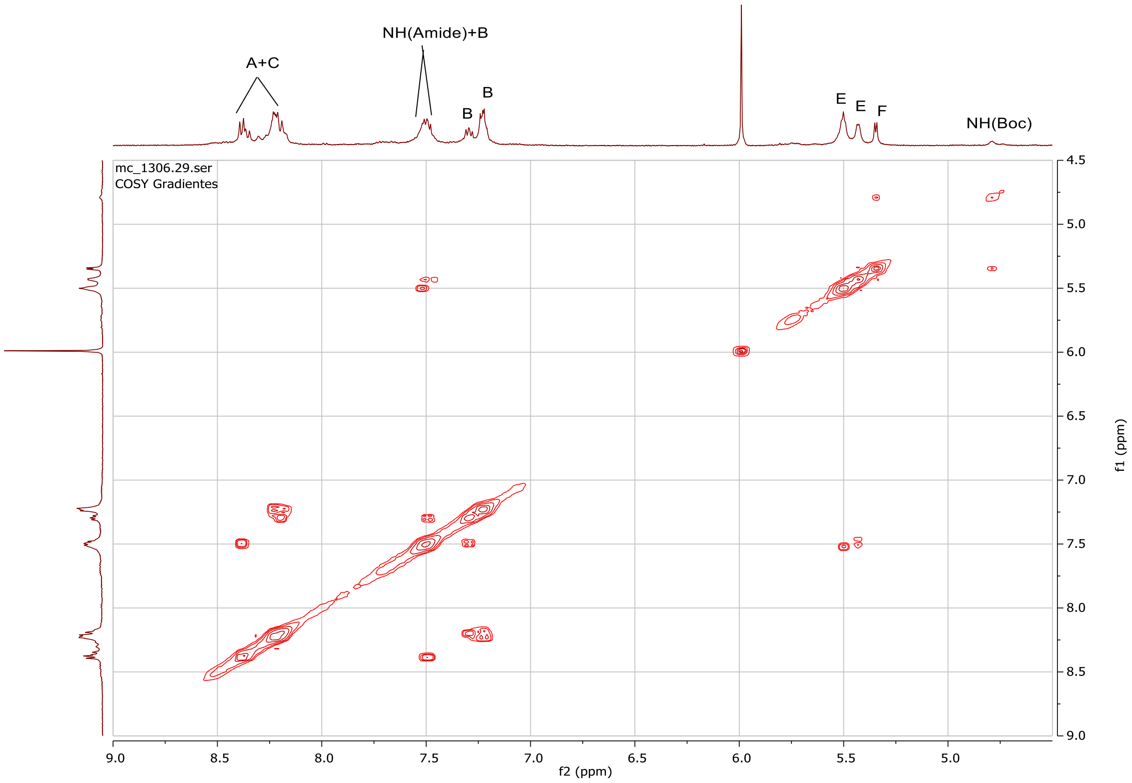
mc_1306.29.ser
COSY Gradientes
A+C
NH(Amide)+B
B
B
E
E
F
NH(Boc)
f2 (ppm)
f1 (ppm)

Supplementary Note 2: STM Break junction

An estimation of the electronic coupling strength (Γ) between the molecule and the electrodes has been obtained from a simple Breit-Wigner single-level tunneling model for transport (see manuscript) by using its numerical version:[3]

$$I(V) = n\frac{8e}{h}\alpha(1-\alpha)\Gamma\left[arctan\left(\frac{\alpha V_{bias}-\varepsilon_o}{\Gamma}\right) + arctan\left(\frac{(1-\alpha)V_{bias}+\varepsilon_o}{\Gamma}\right)\right]$$

where α is assumed to be 0.5, meaning a symmetrical coupling on both sides of the molecular junctions ($\alpha=\Gamma_R/\Gamma$, being $\Gamma=\Gamma_R+\Gamma_L$ where Γ_R and Γ_L are the electronic coupling of the molecule to the right and left electrodes respectively). ε_0 represents the level alignment between the molecular frontier orbital and the electrodes Fermi energy, which it is set to 0.11 eV from our computational calculations (see manuscript). V_{bias} is the voltage difference between the two junction electrodes.

Supplementary References

1. Gassensmith JJ*, et al.* Self-Assembly of Fluorescent Inclusion Complexes in Competitive Media Including the Interior of Living Cells. *J. Am. Chem. Soc.* **129**, 15054-15059 (2007).

2. Achmatowicz M, Hegedus LS, David S. Synthesis and Structural Studies of 5,12-Dioxocyclams Capped by 4-Substituted Pyridines Across the Amine Nitrogens. *J. Org. Chem.* **68**, 7661-7666 (2003).

3. Komoto Y, Fujii S, Nishino T, Kiguchi M. High electronic couplings of single mesitylene molecular junctions. *Beilstein J. Nanotechnol.* **6**, 2431–2437 (2015).
